# Making Mosquito Taxonomy Useful: A Stable Classification of Tribe Aedini that Balances Utility with Current Knowledge of Evolutionary Relationships

**DOI:** 10.1371/journal.pone.0133602

**Published:** 2015-07-30

**Authors:** Richard C. Wilkerson, Yvonne-Marie Linton, Dina M. Fonseca, Ted R. Schultz, Dana C. Price, Daniel A. Strickman

**Affiliations:** 1 Department of Entomology, National Museum of Natural History, Smithsonian Institution, Washington DC, United States of America; 2 Walter Reed Biosystematics Unit, Museum Support Center, Smithsonian Institution, Suitland, Maryland, United States of America; 3 Department of Entomology, Walter Reed Army Institute of Research, Silver Spring, Maryland, United States of America; 4 Faculty of Preventative Medicine and Biometrics, Uniformed Services University of the Health Sciences, Bethesda, Maryland, United States of America; 5 Entomology Department, School of Environmental and Biological Sciences, Rutgers University, New Brunswick, New Jersey, United States of America; 6 Global Health Program, Bill and Melinda Gates Foundation, Seattle, Washington, United States of America; The National Orchid Conservation Center of China; The Orchid Conservation & Research Center of Shenzhen, CHINA

## Abstract

The tribe Aedini (Family Culicidae) contains approximately one-quarter of the known species of mosquitoes, including vectors of deadly or debilitating disease agents. This tribe contains the genus *Aedes*, which is one of the three most familiar genera of mosquitoes. During the past decade, Aedini has been the focus of a series of extensive morphology-based phylogenetic studies published by Reinert, Harbach, and Kitching (RH&K). Those authors created 74 new, elevated or resurrected genera from what had been the single genus *Aedes*, almost tripling the number of genera in the entire family Culicidae. The proposed classification is based on subjective assessments of the “number and nature of the characters that support the branches” subtending particular monophyletic groups in the results of cladistic analyses of a large set of morphological characters of representative species. To gauge the stability of RH&K’s generic groupings we reanalyzed their data with unweighted parsimony jackknife and maximum-parsimony analyses, with and without ordering 14 of the characters as in RH&K. We found that their phylogeny was largely weakly supported and their taxonomic rankings failed priority and other useful taxon-naming criteria. Consequently, we propose simplified aedine generic designations that 1) restore a classification system that is useful for the operational community; 2) enhance the ability of taxonomists to accurately place new species into genera; 3) maintain the progress toward a natural classification based on monophyletic groups of species; and 4) correct the current classification system that is subject to instability as new species are described and existing species more thoroughly defined. We do not challenge the phylogenetic hypotheses generated by the above-mentioned series of morphological studies. However, we reduce the ranks of the genera and subgenera of RH&K to subgenera or informal species groups, respectively, to preserve stability as new data become available.

## Introduction

The *Catalog of the Mosquitoes of the World*, published in 1977 by Knight and Stone [1] and its three supplements [2–5], which were based on an earlier 1959 catalog [6] and supplements [7–11], are the primary reference point for all modern mosquito systematics. These catalogs summarize the nomenclatorial organization of the entire Culicidae family and list all references that established those names. The family Culicidae includes 3,601 described species and subspecies (www.mosquitocatalog.org accessed 22 Jan., 2014), a number that steadily increases due to the intense medical and veterinary importance of mosquitoes. The family is organized into two subfamilies, the Anophelinae (482 species) and the Culicinae (3,119 species). Aedini, with 1,261 species, is the largest of the 11 tribes within Culicinae. The genus *Aedes* in the tribe Aedini is one of three best-known genera of mosquitoes (along with *Culex* and *Anopheles*) since many of its species are important vectors of arboviruses and pathogens (Table 1). Because of its medical importance, *Aedes* is recognized by thousands of researchers and operators concerned with mosquito control, public health, and veterinary health.

**Table 1:** Name combinations used since 1977 for selected mosquitoes belonging to Tribe Aedini with associated human pathogens and invasive (established, spreading) or non-native (established, not spreading) behaviors. Names proposed herein are in bold typeface; previous name combinations follow in normal typeface.

SPECIES	ASSOCIATED HUMAN PATHOGENS / INVASIVE AND NON-NATIVE SPECIES
***Aedes* (*Aedes*) *cinereus* Meigen** *	EEEV^[^ ^12^ ^]^, HJV^[^ ^13^ ^]^, JCV^[^ ^13^ ^]^,
*Aedes cinereus* Meigen	**TAHV**, WNV^[^ ^14^ ^]^
***Aedes* (*Aedimorphus*) *abnormalis* (Theobald)** *	**SFV**, **WSLV**
*'Aedes'* (*'Aedimorphus'*) *abnormalis* (Theobald)	
*Aedimorphus abnormalis* (Theobald)	
***Aedes* (*Aedimorphus*) *cumminsii* (Theobald)** *	RVFV^[^ ^15^ ^]^, **SPOV**
*'Aedes'* (*'Aedimorphus'*) *cumminsii* (Theobald)	
*Aedimorphus cumminsii* (*Theobald*)	
***Aedes* (*Aedimorphus*) *dalzieli* (Theobald)** *	BBKV^[^ ^15^ ^]^, BOUV^[^ ^15^ ^]^, CHIKV^[^ ^15^ ^]^,
*'Aedes'* (*'Aedimorphus'*) *dalzieli* (Theobald)	**KEDV**, **NDOV**, **PGAV**, RVFV^[^ ^15^ ^]^, **WSLV, ZIKAV**
*Aedimorphus dalzieli* (Theobald)	
***Aedes* (*Aedimorphus*) *dentatus* (Theobald)** *	**ORUV**, RVFV^[^ ^16^ ^]^, **WSLV**
*'Aedes'* (*'Aedimorphus'*) *dentatus* (Theobald)	
*Aedimorphus dentatus* (Theobald)	
***Aedes* (*Aedimorphus*) *fowleri* (de Charmoy)** *	**PGAV**, RVFV^+^ ^[^ ^17^ ^]^
*'Aedes'* (*'Aedimorphus'*) *fowleri* (de Charmoy)	
*Aedimorphus fowleri* (de Charmoy)	
***Aedes* (*Aedimorphus*) *hirsutus* (Theobald)** *	NRIV^[^ ^18^ ^]^
*'Aedes'* (*'Aedimorphus'*) *hirsutus* (Theobald)	
*Aedimorphus hirsutus* (Theobald)	
***Aedes* (*Aedimorphus*) *mediolineatus* (Theobald)** *	**WSLV**
*'Aedes'* (*'Aedimorphus'*) *mediolineatus* (Theobald)	
*Aedimorphus mediolineatus* (Theobald)	
***Aedes* (*Aedimorphus*) *natrionius* Edwards** *	**UGSV**
*'Aedes'* (*'Aedimorphus'*) *natronius* Edwards	
*Aedimorphus natronius* (Edwards)	
***Aedes* (*Aedimorphus*) *ochraceus* (Theobald)** *	RVFV^[^ ^15^ ^]^, WSLV^[^ ^15^ ^]^
*'Aedes'* (*'Aedimorphus'*) *ochraceus* (Theobald)	
*Aedimorphus ochraceus* (Theobald)	
***Aedes* (*Aedimorphus*) *vexans* (Meigen)** *	**TAHV**, **TVTV**, WEE^+^ ^[^ ^19^ ^]^, WNV^[^ ^14^ ^,^ ^15^ ^]^,
*'Aedes'* (*'Aedimorphus'*) *vexans* (Meigen)	Banna^[^ ^20^ ^]^, Chaoyang^[^ ^21^ ^]^, Potosi^[^ ^22^ ^]^
*Aedimorphus vexans* (Meigen)	
***Aedes* (*Catageiomyia*) *argenteopunctatus* (Theobald)**	**NRIV**, **SFV**
*Aedes* (*Aedimorphus*) *argenteopunctatus* (Theobald)*	
*'Aedes'* (*'Aedimorphus'*) *argenteopunctatus* (Theobald)	
*Catageiomyia argenteopunctata* (Theobald)	
***Aedes* (*Catageiomyia*) *minutus* (Theobald)**	**KEDV**
*Aedes* (*Aedimorphus*) *minutus* (Theobald)*	
*'Aedes'* (*'Aedimorphus'*) *minutus* (Theobald)	
*Catageiomyia minuta* (Theobald)	
***Aedes* (*Catageiomyia*) *tarsalis* (Newstead)**	**KEDV**, **PGAV**, **WSLV**
*Aedes* (*Aedimorphus*) *tarsalis* (Newstead)*	
*'Aedes'* (*'Aedimorphus'*) *tarsalis* (Newstead)	
*Catageiomyia tarsalis* (Newstead)	
***Aedes* (*Diceromyia*) *furcifer* (Edwards)** *	**BOUV**, CHIK^[^ ^23^ ^]^, RVFV^[^ ^15^ ^]^,
*Diceromyia furcifer* (Edwards)	YF^[^ ^24^ ^]^
***Aedes* (*Diceromyia*) *taylori* Edwards** *	CHIK^[^ ^23^ ^]^, YFV^[^ ^24^ ^]^
*Diceromyia taylori* (Edwards)*	
***Aedes* (*Downsiomyia*) *harinasutai* Knight**	*W*. *bancrofti* ^[^ ^25^ ^]^
*Aedes* (*Finlaya*) *harinasutai* Knight*	
*Downsiomyia harinasutai* (Knight)	
***Aedes* (*Downsiomyia*) *niveus* (Ludlow)**	**DENV,** *W*. *bancrofti* ^[^ ^26^ ^]^
*Aedes* (*Finlaya*) *niveus* (Ludlow)*	
*Downsiomyia nivea* (Ludlow)	
**Ae*des* (*Finlaya*) *fijiensis* Marks** *	*W*. *bancrofti* ^[^ ^27^ ^]^
*Finlaya fijiensis* (Marks)	
***Aedes* (*Finlaya*) *kochi* (Dönitz)** *	*W*. *bancrofti* ^[^ ^28^ ^]^
*Ochlerotatus* (*Finlaya*) *kochi* (Dönitz)	
*Finlaya kochi* (Dönitz)	
***Aedes* (*Finlaya*) *poicilius* (Theobald)** *	*W*. *bancrofti* ^[^ ^29^ ^]^
*Ochlerotatus* (*Finlaya*) *poicilius* (Theobald)	
*Finlaya poicilia* Theobald	
***Aedes* (*Fredwardsius*) *vittatus* (Bigot)**	**BBKV**, **NRIV**, **PGAV**, **SFV**
*Aedes* (*Stegomyia*) *vittatus* (Bigot)*	
*Fredwardsius vittatus* (Bigot)	
***Aedes* (*Georgecraigius*) *epactius* Dyar & Knab**	WNV^[^ ^14^ ^]^
*Aedes* (*Ochlerotatus*) *epactius* Dyar & Knab*	
*Ochlerotatus* (*Ochlerotatus*) *epactius* (Dyar & Knab)	
*'Ochlerotatus'* (*'Ochlerotatus'*) *epactius* (Dyar & Knab)	
*Georgecraigius* (*Georgecraigius*) *epactius* (Dyar & Knab)	
***Aedes* (*Georgecraigius*) *atropalpus* (Coquillett)**	Invasive^[^ ^30^ ^]^, WNV^[^ ^14^ ^]^
*Aedes* (*Ochlerotatus*) *atropalpus* (Coquillett)*	
*Ochlerotatus* (*Ochlerotatus*) *atropalpus* (Coquillett)	
*'Ochlerotatus'* (*'Ochlerotatus'*) *atropalpus* (Coquillett)	
*Georgecraigius* (*Georgecraigius*) *atropalpus* (Coquillett)	
***Aedes* (*Hopkinsius*) *ingrami* (Edwards)**	**UGSV**
*Aedes* (*Finlaya*) *ingrami* Edwards*	
*Ochlerotatus* (*Finlaya*) *ingrami* (Edwards)	
*'Ochlerotatus'* (*'Finlaya'*) *ingrami* (Edwards)	
*Hopkinsius* (*Hopkinsius*) *ingrami* (Edwards)	
***Aedes* (*Howardina*) *septemstriatus* Dyar & Knab** *	**WYOV**
*Ochlerotatus* (*Howardina*) *septemstriatus* (Dyar & Knab)	
*Howardina septemstriata* (Dyar & Knab)	
***Aedes* (*Howardina*) *sexlineatus* (Theobald)** *	**WYOV**
*Ochlerotatus* (*Howardina*) *sexlineatus* (Theobald)	
*Howardina sexlineata* (Theobald)	
***Aedes* (*Hulecoeteomyia*) *japonicus* (Theobald)**	Invasive^[^ ^30^ ^]^, CVV^[^ ^31^ ^]^, WNV^[^ ^14^ ^]^
*Aedes* (*Finlaya*) *japonicus* (Theobald)*	
*Ochlerotatus* (*Finlaya*) *japonicus* (Theobald)	
*'Ochlerotatus'* (*'Finlaya'*) *japonicus* (Theobald)	
*Hulecoeteomyia japonica* (Theobald)	
***Aedes* (*Hulecoeteomyia*) *koreicus* (Edwards)**	Invasive^[^ ^32^ ^]^
*Aedes* (*Finlaya*) *koreicus* (Edwards)*	
*Ochlerotatus* (*Finlaya*) *koreicus* Edwards	
*'Ochlerotatus'* (*'Finlaya'*) *koreicus* Edwards	
*Hulecoeteomyia koreica* (Edwards)	
***Aedes* (*Neomelaniconion*) *mcintoshi* Huang** *	NRIV^[^ ^15^ ^]^, MIDV^[^ ^33^ ^]^, RVFV^[^ ^34^ ^]^,
*Neomelaniconion mcintoshi* (Huang)	WSLV^[^ ^33^ ^]^
***Aedes* (*Neomelaniconion*) *palpalis* (Newstead)** *	RVFV^[^ ^15^ ^]^, **SFV**
*Neomelaniconion palpale* Newstead	
***Aedes* (*Neomelaniconion*) *circumluteolus* (Theobald)** *	**BUNV**, **LEBV**, **PGAV**, **RVFV**, **SPON**, **WSLV**
*Neomelaniconion circumluteolus* (Theobald)	
*Neomelaniconion circuluteolum* (Theobald)	
***Aedes* (*Neomelaniconion*) *lineatopennis* (Ludlow)** *	**WSLV**
*Neomelaniconion lineatopenne* (Ludlow)	
***Aedes* (*Ochlerotatus*) *abserratus* (Felt & Young)** *	**JCV**
*Ochlerotatus* (*Ochlerotatus*) *abserratus* (Felt & Young)	
*'Ochlerotatus'* (*'Ochlerotatus'*) *abserratus* (Felt & Young)	
*Ochlerotatus* (subgenus unassigned) *abserratus* (Felt & Young)	
*Ochlerotatus* (*Woodius*) *abserratus* (Felt & Young)	
***Aedes* (*Ochlerotatus*) *angustivittatus* Dyar & Knab** *	ILHV^[^ ^35^ ^]^, **VEEV**
*Ochlerotatus* (*Ochlerotatus*) *angustivittatus* (Dyar & Knab)	
*'Ochlerotatus'* (*'Ochlerotatus'*) *angustivittatus* (Dyar & Knab)	
***Aedes* (*Ochlerotatus*) *argyrothorax* (Bonne-Wepster & Bonne)** *	**WYOV**
*Aedes* (*Protomacleaya*) *argyrothorax* (Bonne-Wepster & Bonne)	
*Ochlerotatus* (*Protomacleaya*) *argyrothorax* (Bonne-Wepster & Bonne)	
*'Ochlerotatus'* (*'Protomacleaya'*) *argyrothorax* (Bonne-Wepster & Bonne)	
***Aedes* (*Ochlerotatus*) *atlanticus* Dyar & Knab** *	**EEEV**, **EYEV**, **TENV**, **TVTV**, WNV^[^ ^14^ ^]^
*Ochlerotatus* (*Ochlerotatus*) *atlanticus* (Dyar & Knab)	
*Ochlerotatus* (subgenus unassigned) *atlanticus* (Dyar & Knab)	
*Ochlerotatus* (*Protoculex*) *atlanticus* (Dyar & Knab)	
***Aedes* (*Ochlerotatus*) *bancroftianus* (Edwards)**	**BAMV**
*Aedes* (*Pseudoskusea*) *bancroftianus* Edwards*	
*Ochlerotatus* (*Pseudoskusea*) *bancroftianus* (Edwards)	
*'Ochlerotatus'* (*'Pseudoskusea'*) *bancroftianus* (Edwards)	
*Pseudoskusea bancroftiana* (*Edwards*)	
***Aedes* (*Ochlerotatus*) *caballus* (Theobald)** *	**RVFV**, **WSLV**
*Ochlerotatus* (*Ochlerotatus*) *caballus* (Theobald)	
*'Ochlerotatus'* (*'Ochlerotatus'*) *caballus* (Theobald)	
*Ochlerotatus* (*subgenus unassigned*) *caballus* (Theobald)	
*Ochlerotatus* (*Juppius*) *caballus* (Theobald)	
***Aedes* (*Ochlerotatus*) *canadensis* (Theobald)** *	EEEV^[^ ^12^ ^]^, HJV^[^ ^36^ ^]^, JCV^[^ ^13^ ^]^,
*Ochlerotatus* (*Ochlerotatus*) *canadensis* (Theobald)	RVFV^+^ ^[^ ^37^ ^]^, WNV^[^ ^19^ ^]^
*'Ochlerotatus'* (*'Ochlerotatus'*) *canadensis* (Theobald)	
*Ochlerotatus* (*subgenus unassigned*) *canadensis* (Theobald)	
*Ochlerotatus* (*Culicada*) *canadensis* (Theobald)	
***Aedes* (*Ochlerotatus*) *cantans* (Meigen)** *	**TAHV, WNV **
*Ochlerotatus* (*Ochlerotatus*) *cantans* (Meigen)	
*'Ochlerotatus'* (*'Ochlerotatus'*) *cantans* (Meigen)	
*Ochlerotatus* (subgenus unassigned) *cantans* (Meigen)	
*Ochlerotatus* (*Woodius*) *cantans* (Meigen)	
***Aedes* (*Ochlerotatus*) *cantator* (Coquillett)** *	CVV^[^ ^31^ ^]^, EEEV^[^ ^36^ ^]^, HJV^[^ ^36^ ^]^, **JCV**,
*Ochlerotatus* (*Ochlerotatus*) *cantator* (Coquillett)	RVFV^+^ ^[^ ^37^ ^]^, WNV^[^ ^14^ ^]^
*'Ochlerotatus'* (*'Ochlerotatus'*) *cantator* (Coquillett)	
*Ochlerotatus* (subgenus unassigned) *cantato*r (Coquillett)	
***Aedes* (*Ochlerotatus*) *caspius* (Pallas)** *	**TAHV**, WNV^[^ ^38^ ^]^
*Ochlerotatus* (*Ochlerotatus*) *caspius* (Pallas)	
*'Ochlerotatus'* (*'Ochlerotatus'*) *caspius* (Pallas)	
*Ochlerotatus* (subgenus unassigned) *caspius* (Pallas)	
***Aedes* (*Ochlerotatus*) *communis* (de Geer)** *	**LACV**, **JCV**, **TAHV**
*Ochlerotatus* (*Ochlerotatus*) *communis* (de Geer)	
*Ochlerotatus* (subgenus unassigned) *communis* de Geer	
***Aedes* (*Ochlerotatus*) *condolescens* Dyar & Knab** *	WNV^[^ ^14^ ^]^
*'Ochlerotatus'* (*'Ochlerotatus'*) *condolescens* (Dyar & Knab)	
*Ochlerotatus* (*Ochlerotatus*) *condolescens* (Dyar & Knab)	
***Aedes* (*Ochlerotatus*) *detritus* Haliday** *	TAHV^[^ ^39^ ^]^
*Ochlerotatus* (*Ochlerotatus*) *detritus* (Haliday)	
*'Ochlerotatus'* (*'Ochlerotatus'*) *detritus* (Haliday)	
*Ochlerotatus* (subgenus unassigned) *detritus* (Haliday)	
***Aedes* (*Ochlerotatus*) *diantaeus* Howard, Dyar & Knab** *	**TAHV**
*Aedes* (*Ochlerotatus*) *diantaeus* Howard, Dyar & Knab*	
*Ochlerotatus* (*Ochlerotatus*) *diantaeus* (Howard, Dyar & Knab)	
*'Ochlerotatus'* (*'Ochlerotatus'*) *diantaeus* (Howard, Dyar & Knab)	
*Ochlerotatus* (subgenus unassigned) *diantaeus* (Howard, Dyar & Knab)	
*Ochlerotatus* (*Woodius*) *diantaeus* (Howard, Dyar & Knab)	
***Aedes* (*Ochlerotatus*) *dorsalis* (Meigen)** *	WEE^[^ ^40^ ^]^, WNV^[^ ^14^ ^]^, Banna^[^ ^20^ ^]^,
*Ochlerotatus* (*Ochlerotatus*) *dorsalis* (Meigen)	Liaoning^[^ ^41^ ^]^
*Ochlerotatus* (subgenus unassigned) *dorsalis* (Meigen)	
***Aedes* (*Ochlerotatus*) *dupreei* (Coquillett)** *	WNV^[^ ^14^ ^]^
*Ochlerotatus* (*Ochlerotatus*) *dupreei* (Coquillett)	
*'Ochlerotatus'*) *dupreei* (Coquillett)	
*Ochlerotatus* (*Protoculex*) *dupreei* (Coquillett)	
***Aedes* (*Ochlerotatus*) *excrucians* (Walker)** *	RVFV^+^ ^[^ ^37^ ^]^
*Ochlerotatus* (*Ochlerotatus*) *excrucians* (Walker)	
*'Ochlerotatus'* (*'Ochlerotatus'*) *excrucians* (Walker)	
*Ochlerotatus* (subgenus unassigned) *excrucians* (Walker)	
***Aedes* (*Ochlerotatus*) *fitchii* (Felt & Young)** *	WNV^[^ ^14^ ^]^
*Ochlerotatus* (*Ochlerotatus*) *fitchii* (Felt & Young)	
*'Ochlerotatus'* (*'Ochlerotatus'*) *fitchii* (Felt & Young)	
*Ochlerotatus* (subgenus unassigned) *fitchii* (Felt & Young)	
***Aedes* (*Ochlerotatus*) *fulvus* (Wiedemann)** *	**EEEV, WNV, WYOV**
*Ochlerotatus* (*Ochlerotatus*) *fulvus* (Wiedemann)	
*Ochlerotatus* (subgenus unassigned) *fulvus* (Wiedemann)	
*Ochlerotatus* (*Chrysoconops*) *fulvus* (Wiedemann)	
***Aedes* (*Ochlerotatus*) *grossbecki* Dyar & Knab** *	WNV^[^ ^14^ ^]^
*Ochlerotatus* (*Ochlerotatus*) *grossbecki* (Dyar & Knab)	
*'Ochlerotatus'* (*'Ochlerotatus'*) *grossbecki* (Dyar & Knab)	
*Ochlerotatus* (subgenus unassigned) *grossbecki* (Dyar & Knab)	
***Aedes* (*Ochlerotatus*) *infirmatus* Dyar & Knab** *	EEEV^[^ ^42^ ^]^, Keystone^[^ ^42^ ^]^,
*Ochlerotatus* (*Ochlerotatus*) *infirmatus* (Dyar & Knab)	Tensaw^[^ ^42^ ^]^, **TVTV**, WNV^[^ ^14^ ^]^
*Ochlerotatus* (subgenus unassigned) *infirmatus* (Dyar & Knab)	
***Aedes* (*Ochlerotatus*) *melanimon* Dyar** *	**SLEV**, WEEV^[^ ^19^ ^]^, WNV^[^ ^14^ ^]^
*Ochlerotatus* (*Ochlerotatus*) *melanimon* (Dyar)	
*'Ochlerotatus'* (*'Ochlerotatus'*) *melanimon* (Dyar)	
*Ochlerotatus* (subgenus unassigned) *melanimon* (Dyar)	
***Aedes* (*Ochlerotatus*) *mitchellae* (Dyar)** *	**EEEV**, **TENV**
*Ochlerotatus* (*Ochlerotatus*) *mitchellae* (Dyar)	
*'Ochlerotatus'* (*'Ochlerotatus'*) *mitchellae* (Dyar)	
*Ochlerotatus* (*Culicelsa*) *mitchellae* (Dyar)	
***Aedes* (*Ochlerotatus*) *nigromaculis* (Ludlow)** *	WNV^[^ ^14^ ^]^
*Ochlerotatus* (*Ochlerotatus*) *nigromaculis* (Ludlow)	
*'Ochlerotatus'* (*'Ochlerotatus'*) *nigromaculis* (Ludlow)	
*Ochlerotatus* (*Culicelsa*) *nigromaculis* (Ludlow)	
***Aedes* (*Ochlerotatus*) *normanensis* (Taylor)** *	**BAMV**, **GGV**, **MVEV**, **RRV**, **SINV**
*Ochlerotatus* (*Ochlerotatus*) *normanensis* (Taylor)	
*'Ochlerotatus'* (*'Ochlerotatus'*) *normanensis* (Taylor)	
*Ochlerotatus* (subgenus unassigned) *normanensis* (Taylor)	
***Aedes* (*Ochlerotatus*) *provocans* (Walker)** *	WNV^[^ ^14^ ^]^ ** **
*Ochlerotatus* (*Ochlerotatus*) *provocans* (Walker)	
*Ochlerotatus* (*Rusticoidus*) *provocans* (Walker)	
***Aedes* (*Ochlerotatus*) *punctor* (Kirby)** *	**BATV**
*Ochlerotatus* (*Ochlerotatus*) *punctor* (Kirby)	
*'Ochlerotatus'* (*'Ochlerotatus'*) *punctor* (Kirby)	
*Ochlerotatus* (subgenus unassigned) *punctor* (Kirby)	
***Aedes* (*Ochlerotatus*) *scapularis* (Rondani)** *	ILHV^[^ ^43^ ^]^, **SLEV**, **VEEV**, **WYOV,** YFV^[^ ^44^ ^]^,
*Ochlerotatus* (*Ochlerotatus*) *scapularis* (Rondani)*Ochlerotatus* (subgenus unassigned) *scapularis* (Rondani)	*W*. *bancrofti* ^[^ ^44^ ^]^
***Aedes* (*Ochlerotatus*) *serratus* (Theobald)** *	**SLEV**, **VEEV**, **WYOV**
*Ochlerotatus* (*Ochlerotatus*) *serratus* (Theobald)	
*'Ochlerotatus'* (*'Ochlerotatus'*) *serratus* (Theobald)	
*Ochlerotatus* (*Protoculex*) *serratus* (Theobald)	
***Aedes* (*Ochlerotatus*) *sollicitans* (Walker)** *	CVV^[^ ^31^ ^]^, **EEEV**, RVFV^+^ ^[^ ^37^ ^]^,
*Ochlerotatus* (*Ochlerotatus*) *sollicitans* (Walker)	**VEEV**, WNV^[^ ^14^ ^]^
*Ochlerotatus* (subgenus unassigned) *sollicitans* (Walker)	
*Ochlerotatus* (*Culicelsa*) *sollicitans* (Walker)	
***Aedes* (*Ochlerotatus*) *squamiger* (Coquillett)** *	WNV^[^ ^14^ ^]^
*Ochlerotatus* (*Ochlerotatus*) *squamiger* (Coquillett)	
*'Ochlerotatus'* (*'Ochlerotatus'*) *squamiger* (Coquillett)	
*Ochlerotatus* (subgenus unassigned) *squamiger* (Coquillett)	
***Aedes* (*Ochlerotatus*) *sticticus* (Meigen)** *	**EEEV**, **JCV**, **TAHV**, WNV^[^ ^14^ ^]^
*Ochlerotatus* (*Ochlerotatus*) *sticticus* (Meigen)	
*'Ochlerotatus'* (*'Ochlerotatus'*) *sticticus* (Meigen)	
*Ochlerotatus* (subgenus unassigned) *sticticus* (Meigen)	
***Aedes* (*Ochlerotatus*) *stimulans* (Walker)** *	**JCV**, WNV^[^ ^14^ ^]^
*Ochlerotatus* (*Ochlerotatus*) *stimulans* (Walker)	
*'Ochlerotatus'* (*'Ochlerotatus'*) *stimulans* (Walker)	
*Ochlerotatus* (subgenus unassigned) *stimulans* (Walker)	
***Aedes* (*Ochlerotatus*) *taeniorhynchus* (Wiedemann)** *	**CVV**, **EEEV**, **EYEV**, **ORIV**, RVFV^+^ ^[^ ^37^ ^]^,
*Ochlerotatus* (*Ochlerotatus*) *taeniorhynchus* (Wiedemann)	**TENV**, **TVTV**, **VEEV**, WNV^[^ ^14^ ^]^, **WYOV**
*'Ochlerotatus'* (*'Ochlerotatus'*) *taeniorhynchus* (Wiedemann)	
*Ochlerotatus* (*Culicelsa*) *taeniorhynchus* (Wiedemann)	
***Aedes* (*Ochlerotatus*) *thelcter* Dyar** *	**VEEV**
*Ochlerotatus* (*Ochlerotatus*) *thelcter* (Dyar)	
*'Ochlerotatus'* (*'Ochlerotatus'*) *thelcter* (Dyar)	
***Aedes* (*Ochlerotatus*) *trivittatus* (Coquillett)** *	EEEV^[^ ^12^ ^]^, **LACV**, **TVTV**, WNV^[^ ^14^ ^]^
*'Ochlerotatus'* (*'Ochlerotatus'*) *trivittatus* (Coquillett)	
*Ochlerotatus* (*Ochlerotatus*) *trivittatus* (Coquillett)	
***Aedes* (*Ochlerotatus*) *vigilax* (Skuse)** *	Non-native^[^ ^30^ ^]^ **, BAMV**, **EHV**, **GGV**, **KOKV**, **RRV**, **SINV**
*Ochlerotatus* (*Ochlerotatus*) *vigilax* (Skuse)	
*'Ochlerotatus'* (*'Ochlerotatus'*) *vigilax* (Skuse)	
*Ochlerotatus* (*Empihals*) *vigilax* (Skuse)	
***Aedes* (*Polyleptiomyia*) *albocephalus* (Theobald)**	WNV^[^ ^45^ ^]^
*Aedes* (*Aedimorphus*) *albocephalus* (Theobald)*	
*'Aedes'* (*'Aedimorphus'*) *albocephalus* (Theobald)	
*Polyleptiomyia albocephala* (Theobald)	
***Aedes* (*Protomacleaya*) *triseriatus* (Say)** *	CVV^[^ ^46^ ^]^, EEEV^[^ ^12^ ^]^, **JCV** ^++^, **LACV**,
*'Ochlerotatus'* (*'Protomacleaya'*) *triseriatus* (Say)	RVFV^+^ ^[^ ^37^ ^]^, WNV^[^ ^14^ ^]^, Potosi^[^ ^46^ ^]^
***Aedes* (*Rampamyia*) *notoscriptus* (Skuse)**	Invasive^[^ ^30^ ^]^
*Aedes* (*Finlaya*) *notoscriptus* (Skuse)*	
*Ochlerotatus* (*Finlaya*) *notoscriptus* (Skuse)	
*'Ochlerotatus'* (*'Finlaya'*) *notoscriptus* (Skuse)	
*Rampamyia notoscripta* (*Skuse*)	
***Aedes* (*Skusea*) *pembaensis* Theobald**	**BUNV**, **TAHV**
*Aedes* (*Skusea*) *pembaensis* Theobald*	
*Skusea pembaensis* (Theobald)	
***Aedes* (*Stegomyia*) *aegypti* (Linnaeus)** *	Invasive^[^ ^30^ ^]^ **, CHIK**, **DENV**, **ORUV**, **VEEV**, **WNV**, **YFV**
*Stegomyia aegypti* (Linnaeus)	
*Stegomyia* (*Stegomyia*) *aegypti* (Linnaeus)	
***Aedes* (*Stegomyia*) *africanus* (Theobald)** *	**BBKV**, **BOUV**, **CHIK**, RVFV^[^ ^47^ ^]^, **YFV**, **ZIKAV**
*Stegomyia africana* Theobald	
*Stegomyia* (subgenus unassigned) *africana* Theobald	
***Aedes* (*Stegomyia*) *albopictus* (Skuse)** *	Invasive^[^ ^30^ ^]^, CHIK^[^ ^48^ ^]^, CVV^[^ ^46^ ^]^,
*Stegomyia albopicta* (Skuse)	**DENV**, EEEV^[^ ^49^ ^]^, LACV^[^ ^46^ ^]^,
*Stegomyia* (subgenus unassigned) *albopicta* (Skuse)	WNV^[^ ^50^ ^]^, YFV^[^ ^51^ ^]^
***Aedes* (*Stegomyia*) *bromeliae* (Theobald)** *	YFV^[^ ^52^ ^]^
*Stegomyia bromeliae* Theobald	
*Stegomyia* (*Mukwaya*) *bromeliae* Theobald	
***Aedes* (*Stegomyia*) *luteocephalus* (Newstead)** *	CHIK^[^ ^23^ ^]^, DEN^[^ ^47^ ^]^, YFV^[^ ^47^ ^]^, **ZIKAV**
*Stegomyia luteocephala* Newstead	
*Stegomyia* (subgenus unassigned) *luteocephala* Newstead	
***Aedes* (*Stegomyia*) *neoafricanus* Cornet, Valade & Dieng** *	**NRIV**
*Stegomyia neoafricana* (Cornet, Valade & Dieng)	
*Stegomyia* (subgenus unassigned) *neoafricana* (Cornet, Valade & Dieng)	
***Aedes* (*Stegomyia*) *opok* Corbet & van Someren** *	**YFV**
*Stegomyia opok* (Corbet & van Someren)	
*Stegomyia* (subgenus unassigned) *opok* (Corbet & van Someren)	
***Aedes* (*Stegomyia*) *polynesiensis* Marks** *	*W*. *bancrofti* ^[^ ^27^ ^,^ ^53^ ^]^
*Stegomyia polynesiensis* (Marks)	
*Stegomyia* (subgenus unassigned) *polynesiensis* (Marks)	
***Aedes* (*Stegomyia*) *scutellaris* (Walker)** *	**DEN** ^[^ ^27^ ^]^, *W*. *bancrofti* ^[^ ^27^ ^]^
*Stegomyia scutellaris* (Walker)	
*Stegomyia* (subgenus unassigned) *scutellaris* (Walker)	
***Aedes* (*Stegomyia*) *simpsoni* (Theobald)** *	** BBKV**, **NRIV** ^+++^, **YFV**
*Stegomyia simpsoni* Theobald	
*Aedes* (*Mukwaya*) *simpsoni* (Theobald)	
***Aedes* (*Tanakius*) *togoi* (Theobald)**	Non-native^[^ ^30^ ^]^, JEV^[^ ^54^ ^]^,
*Aedes* (*Finlaya*) *togoi* (Theobald)*	*Brugia malayi* ^[^ ^54^ ^]^, *W*. *bancrofti* ^[^ ^54^ ^]^,
*Ochlerotatus* (*Finlaya*) *togoi* (Theobald)	*D*. *immitis* ^[^ ^54^ ^]^
*Tanakius togoi* (Theobald)	
***Aedes* (*Zavortinkius*) *longipalpis* (Gruenberg)**	**UGSV**
*Aedes* (*Finlaya*) *longipalpis* (Gruenberg)*	
*Ochlerotatus* (*Finlaya*) *longipalpis* (Gruenberg)	
*Zavortinkius longipalpis* (Gruenberg)	
***Armigeres* (*Armigeres*) *obturbans* (Walker)** *	GETV^[^ ^39^ ^]^, Kadipiro^[^ ^39^ ^]^
***Armigeres* (*Armigeres*) *subalbatus* (Coquillett)** *	JEV^[^ ^55^ ^]^, *W*. *bancrofti* ^[^ ^54^ ^]^
***Haemagogus* (*Haemagogus*) *janthinomys* Dyar** *	YFV^[^ ^56^ ^]^
***Haemagogus* (*Haemagogus*) *mesodentatus* Komp & Kumm** *	YFV^[^ ^55^ ^]^
***Haemagogus* (*Haemagogus*) *spegazzinii* Brethes** *	YFV^[^ ^55^ ^]^
***Haemogogus* (*Conopostegus*) *leucocelanus* (Dyar & Shannon)** *	**WYOV, YFV**
***Psorophora* (*Janthinsoma*) *cyanescens* (Coquillett)** *	**VEEV**
***Psorophora* (*Janthinsoma*) *ferox* (von Humboldt)** *	CVV^[^ ^31^ ^]^, EEEV^[^ ^12^ ^]^, **ORIV**, **SLEV**, WNV^[^ ^14^ ^]^, **WYOV**
***Psorophora* (*Grabhamia*) *columbiae* (Dyar & Knab)** *	WNV^[^ ^14^ ^]^, Potosi^[^ ^22^ ^]^
***Psorophora* (*Grabhamia*) *confinnis* (Lynch Arribálzaga)** *	**CVV**, **TENV**, **VEEV**
***Psorophora* (*Grabhamia*) *discolor* (Coquillett)** *	**VEEV**
***Psorophora* (*Janthinosoma*) *ferox* (von Humboldt)** *	WNV^[^ ^14^ ^]^
***Psorophora* (*Janthinosoma*) *albipes* (Theobald)** *	**WYOV**
***Psorophora* (*Psorophora*) *ciliata* (Fabricius)** *	TENV^[^ ^22^ ^]^, **VEEV**, WNV^[^ ^14^ ^]^
***Psorophora* (*Psorophora*) *cilipes* (Fabricius)** *	**VEEV**
***Psorophora* (*Psorophora*) *howardii* Coquillett** *	WNV^[^ ^14^ ^]^

*Name combination as in Knight & Stone (1977) [1]

^+^ Experimental infection

^++^ Virus detected in eggs

^+++^ Virus detected in males

Virus-species associations listed in bold typeface are those listed on the CDC Arbovirus Catalog (2015) [57]. Virus names and abbreviations: BAMV—Barmah Forest; BATV—Batai; BBKV—Babanki; BOUV—Bouboui; BUNV—Bunyamwera; CHIKV—Chikungunya; CVV—Cache Valley; DENV—Dengue; EEEV—Eastern Equine Encephalitis; EHV—Edge Hill; EYEV—Everglades; GETV—Getah; GGV—Gan Gan; HJV—Highland J; ILHV—Ilheus; JCV—James Canyon; KEDV—Kedougou; LACV—La Crosse; LEBV—Lebombo; MVEV—Murray Valley River; NDOV—Nyando; NRIV—Ngari; ORIV—Oriboca; ORUV—Orungo; PGAV—Pongola; RRV—Ross River; RVFV—Rift Valley Fever; SFV—Semiliki Forest; SINV—Sindbis; SLEV—St Louis Encephalitis; SPOV—Spondweri; TAHV—Tahyna; TENV—Tensaw; TVTV—Trivittatus; UGSV—Uganda S; VEEV—Venezuelan Equine Encephalitis; WNV—West Nile; WSLV—Wesselbron; WYOV—Wyeomyia; YFV—Yellow Fever; ZIKAV—Zika.

## Formal Taxonomic Action

This publication restores the generic structure of the tribe Aedini (Diptera: Culicidae) to its status prior to the year 2000. Specifically, all species in the tribe are placed in the following 10 genera: *Aedes*, *Armigeres*, *Eretmapodites*, *Haemagogus*, *Heizmannia*, *Opifex*, *Psorophora*, *Udaya*, *Verrallina*, and *Zeugnomyia*. Aedine genera created in and after 2000 are placed as subgenera of genus *Aedes*; subgenera created after 2000 are placed in informal species groupings (S1 Appendix, S2 Appendix). The classification proposed here, and further updates to culicid classification, will be reflected in the online taxonomic catalog at www.mosquitocatalog.org.

The 1977 catalog [1] lists nine genera in the tribe Aedini (*Aedes* Meigen, 1818; *Armigeres* Theobald, 1901; *Eretmapodites* Theobald, 1901; *Haemagogus* Williston, 1896; *Heizmannia* Ludlow, 1905; *Opifex* Hutton, 1902; *Psorophora* Robineau-Desvoidy, 1827; *Udaya* Thurman, 1954; and *Zeugnomyia* Leicester, 1908). Of the 51 subgenera in Aedini, 38 subgenera with about 800 species were placed in the genus *Aedes*. An “Aedes Group” was mentioned by Edwards in 1932 [58] in a compendium of mosquito taxonomy that preceded the 1959 and 1977 catalogs. His Aedes Group included all of the above except *Udaya*, which had not yet been described, and *Zeugnomyia*, which was then considered to be in the *Uranotaenia* group. Tribe Aedini with all nine genera was first mentioned by Belkin in 1962 [27] in a two-volume work on mosquitoes of the South Pacific that also discussed mosquito systematics and biogeography in a world-wide context.

Since Knight and Stone’s catalog and supplements, five new aedine subgenera were added (*Isoaedes* [59], *Belkinius* [60], *Albuginosus* [61], *Kenknightia* [62] and *Zavortinkius* [63]). Also during this period three subgenera of *Aedes* were elevated to genus, one in 1999 (*Verrallina* [64]) and two in 2000 (*Ayurakitia* [65] and *Ochlerotatus* [66]). Elevation of *Verrallina* and *Ayurakitia* to genus went largely unnoticed except by taxonomists. In contrast, because *Ochlerotatus* contained some well-known species, e.g. *Ae*. (*Och*.) *sollicitans* (Walker), a salt-marsh mosquito and important nuisance and disease vector (Table 1), there was a good deal of debate as to the merit of elevating it to a genus [67]. The classification that included *Ochlerotatus* as a genus was followed by some workers but ignored by others [68,69]. The recognition of these additional genera and subgenera in Aedini was based on traditional taxonomic judgments of similarities and relationships rather than on quantitative cladistic analysis.

**Fig 1:**
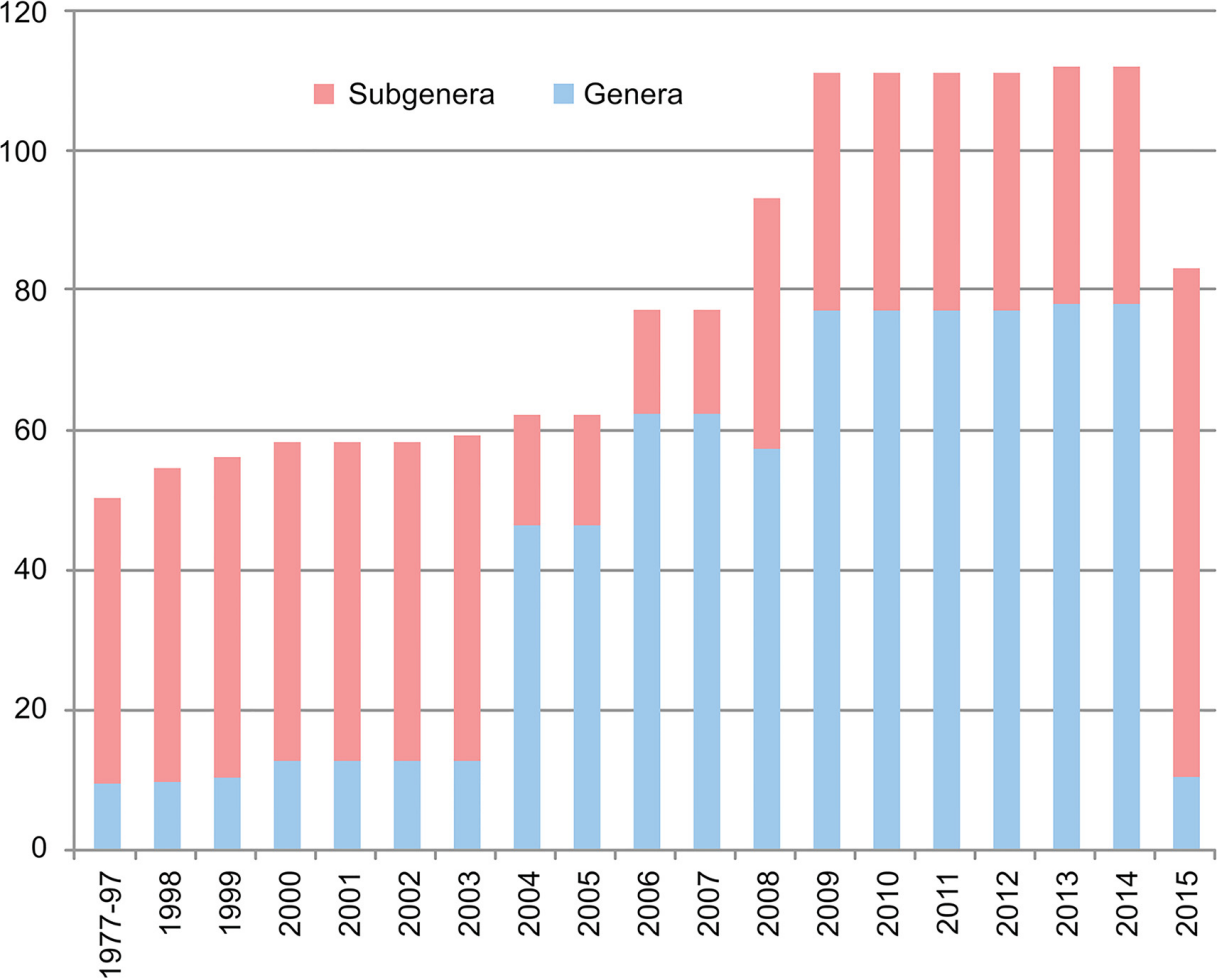
Graph comparing number of aedine mosquito genera and subgenera from 1977 to present.

**Fig 2:**
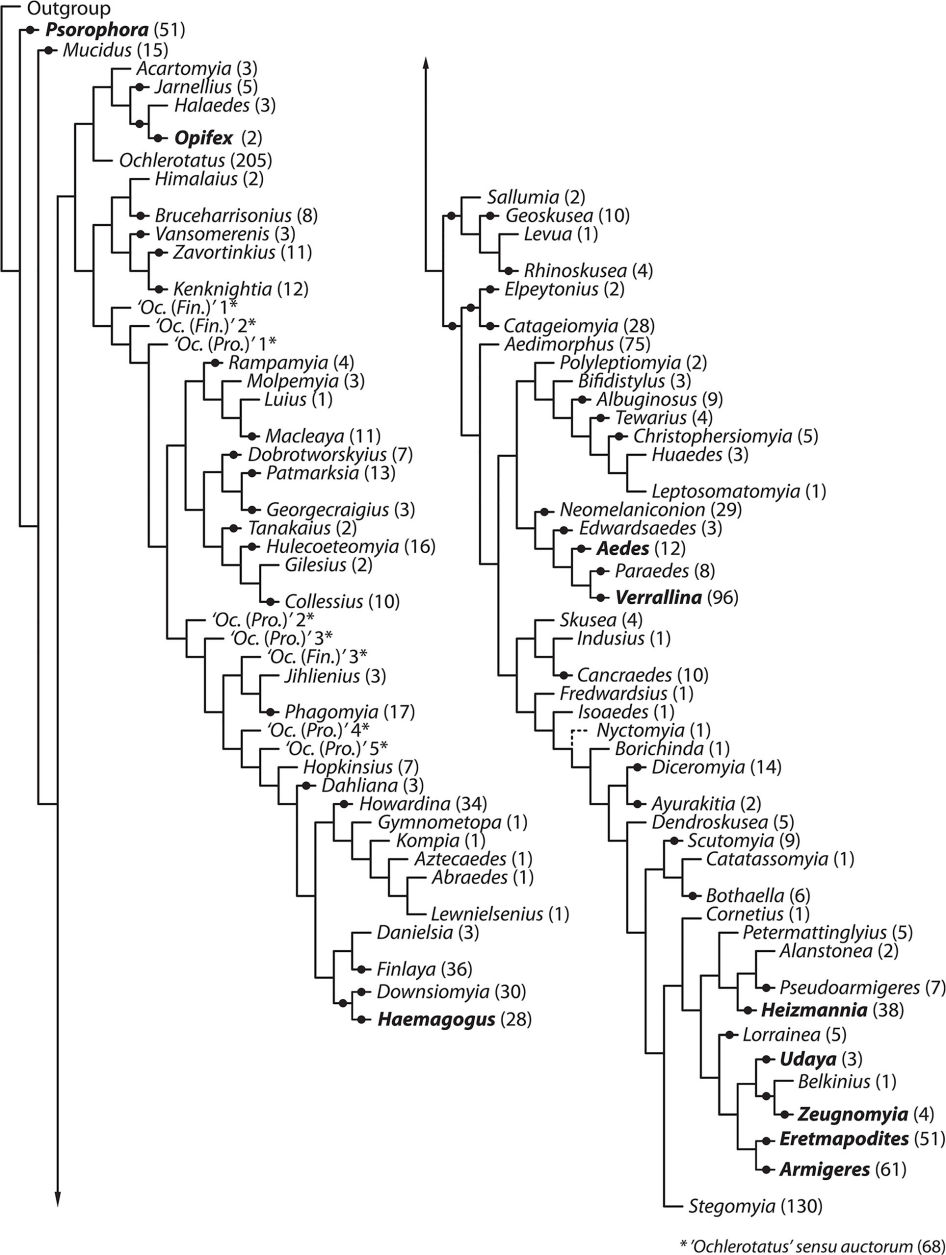
Tree derived from the single most parsimonious cladogram in Reinert, Harbach & Kitching 2009 [73]. Branches with GC≥40 are indicated by dark circles.

In a series of papers published from 2004 through 2009 [70–73], Reinert, Harbach, and Kitching (henceforth RH&K) made many changes to the nomenclature of the tribe Aedini. The RH&K reclassifications [70–73] were based on comprehensive morphological studies of all life stages of the tribe. Their papers report many entirely new morphological observations, especially on the female genitalia. The authors were not able to examine every species in the tribe, but instead chose those species that were available to them and that they considered representative of previously established groups, usually choosing the type species from a genus or subgenus. Based on their results, RH&K identified monophyletic groups and declared new classifications that were further refined in each publication. The series of papers established 74 genera (S1 Fig, Fig 1) from what was formerly genus *Aedes*, essentially tripling the number of genera in the family Culicidae. Most of the decisions on classification, particularly in the 2009 paper [73], were based on quantitative cladistic analyses. These decisions were primarily anchored to an objectively defined measure of "Groups present/Contradicted" (GC) [74]. Those groups were identified by pseudo-replication, or jackknifing, using symmetric resampling in an implied-weights parsimony analysis in the computer program TNT [75]. When a grouping was supported by a GC threshold ≥ 40, it was considered cladistically significant [73]. However, the authors departed from this quantitative scheme by recognizing clades with GC<40 when the “number and nature of the characters that support the branches” were subjectively considered significant (Fig 2). RH&K did not propose diagnostic characters for the new genera, though they did assign many species in Aedini to genus or subgenus based on accepted groupings and their own analyses of characters. Exceptions were: genus *Ochlerotatus*, 138 species unassigned to subgenus; ‘*Ochlerotatus’* (‘*Finlaya’*) *sensu auctorum* (= a name used by subsequent authors in a sense different from that established by the original author [76]), 28 species; ‘*Ochlerotatus’* (‘*Protomacleaya’*) *sensu auctorum*, 39 species; and genus *Stegomyia*, 104 species unassigned to subgenus.

Although phylogenetic studies of DNA sequences should ultimately be helpful in determining taxonomic relationships between groups within Aedini, the few studies to date have not been able to test the validity of the reclassification proposed by RH&K based on morphological characters. DNA barcoding studies have examined 217 aedine species in China [77], India [78], and Afghanistan [79]. Of note, barcoding involves comparison of short sequences of the *COI* mitochondrial gene, producing distance measures that are only useful for delineating and differentiating species-level taxa, not for estimating their relationships. Combining *COI* and *COII* mitochondrial genes from 17 aedine species in western Africa [80] failed to distinguish them as a group from three non-aedine species and also failed to show significant groupings of species. Restriction mapping of rDNA [81] was similarly unsuccessful for a study of 12 culicine species representing six genera. Analysis of the *white* gene [82] successfully distinguished three aedine species from nine others, but the sample size was too small to examine relationships among the aedine species.

The Linnaean system of nomenclature that has served biology since 1758 was formalized in the International Code of Zoological Nomenclature (ICZN), a basic set of rules designed to promote stability and communication [76]. Classifications governed by the ICZN are meant to be “natural,” a term that since the translation of Hennig’s “Phylogenetic Systematics” [83] has been defined as strictly monophyletic, meaning “including all descendants from a common ancestor.” Wiley [84,85], one of the early theorists of phylogenetic systematics, asserted that an annotated Linnaean classification can take more than one form as long as each is logically consistent with a phylogeny, and that “… minimum taxonomic decisions will be made whenever possible to construct a classification or to modify an existing classification.” Wiley and Liebermann [86] also stated that “the ranks of well-known clades will be retained whenever possible.” Although explicitly "tree-based" classification systems have been suggested as alternatives to Linnaean classification (e.g., numerical prefix schemes [87] and the PhyloCode [88]), these alternative schemes require a well-supported phylogeny based on multiple lines of evidence, a condition not yet reached in Aedini as discussed above.

Regardless of the system, some accommodations are necessary for serving the sometimes non-overlapping requirements of taxonomy, information retrieval, and communication [86]. Recently, Vences and colleagues [89] discussed the relationship between nomenclatorial utility and phylogenetic accuracy in an expansion on previous treatments of the subject [84–86]. They acknowledged that taxonomic instability “…can become a serious problem consuming public and private resources…”. To address this and other issues, they proposed several levels of taxon naming criteria (TNCs), the priority criteria being monophyly, clade stability, and phenotypic diagnosability. Accessory criteria included, among others, the medical or economic significance of a taxon. These authors emphasized that “…except for monophyly, the priority TNCs are not proposed as mandatory requirements of a Linnaean taxon but as yardsticks to allow for an informed choice among various clades in a tree…” and warn that “…taxa of unstable monophyly or poor diagnosability reduce the information content and hence the utility of the Linnaean system.”

After reviewing the history of the classification of tribe Aedini and previous morphological and molecular phylogenetic analyses, we reanalyzed RH&K data and performed a critical assessment of their methods and results in the context of Wiley [84,85], Wiley and Lieberman [86]’s, “conventions for annotated Linnaean classifications”, and also Vences et al. [89]’s, “taxon naming criteria”. To preserve information about morphological evolution in mosquitoes, we suggest alternative methods for reflecting RH&K’s findings in a classification that retains the stability necessary for effective information retrieval and communication.

## Materials and Methods

**Fig 3:**
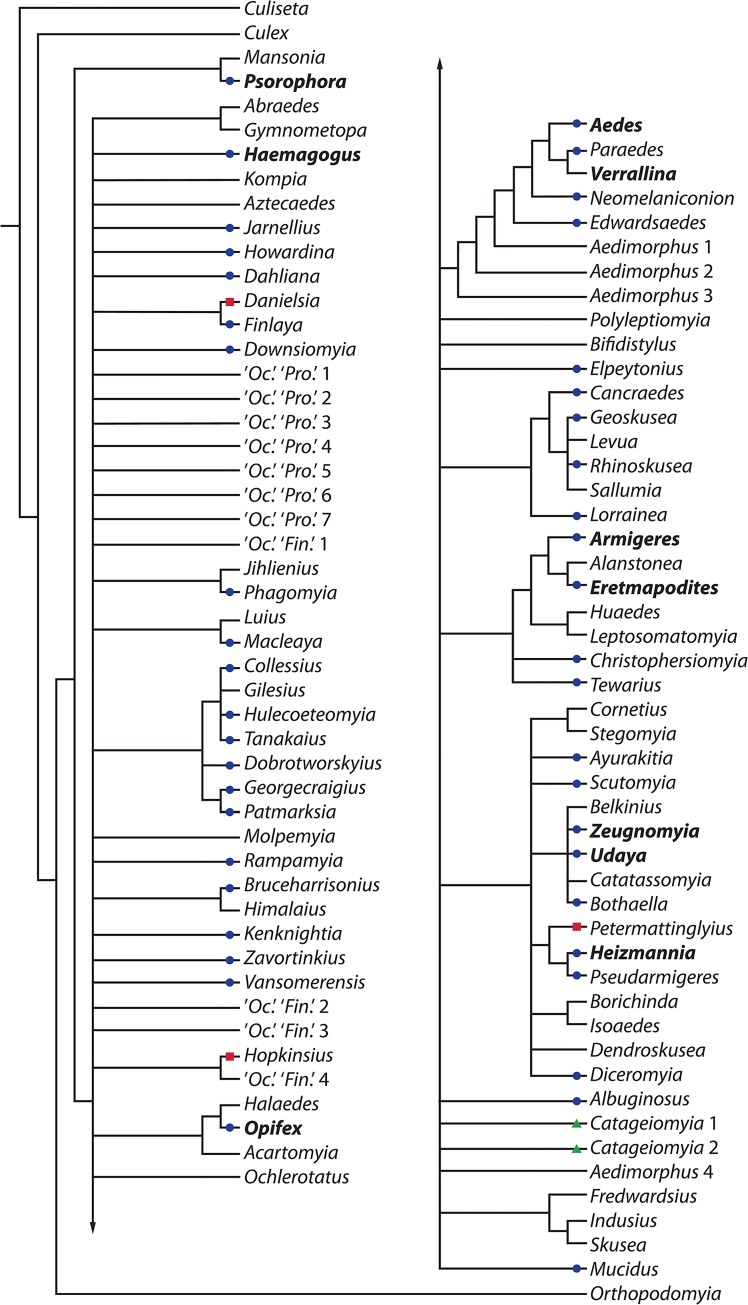
Strict consensus tree derived from the reanalysis of the Aedini morphology data set using unweighted parsimony with 14 ordered characters. Dark circle = nodes supported in [73] and herein; triangle = supported in RH&K [73] but not herein; square = supported herein but not in RH&K [73].

We reanalyzed RH&K’s data in the program TNT [75,90], not for the purpose of producing a classification, but rather to test the robustness of RH&K’s [73] generic groups (Fig 3; S1 Tree, S2 Tree, S3 Tree, S4 Tree) under alternative, but similar, cladistic analyses. We assumed that if proposed relationships among genera are not robust when subjected to closely related analytical methods, they are unlikely to be robust to the introduction of new data. To gauge the stability of RH&K’s [73] generic groupings to standard parsimony-based analytical methods, we reanalyzed their data with two alternative methods: (i) unweighted parsimony jackknife analyses, identifying clades and their GC (Groups present/Contradicted) support values [74] on the resulting jackknife tree, and (ii) unweighted maximum-parsimony analyses, identifying clades occurring on the strict consensus of the resulting most parsimonious trees. For each of these analytical methods, we conducted two sets of multiple analyses, one in which all characters were unordered and one in which 14 characters were ordered, as was the case in a subset of the original analyses [73]. We performed all analyses in TNT v.1.1 [75,90], although in some cases we carried out tree-bisection and reconnection (TBR) branch-swapping on accumulated trees in PAUP* [91].

Jackknife analyses. We conducted unweighted parsimony jackknife analyses consisting of 1,000 pseudoreplicates, each pseudoreplicate consisting of 10 separate tree searches utilizing TBR branch-swapping on a random-taxon-addition Wagner tree and limited to finding 10 most-parsimonious trees. Support for clades was measured with the GC (Groups present/Contradicted) criterion [74] based on the TNT default tree output, which displays a tree consisting of all groups with positive GC values (S1 Tree, S2 Tree). Following RH&K’s criterion, we report nodes with GC≥ 40.

Maximum parsimony analyses. We conducted multiple, progressive, unweighted parsimony analyses in which resulting trees were saved, accumulated trees were subjected to TBR branch-swapping, and duplicate trees were discarded. These analyses employed varying combinations of TNT "new technology" algorithms, including the parsimony ratchet (5 to 100,000 iterations), sectorial searching (RSS and CSS), tree drifting (10–1000 cycles), and tree fusing (5–100 rounds), in which accumulating trees from prior searches were carried over into subsequent searches. Multiple driven searches were also conducted, including (i) 1,000 initial random-taxon-addition sequences with five rounds of ratcheting and three rounds of tree-fusing and requiring that minimum-length trees be found 10 times; and (ii) combined sectorial, ratchet, tree-drifting, and tree-fusion searches requiring the consensus tree to stabilize four times (S3 Tree, S4 Tree).

GC support criterion. GC is calculated as the difference between the frequency with which a given group is retrieved (e.g., in the jackknife replicates) and the frequency with which its most frequent contradictory group is retrieved [74]. Absolute frequencies (the usual method of counting frequencies in jackknife or bootstrap analyses) do not distinguish between a group with a frequency of 60% that is uncontradicted by an alternative grouping (GC = 60) and a group with a frequency of 60% that is contradicted by an alternative grouping that may occur, e.g., 20% of the time, which would produce GC = 40 [74].

Ordered characters. Following RH&K, the 14 ordered characters (out of a total of 336 characters) are all measurement or ratio characters, ordered as suggested by Thiele [92] (characters 13, 14, 17, 20, 62, 67, 72, 84, 107, 138, 161, 273, 280, 318; for TNT characters 12, 13, 16, 19, 61, 66, 71, 83, 106, 137, 160, 272, 279, 317).

## Results

A series of 20 maximum-parsimony searches in which all characters were unordered converged multiple times on the same set of 832 most-parsimonious trees of unweighted length = 6,609 (S4 Tree). A series of 51 maximum-parsimony searches in which 14 characters were ordered produced 4,000 most-parsimonious trees of unweighted length = 6,654 (S3 Tree), but searches continued to find additional trees, so it is likely that not all of the most-parsimonious trees were found. Finding all most-parsimonious trees, however, is not necessary for calculating the strict consensus [93]. The majority of tree islands were likely visited and the strict consensus is likely stable because all 51 searches, including the driven consensus-stabilization searches, employed the parsimony ratchet [94]. At best, any additional most-parsimonious trees would have no effect on the consensus and, at worst, they would further erode its resolution.

Regardless of the specific analysis used (unweighted parsimony or strict consensus, ordered or unordered: see S1 Tree, S2 Tree, S3 Tree, S4 Tree), 48 supported clades, including all 10 pre-2000 Aedine genera, were supported by GC values ≥ 40 (Fig 3). These included 45 of the 46 clades proposed by RH&K, with the exception of the proposed genus *Catageiomyia* that split into two unsupported clades in our analysis (Fig 3). In addition, our analysis supports three clades corresponding to the proposed genera *Danielsia*, *Hopkinsius* and *Petermattinglyius* (Fig 3), which were not supported in the analysis of RH&K (Fig 1 in [73]). Significantly, our analyses split *Aedimorphus* into four distinct clades, none of which were supported by GC values ≥ 40 (Fig 3). Numerous instances of species groupings unsupported according to RH&K’s criteria but nonetheless ultimately elevated to generic status [73] were also not supported in our analyses (S1 Tree, S2 Tree, S3 Tree, S4 Tree, Fig 3). Most importantly, we found minimal support for the phylogenetic relationships among taxa (i.e., very low GC or jackknife support at branches), underscoring the low phylogenetic resolution of the character set used to assign ranks among the taxa. Detailed examples are discussed fully in the next section.

## Discussion

As we would expect, the implied-weight parsimony method used by RH&K [73] resulted in a more resolved phylogeny (Fig 2) than the standard equally weighted parsimony strategy we employed (Fig 3). In particular, and as admitted by the authors [73], the high level of precision of the floating-point arithmetic used to calculate fit in the TNT implementation of the implied-weights method is unlikely to find more than a single optimal cladogram. A single-solution, highly resolved cladogram may serve a valuable purpose in posing bold hypotheses maximally vulnerable to refutation by the addition of new data (i.e., taxa or characters) [95]. However, the potential instability that makes monophyletic groups on optimal trees ideal for the generation of bold hypotheses can make those same groups poor candidates upon which to base stable classifications. Because they recognized this problem, RH&K [73] utilized the support criterion of "Groups present/Contradicted" (GC) ≥ 40, based on jackknifing with symmetric resampling, to judge clades on their implied-weights parsimony tree (Fig 2). Although we applied this same criterion (i.e., GC ≥ 40) in our alternative unweighted parsimony analyses, we found negligible support for the basal branches uniting the terminal taxa, i.e., for higher-level relationships between supraspecific groups (Fig 3). Without a better understanding of the relationships among terminal taxa, application of the principle of equivalent ranking advocated by RH&K is unfeasible.

In light of these results and using Vences et al. [89] as a guide, we make a point-by-point evaluation to assess whether the classification of Aedini as proposed by RH&K [73] meets the taxonomic naming criteria (TNCs) described. Of 11 TNCs discussed by Vences et al. [89] the following seven are applicable to our argument (quotes are from Vences et al. [89] unless otherwise noted).

### TNC 1: Monophyly

“The only strict TNC, […]: All supraspecific taxa must be monophyletic units in the respective species tree (although they might be paraphyletic in any gene tree). Monophyly of a taxon should be assessed by an explicit phylogenetic analysis with adequately dense taxon sampling.”

Monophyly of family Culicidae has been demonstrated [96–98] and Tribe Aedini is monophyletic based on morphological characters [99], egg structure, and physiology [67]. RH&K propose and at least partially demonstrate monophyly for many aedine generic level taxa [73]. However, questions remain about branch support and subjectivity, particularly the use of the GC value as the primary determinant of branch support and the arbitrary use of GC≥40 as the cut-off for significance. Of the 74 terminal clades raised to genera by RH&K [73], 36 are supported according to the GC≥40 criterion. Elevation of the remaining 38 unsupported clades (GC<40) to generic status is not further explained or justified except to reference the “nature of the characters that support the branches.”

RH&K [73] indicate three measures of support on each branch of their most-parsimonious implied-weights tree (Fig 1 in [73]): (i) the absolute (i.e., non-GC) frequency from symmetric resampling (a form of jackknifing that accounts for character weights; see [74] and [90]), (ii) the GC frequency from symmetric resampling, and (iii) the absolute (i.e., non-GC) frequency based on typical (i.e., non-symmetric) jackknife resampling. On some branches, these support measures take on zero (measure i) or negative (measures ii and iii) values, indicating no support or contradictory support for groups that do not appear on the most-parsimonious tree. Except for one case (the clade *Juppius* + *Lepidokenon*), in discussing their choices to elevate groups of species to genus level, RH&K only consider measure ii and ignore the other two measures.

Critically, support (GC≥40) uniting pairs of genera (i.e. putative sister taxa) was limited to two nodes in RH&K’s 2009 analysis [73] (*Downsiomyia*–*Haemagogus* and *Elpeytonius-Catageiomyia*), and more basal support (GC≥40) for relationships between multiple genera occurs only twice (a clade with four genera (*Sallumia*, *Geoskusea*, *Levua*, *Rhinoskusea*, and the entire clade from *Elpeytonius* to *Stegomyia*). However, when GC support values are taken into account, their tree is essentially a polytomy with regard to relationships between species groups (Fig 1 in [73]).

Our simplified RH&K tree (Fig 2) does not show the internal relationships within *Mucidus*, *Ochlerotatus*, *Petermattinglyius*, and *Stegomyia* as proposed by RH&K (Fig 1 in [73]). Genus *Mucidus* had two well-supported subgenera and genus *Ochlerotatus* was divided into 14 subgenera, nine of which were supported (*Pseudoskusea*, *Woodius*, *Culicada*, *Gilesia*, *Ochlerotatus*, *Protoculex*, *Chrysoconops*, *Pholeomyia*, *Rusticoidus*). The branch leading to *Ochlerotatus* subgenera *Juppius* and *Leptokeneon* only has single species scored thus it was not possible to judge their support. Interspersed with the named *Ochlerotatus* subgenera were 124 (of 198) species unassigned to subgenus (Appendix 2 in [73]). Lack of consistency in naming within the *Ochlerotatus* clade was evident. For example, it was unclear why subgenus *Culicelsa* was elevated from synonymy [72] while the genus *Ochlerotatus* species pair *andersoni* and *nivalis*, with similarly poor support, was not given equivalent subgeneric rank [73]. Genus *Petermattinglyius* was split into two subgenera even though only the nominotypical subgenus was supported by GC≥40. Genus *Stegomyia*, containing several medically important species, emerged as two poorly supported clades. Eight well-supported subgenera were created for 30 species of *Stegomyia*, leaving the remaining 100 species unassigned to subgenus. We also noted that genus *Aedimorphus*, was not divided into subgenera by RH&K even though it contains four branches with GC≥40 support [73].

The above hypotheses of monophyly are accepted by us as the best currently available. Given our own results and a reassessment of those presented in RH&K [73], we follow Vences et al. [89], who proposed that subgenera or species group names be used “before the phylogeny is fully resolved” or “when important future changes to classification are to be expected.” Since the monophyly of groups within Aedini is only partially supported, we argue it serves little purpose to subdivide the well-known taxon *Aedes* into many additional generic level groups. It is preferable at this time to preserve the groups as subgenera or informal groups.

### TNC 2: Clade Stability

“Those clades selected for naming as taxa in a phylogeny should be supported by as many different independent data sets and analysis methods as possible, and not strongly contradicted by any of them (strong / significant contradictions require a biological explanation to be put forward).”

This criterion is not met by RH&K since only one data set and one analytical approach (implied-weights parsimony) was considered. Our analyses of the same data set with a closely related method, jackknife unweighted parsimony jackknifing, produced significant support (i.e., by RH&K's criterion of GC≥40) for just over half the terminal clades assumed to be equivalent-rank genera by RH&K. This lack of support for terminal species-group clades, as well as the non-significant support for internal nodes in both RH&K's and our analyses, demonstrates the weakness of the phylogenetic signal in the dataset used by RH&K to infer relationships among taxa upon which they base their taxonomic ranks.

### TNC 3: Phenotypic Diagnosability

“A taxon to which a Linnaean rank is assigned should be diagnosable and identifiable phenotypically. Preference should be on diagnostic characters that are unequivocally synapomorphic, externally visible in as many sexes and life-history stages of the organism as possible, and recognizable also by non-specialists;…..”

For this we also follow Wiley & Liebermann’s [86] description of diagnosis which is: “Diagnoses in revisionary work have a different function than diagnoses used for conveying phylogenetic characters of monophyletic groups.” None of the new or resurrected generic level taxa [70–73] were accompanied by diagnoses or keys. They are characterized instead by lists of homoplasious polythetic characters that are not easily retrievable from the publications [70–73].

### TNC 8: Manageability

“If equally stable and diagnosable clades are alternatively available for naming as taxa, choose those that contain a number of taxa manageable for the human mind. Avoid over splitting and deliberately creating monotypic taxa.”

The avoidance of over splitting applies to diagnosable clades and multiple data sets, which as discussed above fundamentally does not apply. However, we cite TNC 8 because we argue that, based on the available dataset, the classification has been over split and most names are difficult to remember and manage.

### TNC 9: Hall of Fame

“Take particular care with taxa that are of high public interest beyond taxonomy and where communication is thus particularly important. The more prominent a rank the more carefully should any change be applied. Intermediate ranks or unranked taxon names can be used preferentially when Clade Stability and Phenotypic Diagnosability TNCs are not sufficiently met.”

We show 101 high-profile mosquito species in tribe Aedini, 85 formerly in genus *Aedes* (Table 1). Given the importance of the relationship of classification to communication and information retrieval, this alone is reason to use intermediate ranks or informal groups to reflect hypotheses of relationships, at least until the classification is declared stable.

### TNC 10: Nomenclatural Stability

“If equally stable and diagnosable clades are alternatively available for naming as taxa, avoid a classification in which unstable names …. are resurrected from synonymy.” Also, “Minimum taxonomic decisions will be made, whenever possible, to construct a classification or to modify an existing classification.”

Although this TNC is for more than one alternative clade we apply it to the excessive changes proposed for genus *Aedes* between 2000 and 2009. S1 Fig is a summary of currently valid generic-level names with a chronology of changes from 1977–2015. The dominant starting point for the chronology was the 1977 catalog [1]. As a result of Reinert [66] and RH&K [70–73] there have been 118 status and / or rank changes in the original genus *Aedes* (elevation from synonymy, change from subgenus to genus and vice versa, and new names). Several taxonomic changes were proposed and later amended or reversed in subsequent publications e.g., *Kenknightia* (3 changes), *Levua* (4 changes with a reversal), *Pseudoskusea* (3 changes with a reversal), and *Rhinoskusea* (3 changes with a reversal) (S1 Fig). These changes underscore the instability of this proposed classification.

### TNC 11: Community Consensus

“If equally stable and diagnosable clades are alternatively available for naming as taxa, choose a classification which is favored by the majority of taxonomists and, if applicable, other biologists, e.g., because it conserves the traditional content and definition of prominent taxa, …”

We measured community consensus regarding the proposed new classifications of Aedini by RH&K [70–73] by using the number of “hits” in the “Web of Science,” searching “topic” AND “title” on 13 September, 2014. The time reference was from 2004 (elevation of genus *Stegomyia*) to present. We chose a few important species to determine which names researchers were using. Search terms and hits are as follow: “*Aedes aegypti*” (n = 6,300) versus “*Stegomyia aegypti*” (n = 191); “*Aedes albopictus*” (n = 2,042) versus “*Stegomyia albopicta*” (n = 23); “*Aedes japonicus*” (n = 102) versus “*Ochlerotatus japonicus*” (n = 72) and “*Hulecoeteomyia japonica*” (n = 0). The “traditional” use of *Aedes* for this small but important sample of species is obviously preferred by the majority of workers. It is likely that “*Ochlerotatus japonicus”*, a recent introduction to North America [100], received more use because it was adopted in a revision of the influential Darsie & Ward key to North American mosquitoes [101].

RH&K’s work, published from 2004 through 2009 [70–73], caused a dramatic change in the structure of Aedini, as outlined above. The elevation of *Ochlerotatus* to genus level in 2000 [66] removed many familiar species from *Aedes*, which in North America involved most of the floodwater species that formed a functional unit from the standpoint of operational mosquito control. In that sense, *Ochlerotatus* was useful from a functional perspective, even if it meant that morphological keys could not be used to separate them at a generic level (i.e., identifications were performed at a species level and then assigned to either *Ochlerotatus* or *Aedes* retrospectively [67]). The establishment of 74 additional genera by RH&K [70–73] (S1 Fig, Fig 1) removed that functionality. RH&K stated that they were attempting to make divisions within Aedini comparable in taxonomic significance by elevating each monophyletic group to genus rank. They used, but did not define or reference, the “principle of equivalent rank” as one of the bases of this decision. While Hennig [83] introduces and discusses many criteria that should be used in the absolute ranking of higher taxa, such as assigning equal rank to sister taxa, he does not specifically define or highlight a principle of equivalent rank. Application of the “principle of equivalent rank” *sensu* RH&K resulted in many new taxon groups being recognized by those authors as equivalent to the small number of traditionally recognized genera, and therefore raised by them to genus status. The most significant outcomes of this process were the near trebling of the number of total genera and the reduction of the number of species in genus *Aedes* from over 800 to just 12. These proposed changes have caused extensive debate about translating phylogenies into classifications [102–105].

The attempt to align aedine classification with cladistically defined monophyletic groups was probably motivated by a desire to have a natural classification. Such a classification has the potential to make information retrieval easier, in that identifying a particular specimen or population to genus would associate it with biologically significant characteristics shared by species within that genus. Such a classification could be helpful not only by predicting likely characters of unfamiliar species, but also by serving as a powerful tool for educating people about the structure of the larger groups. Unfortunately, in our experience, many of these advantages have not resulted from the new classification because most of the genera cannot be identified except by combinations of character states, i.e., they do not have simple, unreversed diagnostic character states. This problem not only inhibits the assignment of new species to the genera, it also tends to force entomologists to identify specimens to species before assigning them to a genus. In contrast, the traditional genus *Aedes* has morphological and biological characters held in common by its species [67], so that identification of a specimen to *Aedes* is informative. The large number of species in the genus is not in itself a reason to divide it, since it may be the result of an evolutionary radiation of a cohesive genetic architecture that is particularly successful under current conditions (for examples refer to [106]).

Hindrance of communication and information retrieval resulting from the reclassification of Aedini is illustrated by the successive renaming of *Aedes* (*Finlaya*) *japonicus*, a recent invasive species in the US and elsewhere [100]. It was first changed to *Ochlerotatus japonicus* [66] and then *Hulecoeteomyia japonica* [71] (Table 1). The two most globally important arboviral vectors *Aedes* (*Stegomyia*) *aegypti* and *Ae*. (*Stegomyia*) *albopictus* were renamed *Stegomyia aegypti* and *Stegomyia albopicta*, respectively [70] (Table 1). Another example is the invasive Australasian species *Aedes notoscriptus* (Skuse), recently found in California (Kenn Fujioka, Pers. Comm.). During the past decade, this species has been referred to as *Aedes* (*Finlaya*) *notoscriptus* [1], *Ochlerotatus* (*Finlaya*) *notoscriptus* [70], and *Rampamyia notoscripta* [71] (Table 1). Successive name changes during a short period of time are confusing and significantly interfere with critical information retrieval from the literature (Table 1).

In conclusion, we formally restore the generic classification of Aedini to its status prior to the publications of RH&K (S1 Appendix), update the subgenera and informal species groups in genus *Aedes* in accordance with their findings (S1 Appendix, S2 Appendix), and propose updated two and three-letter abbreviations for genera and subgenera, respectively (S3 Appendix). We argue that this action will stabilize the Aedini classification and maximize its usefulness to the operational community, while highlighting the need to strive towards a natural system where group membership will provide insights into ecological, evolutionary, and epidemiological criteria. Basing subgenera and informal species groups on the results of RH&K's morphological research incorporates the progress that they have made in defining clades. In taking these actions we restore the utility of the generic designations within Aedini while safeguarding advances in our understanding of morphological evolution in mosquitoes.

Specifically, we propose the following:

1. Return to a linear classification that retains names as they were commonly used prior to the year 2000. This arbitrarily retains the recent elevation of the morphologically diagnosable genus *Verrallina* (S1 Appendix, S1 Fig).
2. Reduction in rank of all aedine genera created in and after 2000 to subgenera of *Aedes* (S1 Appendix, S1 Fig). Reduction of all subgenera designated by RH&K to informal species-group status (S2 Appendix). This respects the taxonomic conclusions of RH&K [73].
3. Acceptance of a revised list of species (S1 Appendix; abbreviations in S3 Appendix) with appropriate endings, authors, and dates to reflect membership in the genus *Aedes*.

## Supporting Information

S1 Appendix Tribe Aedini generic, subgeneric and species name combinations proposed herein. (PDF)

S2 Appendix Informal group names proposed here for RH&K [73] post-1999 subgenera. (PDF)

S3 Appendix Mosquito generic (two letters) and subgeneric (three letters) abbreviations. Based on: Reinert JF (2009) List of abbreviations for currently valid generic-level taxa in family Culicidae (Diptera). European Mosquito Bulletin 27: 68–76.
(PDF)

S1 Fig Timeline illustration of changes in the classification of Aedini from 1977 to present. Valid generic level names and graphical timeline of nomenclature combination changes in Aedine taxa pre-2000 to date. Genera are indicated in blue, subgenera in green and synonomy (Syn.) in red. Author abbreviations include: D&S = Dyar and Shannon; H&R = Harbach & Rattanarithikul; R-D = Robineau-Desvoidy RH&K = Reinert, Harbach & Kitching; S&P = Shevchenko & Prudkina. Generic abbreviations follow those in Reinert, Harbach & Kitching (2009) and herein as listed in S3 Appendix. *Nyctomyia is a replacement name for the pre-occupied genus name *Nyx* (Harbach et al., Zootaxa, 3683(2), 159–177 (2013).
(PDF)

S1 Tree Unweighted parsimony jackknife analysis of the Aedini morphology dataset with 14 characters ordered. (PDF)

S2 Tree Unweighted parsimony jackknife analysis of the Aedini morphology dataset with 14 characters unordered. (PDF)

S3 Tree Unweighted parsimony analysis of the Aedini morphology dataset identifying clades occurring on the strict consensus of the resulting most parsimonious trees with 14 characters ordered. (PDF)

S4 Tree Unweighted parsimony analysis of the Aedini morphology dataset identifying clades occurring on the strict consensus of the resulting most parsimonious trees with 14 characters unordered. (PDF)
