# Supplementary material for: Making Mosquito Taxonomy Useful: A Stable Classification of Tribe Aedini that Balances Utility with Current Knowledge of Evolutionary Relationships
Source: PLoS One. 2015 Jul 30;10(7):e0133602. doi: 10.1371/journal.pone.0133602 (PMC4520491; doi:10.1371/journal.pone.0133602)
Supplement: S1 Appendix — (PDF) [file pone.0133602.s001.pdf]

Appendix S1. Tribe Aedini generic, subgeneric and species names proposed herein. As appropriate, species endings are written to agree in gender with their respective genera and author parentheses are provided for species described in a genus different than in the current classification.

Genus *Aedes*

Subgenus *Abraedes* Zavortink, 1970

papago Zavortink, 1970

Subgenus *Acartomyia* Theobald, 1903

mariae (Sergent & Sergent, 1903)
phoeniciae Coluzzi & Sabatini, 1968
zammitii (Theobald, 1903)

Subgenus *Aedes* Meigen, 1818

alexandrei Gornostaeva, 2005
cinereus Meigen, 1818
dahuricus Danilov, 1987
dmitryi Gornostaeva, 2005
esoensis Yamada, 1921
geminus Peus, 1970
mubiensis Luh & Shih, 1958
nataliae Gornostaeva, 2005
rossicus Dolbeskin, Gorickaja & Mitrofanova, 1930
sasai Tanaka, Mizusawa & Saugstad, 1975
valeryi Gornostaeva, 2005
yamadai Sasa, Kano & Takahasi, 1950

Subgenus *Aedimorphus* Theobald, 1903

abnormalis (Theobald, 1909)
abnormalis kabwachensis Edwards, 1941
aerarius McIntosh, 1975
albodorsalis Fontenille & Brunhes, 1985
alboscutellatus (Theobald, 1905)
alboventralis (Theobald, 1910)
argenteoscutellatus Carter & Wijesundara, 1948
bambiotai Geoffroy, 1987
bancoi Geoffroy, 1987

bevisi (Edwards, 1915)
caecus (Theobald, 1901)
caliginosus (Graham, 1910)
centropunctatus (Theobald, 1913)
culicinus Edwards, 1922
cumminsii (Theobald, 1903)
cumminsii mediopunctatus (Theobald, 1909)
dalzieli (Theobald, 1910)
davidi Basio, 1971
dentatus (Theobald, 1904)
domesticus (Theobald, 1901)
durbanensis (Theobald, 1903)
durbanensis angolae Ribeiro & Ramos, 1974
ebogoensis Rickenbach & Ferrara, 1965
eritreae Lewis, 1942
fowleri (de Charmoy, 1908)
gibbinsi Edwards, 1935
gouldi Reinert, 1972
grjebinei Hamon, Taufflieb & Maillot, 1957
hirsutus (Theobald, 1901)
hirsutus adenensis Edwards, 1941
holocinctus Edwards, 1941
jamesi (Edwards, 1914)
karooensis Muspratt, 1961
leesoni Edwards, 1932
leptolabis Edwards, 1936
leucarthrius (Speiser, 1909)
longiseta Edwards, 1936
lowisii (Theobald, 1910)
mansouri Qutubuddin, 1959
masoalensis Fontenille & Brunhes, 1985
mathioti Fontenille & Brunhes, 1985
mattinglyi Hamon & Rickenbach, 1954
mediolineatus (Theobald, 1901)
natronius Edwards, 1932
nigricephalus (Theobald, 1901)
nigrostriatus (Barraud, 1927)
oakleyi Stone, 1939
ochraceus (Theobald, 1901)
orbitae Edwards, 1922
ovazzai Hamon & Adam, 1959
pachyurus Edwards, 1936
pallidostriatus (Theobald, 1907)
pampangensis (Ludlow, 1905)

pipersalatus (Giles, 1902)
pubescens Edwards, 1925
punctifemoris (Ludlow, 1921)
quasiunivittatus (Theobald, 1901)
rickenbachi Hamon & Adam, 1959
semlikiensis van Someren, 1950
senyavinensis Knight & Hurlbut, 1949
stenoetrus (Theobald, 1907)
stenoscutus (Edwards, 1912)
subdentatus Edwards, 1936
syntheticus Barraud, 1928
taeniorhynchoides (Christophers, 1911)
tauffliebi Rickenbach & Ferrara, 1965
tricholabis Edwards, 1941
tricholabis bwamba (van Someren, 1950)
trimaculatus (Theobald, 1905)
trukensis Bohart, 1957
vexans (Meigen, 1830)
vexans arabiensis (Patton, 1905)
vexans nipponii (Theobald, 1907)
vexans nocturnus (Theobald, 1903)
wigglesworthi Edwards, 1941

Subgenus *Alanstonea* Mattingly, 1960

brevitibia (Edwards, 1914)
treubi (de Meijere, 1910)

Subgenus *Albuginosus* Reinert, 1987

capensis Edwards, 1924
gilliesi van Someren, 1962
haworthi Edwards, 1923
kapretwae Edwards, 1941
kennethi Muspratt, 1956
marshallii (Theobald, 1901)
ngong van Someren, 1950
stokesi Evans, 1929
teesdalei van Someren, 1954

Subgenus *Ayurakitia* Thurman, 1954

griffithi (Thurman, 1954)
peytoni Reinert, 1972

Subgenus *Aztecaedes* Zavortink, 1972

ramirezi Vargas & Downs, 1950

Subgenus *Belkinius* Reinert, 1982

aurotaeniatus Edwards, 1922

Subgenus *Bifidistylus* Reinert, Harbach & Kitching, 2009

boneti Gil Collado, 1936
boneti kumbae Chwatt, 1948
lamborni Edwards, 1923

Subgenus *Borichinda* Harbach & Rattanarithikul, 2007

cavernicolus (Rattanarithikul & Harbach, 2007)

Subgenus *Bothaella* Reinert, 1973

alongi Galliard & Ngu, 1947
brownscutumus Dong, Zhu & Dong, 1999
eldridgei Reinert, 1973
helenae Reinert, 1973
kleini Reinert, 1973
manhi (Harbach & Cook, 2010)

Subgenus *Bruceharrisonius* Reinert, 2003

alektorovi Stackelberg, 1943
aureostriatus (Doleschall, 1857)
christophersi Edwards, 1922
doonii Wattal, Bhatia & Kalra, 1958
greenii (Theobald, 1903)
hurlbuti Lien, 1967
okinawanus Bohart, 1946
taiwanus Lien, 1968

Subgenus *Cancraedes* Edwards, 1929

cancricomes Edwards, 1922
curtipes Edwards, 1915
indonesiae Mattingly, 1958

kohkutensis Mattingly, 1958
mamoedjoensis Mattingly, 1958
masculinus Mattingly, 1958
palawanicus Mattingly, 1958
penghuensis Lien, 1968
simplex (Theobald, 1903)
thurmanae Mattingly, 1958

Subgenus *Catageiomyia* Theobald, 1903

adami Geoffroy, 1971
argenteopunctatus (Theobald, 1901)
bedfordi Edwards, 1936
chamboni Cornet, 1968
dialloi Hamon & Brengues, 1965
falabreguesi Hamon, 1957
filicis Ingram & de Meillon, 1927
grenieri Hamon, Service, Adam & Taufflieb, 1961
hopkinsi Edwards, 1936
insolens Edwards, 1936
irritans (Theobald, 1901)
lokojoensis Service, 1959
lottei Hamon & Brengues, 1965
microstictus Edwards, 1936
minutus (Theobald, 1901)
mixtus Edwards, 1936
mutilus Edwards, 1936
nyounae Hamon & Adam, 1959
phyllolabis Edwards, 1929
pseudotarsalis van Someren, 1946
punctothoracis (Theobald, 1909)
reali Hamon & Adam, 1959
smithburni van Someren, 1950
tarsalis (Newstead, 1907)
veeniae McIntosh, 1975
wendyae Service, 1959
yangambiensis de Meillon & Lavoipierre, 1944
yvonneae Edwards, 1941

Subgenus *Catatassomyia* Dyar & Shannon, 1925

meronephada (Dyar & Shannon, 1925)

Subgenus *Christophersiomyia* Barraud, 1923

annulirostris (Theobald, 1905)
chionodes Belkin, 1962
gombakensis Mattingly, 1959
ibis Barraud, 1931
thomsoni (Theobald, 1905)

Subgenus *Coetzeemyia* Huang, Mathis & Wilkerson

fryeri (Theobald, 1912)

Subgenus *Collessius* Reinert, Harbach & Kitching, 2006

banksi Edwards, 1922
elsiae (Barraud, 1923)
elsiae vicarius Lien, 1968
hatorii Yamada, 1921
macdougalli Edwards, 1922
macfarlanei (Edwards, 1914)
ningheensis Lei, 1989
pseudotaeniatus (Giles, 1901)
shortti (Barraud, 1923)
tonkinensis Galliard & Ngu, 1947

Subgenus *Cornetius* Huang, 2005

cozi Cornet, 1973

Subgenus *Dahliana* Reinert, Harbach & Kitching, 2006

echinus (Edwards, 1920)
geniculatus (Olivier, 1791)
gilcolladoi (Sánchez-Covisa Villa, Rodríguez Rodríguez & Guillén Llera, 1985)

Subgenus *Danielsia* Theobald, 1904

albotaeniatus (Leicester, 1904)
harperi Knight, 1948
lepchanus (Barraud, 1923)

Subgenus *Dendroskusea* Edwards, 1929

kanarensis Edwards, 1934
micropterus (Giles, 1901)

periskelatus (Giles, 1902)
ramachandrai Reuben, 1967
reginae Edwards, 1922

Subgenus *Diceromyia* Theobald, 1911

adersi (Edwards, 1917)
bananea Wolfs, 1958
cordellieri Huang, 1986
coulangesi Rodhain & Boutonnier, 1983
fascipalpis (Edwards, 1912)
flavicollis Edwards, 1928
furcifer (Edwards, 1913)
grassei Doucet, 1951
madagascarensis van Someren, 1949
mefouensis Ferrara, 1974
sylvaticus Brunhes, 1983
taylori Edwards, 1936
tiptoni Grjebine, 1953
zethus de Meillon & Lavoipierre, 1944

Subgenus *Dobrotworskyius* Reinert, Harbach & Kitching, 2006

alboannulatus (Macquart, 1850)
milsoni (Taylor, 1915)
occidentalis (Skuse, 1889)
rubrithorax (Macquart, 1850)
rupestris Dobrotworsky, 1959
subbasalis Dobrotworsky, 1962
tubbutiensis Dobrotworsky, 1959

Subgenus *Downsiomyia* Vargas, 1950

albolateralis (Theobald, 1908)
alboniveus Barraud, 1934
axitiosus Kulasekera, Knight & Harbach, 1990
dorseyi Knight, 1946
ganapathi Colless, 1958
harinasutai Knight, 1978
idjenensis Brug, 1934
inermis Colless, 1958
lacteus Knight, 1946
laoagensis Knight, 1946
leonis Colless, 1958

litoreus Colless, 1958
mikrokopion Knight & Harrison, 1988
mjobergi (Edwards, 1926)
mohani Knight, 1969
nipponicus LaCasse & Yamaguti, 1948
nippononiveus Sasa & Nakahashi, 1952
nishikawai Tanaka, Mizusawa & Saugstad, 1979
niveoides Barraud, 1934
niveus (Ludlow, 1903)
novoniveus Barraud, 1934
omorii Lien, 1968
pexus Colless, 1958
pseudoniveus (Theobald, 1905)
saperoi Knight, 1946
shehzadae Qutubuddin, 1972
sinensis Chow, 1950
subniveus Edwards, 1922
vanus Colless, 1958
watteni Lien, 1968

Subgenus *Edwardsaedes* Belkin, 1962

bekkui Mogi, 1977
imprimens (Walker, 1861)
pingpaensis Chang, 1965

Subgenus *Elpeytonius* Reinert, Harbach & Kitching, 2009

apicoannulatus (Edwards, 1912)
simulans (Newstead & Carter, 1911)

Subgenus *Finlaya* Theobald, 1903

alocasicola Marks, 1947
ananae Knight & Laffoon, 1946
avistylus Brug, 1939
bougainvillensis Marks, 1947
burnetti Belkin, 1962
croceus Knight & Laffoon, 1946
dobrotworskyi Marks, 1958
fijiensis Marks, 1947
flavipennis Giles, 1904
franclemonti Belkin, 1962
freycinetiae Laird, 1957

fuscipalpis Belkin, 1962
fuscitarsis Belkin, 1962
gahnicola Marks, 1947
gani Bonne-Wepster, 1940
gressitti Bohart, 1957
hollingsheadi Belkin, 1962
horotoi Taylor, 1972
hui Bohart, 1957
josephinae Marks, 1958
knighti Stone & Bohart, 1944
kochi (Dönitz, 1901)
lewelleni Starkey & Webb, 1946
luteus (Ludlow, 1905)
maffii Taylor & Tenorio, 1974
medleri Knight & Laffoon, 1946
neogeorgianus Belkin, 1962
oceanicus Belkin, 1962
poicilius (Theobald, 1903)
samoanus (Grünberg, 1913)
schlosseri Belkin, 1962
solomonis Stone & Bohart, 1944
sorsogonensis Bañez & Jueco, 1966
stonei Knight & Laffoon, 1946
tutuilae Ramalingam & Belkin, 1965
wallacei Edwards, 1926

Subgenus *Fredwardsius* Reinert, 2000

vittatus (Bigot, 1861)

Subgenus *Georgecraigius* Reinert, Harbach & Kitching, 2006

atropalpus (Coquillett, 1902)
epactius Dyar & Knab, 1908
fluviatilis (Lutz, 1904)

Subgenus *Geoskusea* Edwards, 1929

baisasi Knight & Hull, 1951
becki Belkin, 1962
daggyi Stone & Bohart, 1944
fimbripes Edwards, 1924
kabaenensis Brug, 1939
longiforceps Edwards, 1929

lunulatus King & Hoogstraal, 1946
perryi Belkin, 1962
timorensis (Miyagi, Toma & Lien, 2004)
tonsus Edwards, 1924

Subgenus *Gilesius* Reinert, Harbach & Kitching, 2006

alius Lien, 1968
pulchriventer (Giles, 1901)

Subgenus *Gymnometopa* Coquillett, 1905

mediovittatus (Coquillett, 1906)

Subgenus *Halaedes* Belkin, 1962

ashworthi Edwards, 1921
australis (Erichson, 1842)
wardangensis Brust, Ballard, Driver, Hartley, Galway & Curran, 1998

Subgenus *Himalaius* Reinert, Harbach & Kitching, 2006

gilli (Barraud, 1924)
simlensis Edwards, 1922

Subgenus *Hopkinsius* Reinert, Harbach & Kitching, 2008

albocinctus (Barraud, 1924)
barnardi Edwards, 1924
embuensis Edwards, 1930
ingrami Edwards, 1930
nyasae Edwards, 1930
seoulensis Yamada, 1921
wellmanii (Theobald, 1905)

Subgenus *Howardina* Theobald, 1903

albonotatus (Coquillett, 1906)
allotecnon Kumm, Komp & Ruiz, 1940
arborealis Bonne-Wepster & Bonne, 1920
argyrites Dyar & Núñez Tovar, 1927
aureolineatus Berlin, 1969
aurites (Theobald, 1907)
aurivittatus Cerqueira, 1943

bahamensis Berlin, 1969
brevis Berlin, 1969
brevivittatus Berlin, 1969
busckii (Coquillett, 1906)
cozumelensis Díaz Nájera, 1966
ecuadoriensis Berlin, 1969
eleanorae Berlin, 1969
fulvithorax (Lutz, 1904)
grabhami Berlin, 1969
guatemala Berlin, 1969
guerrero Berlin, 1969
inaequalis (Grabham, 1907)
ioliota Dyar & Knab, 1913
leei Berlin, 1969
lorraineae Berlin, 1969
marinkellei Berlin, 1969
martinezi Berlin, 1969
osornoi Berlin, 1969
pseudodominicii Komp, 1936
quadrivittatus (Coquillett, 1902)
septemstriatus Dyar & Knab, 1907
sexlineatus (Theobald, 1901)
spinosus Berlin, 1969
stenei Thompson, 1956
vanemdeni Martini, 1931
walkeri (Theobald, 1901)
whitmorei Dunn, 1918

Subgenus *Huaedes* Huang, 1968

medialis (Brug, 1932)
variepictus (King & Hoogstraal, 1946)
wauensis Huang, 1968

Subgenus *Hulecoeteomyia* Theobald, 1904

chrysolineatus (Theobald, 1907)
formosensis Yamada, 1921
harveyi (Barraud, 1923)
japonicus (Theobald, 1901)
japonicus amamiensis Tanaka, Mizusawa & Saugstad, 1979
japonicus shintienensis Tsai & Lien, 1950
japonicus yaeyamensis Tanaka, Mizusawa & Saugstad, 1979
jugraensis (Leicester, 1908)

koreicus (Edwards, 1917)
nigrorhynchus Brug, 1931
pallirostris Edwards, 1922
reinerti Rattanarithikul & Harrison, 1988
rizali (Banks, 1906)
saxicola Edwards, 1922
sherki Knight, 1948
yunnanensis (Gaschen, 1934)

Subgenus *Indusius* Edwards, 1934

pulverulentus Edwards, 1922

Subgenus *Isoaedes* Reinert, 1979

cavaticus Reinert, 1979

Subgenus *Jarnellius* Reinert, Harbach & Kitching, 2006

deserticola Zavortink, 1969
laguna Arnell & Nielsen, 1972
monticola Belkin & McDonald, 1957
sierrensis (Ludlow, 1905)
varipalpus (Coquillett, 1902)

Subgenus *Jihlienius* Reinert, Harbach & Kitching, 2006

chungi Lien, 1968
gonguoensis Gong & Lu, 1986
unicinctus Edwards, 1922

Subgenus *Kenknightia* Reinert, 1990

dissimilierodes Dong, Zhou & Dong, 2002
dissimilis (Leicester, 1908)
gaffigani Reinert, 1990
harbachi Reinert, 1990
karwari (Barraud, 1924)
lerozeboomi Reinert, 1990
leucomeres (Giles, 1904)
litwakae Reinert, 1990
luzonensis Rozeboom, 1946
paradissimilis Rozeboom, 1946
pecori Reinert, 1990

wilkersoni Reinert, 1990

Subgenus *Kompia* Aitken, 1941

purpureipes Aitken, 1941

Subgenus *Leptosomatomyia* Theobald, 1905

aurimargo Edwards, 1922

Subgenus *Levua* Stone & Bohart, 1944

geoskusea Amos, 1944

Subgenus *Lewnielsenius* Reinert, Harbach & Kitching, 2006

muelleri Dyar, 1920

Subgenus *Lorrainea* Belkin, 1962

amesii (Ludlow, 1903)
celebicus Mattingly, 1959
dasyorrhus King & Hoogstraal, 1946
fumidus Edwards, 1928
lamelliferus Bohart & Ingram, 1946

Subgenus *Luius* Reinert, Harbach & Kitching, 2008

fengi Edwards, 1935

Subgenus *Macleaya* Theobald, 1903

calabyi Marks, 1963
elchoensis Taylor, 1929
humeralis Edwards, 1922
littlechildi Taylor, 1933
macmillani Marks, 1964
moloiensis Taylor, 1929
spinosipes Edwards, 1922
stoneorum Marks, 1977
tremulus (Theobald, 1903)
tulliae (Taylor, 1929)
wattensis Taylor, 1929

Subgenus *Molpemyia* Theobald, 1910

auridorsum Edwards, 1922
pecuniosus Edwards, 1922
purpureus (Theobald, 1910)

Subgenus *Mucidus* Theobald, 1901

alternans (Westwood, 1835)
aurantius (Theobald, 1907)
aurantius chrysogaster (Taylor, 1927)
ferinus Knight, 1947
grahamii (Theobald, 1909)
laniger (Wiedemann, 1820)
lucianus Muspratt, 1959
mucidus (Karsch, 1887)
nigerrimus (Theobald, 1913)
nigrescens (Edwards, 1929)
quadripunctis (Ludlow, 1910)
quasiferinus Mattingly, 1961
scatophagoides (Theobald, 1901)
sudanensis (Theobald, 1908)
tonkingi Gebert, 1948

Subgenus *Neomelaniconion* Newstead, 1907

albicosta (Edwards, 1913)
albiradius (le Goff, Boussès & Brunhes, 2007)
albothorax (Theobald, 1907)
aurovenatus Worth, 1960
belleci (le Goff, Boussès & Brunhes, 2007)
bequaerti Wolfs, 1947
bergerardi Pajot & Geoffroy, 1971
bolense Edwards, 1936
carteri Edwards, 1936
circumluteolum (Theobald, 1908)
crassiforceps Edwards, 1927
ellinorae Edwards, 1941
flavimargo Edwards, 1941
fontenillei (le Goff, Boussès & Brunhes, 2007)
fuscinervis (Edwards, 1914)
jamoti Hamon & Rickenbach, 1954
lineatopennis (Ludlow, 1905)
lineatopennis aureum (Gutsevich, 1955)

luridus McIntosh, 1971
luteolateralis (Theobald, 1901)
mcintoshi Huang, 1985
monotrichus Edwards, 1936
nigropterum (le Goff, Boussès & Brunhes, 2007)
palpale Newstead, 1907
pogonurus Edwards, 1936
punctocostalis (Theobald, 1909)
sylvaticum (le Goff, Boussès & Brunhes, 2007)
taeniarostris (Theobald, 1909)
unidentatum McIntosh, 1971

Subgenus *Nyctomyia* Harbach, 2013

pholeocolus (Linton & Harbach, 2013)
biunguiculatus (Harbach, 2014)

Subgenus *Ochlerotatus* Lynch Arribalzaga, 1891

aboriginis Dyar, 1917
abserratus (Felt & Young, 1904)
aculeatus (Theobald, 1903)
aenigmaticus Cerqueira & Costa, 1946
akkeshiensis Tanaka, 1998
albescens Edwards, 1921
albifasciatus (Macquart, 1838)
albineus Séguy, 1923
aloponotum Dyar, 1917
ambreensis Rodhain & Boutonnier, 1983
andersoni Edwards, 1926
angustivittatus Dyar & Knab, 1907
annulipes (Meigen, 1830)
antipodeus (Edwards, 1920)
atactavittatus Arnell, 1976
atlanticus Dyar & Knab, 1906
auratus Grabham, 1906
aurifer (Coquillett, 1903)
bancroftianus Edwards, 1921
behningi Martini, 1926
bejaranoi Martinez, Carcavallo & Prosen, 1960
berlandi Séguy, 1921
bicristatus Thurman & Winkler, 1950
bimaculatus (Coquillett, 1902)
biskraensis Brunhes, 1999

bogotanus (Arnell, 1976)
breedensis Muspratt, 1953
burjaticus (Kuchartshuk, 1973)
burpengaryensis (Theobald, 1905)
caballus (Theobald, 1912)
cacozelus Marks, 1963
calcariae Marks, 1957
calumnior Belkin, Heinemann & Page, 1970
campestris Dyar & Knab, 1907
camptorhynchus (Thomson, 1869)
canadensis (Theobald, 1901)
canadensis mathesoni Middlekauff
cantans (Meigen, 1818)
cantator (Coquillett, 1903)
caspius (Pallas, 1771)
caspius hargreavesi Edwards, 1920
caspius meirae Ribeiro, Ramos, Capela & Pires, 1980
cataphylla Dyar, 1916
chelli (Edwards, 1915)
churchillensis Ellis & Brust, 1973
clelandi (Taylor, 1914)
clivis Lanzaro & Eldridge, 1992
coluzzii Rioux, Guilvard & Pasteur, 1998
comitatus Arnell, 1976
communis (de Geer, 1776)
condolescens Dyar & Knab, 1907
continentalis Dobrotworsky, 1960
crinifer (Theobald, 1903)
culiciformis (Theobald, 1905)
cunabulanus Edwards, 1924
cyprioides Danilov & Stupin, 1982
cyprius Ludlow, 1920
dahlae (Nielsen, 2009)
decticus Howard, Dyar & Knab, 1917
deficiens Arnell, 1976
detritus Haliday, 1833
diantaeus Howard, Dyar & Knab, 1913
dorsalis (Meigen, 1830)
dufouri Hamon, 1953
duplex Martini, 1926
dupreei (Coquillett, 1904)
dzeta Séguy, 1924
edgari Stone & Rosen, 1952
eidsvoldensis Mackerras, 1927

eucephalaeus Dyar, 1918
euedes Howard, Dyar & Knab, 1913
euiris Dyar, 1922
euplocamus Dyar & Knab, 1906
excrucians (Walker, 1856)
explorator Marks, 1964
fitchii (Felt & Young, 1904)
flavescens (Müller, 1764)
flavifrons (Skuse, 1889)
fulvus (Wiedemann, 1828)
fulvus pallens Ross, 1943
grossbecki Dyar & Knab, 1906
gutzevichi Dubitsky & Deshevykh, 1978
hakusanensis Yamaguti & Tamaboko, 1954
harrisoni Muspratt, 1953
hastatus Dyar, 1922
hesperonotius Marks, 1959
hexodontus Dyar, 1916
hodgkini Marks, 1959
hokkaidensis Tanaka, Mizusawa & Saugstad, 1979
hungaricus Mihalyi, 1955
imperfectus Dobrotworsky, 1962
impiger (Walker, 1848)
impiger daisetsuzanus Tanaka, Mizusawa & Saugstad, 1979
implicatus Vockeroth, 1954
incomptus Arnell, 1976
increpitus Dyar, 1916
inexpectatus Bonne-Wepster, 1948
infirmatus Dyar & Knab, 1906
intermedius Danilov & Gornostaeva, 1987
intrudens Dyar, 1919
jacobinae Serafim & Davis, 1933
jorgi Carpintero & Leguizamón, 2000
juppi McIntosh, 1973
kasachstanicus Gutsevich, 1962
krymmontanus Alekseev, 1989
lasaensis Meng, 1962
lasaensis gyirongensis Ma, 1982
lepidonotus Edwards, 1920
lepidus Cerqueira & Paraense, 1945
leucomelas (Meigen, 1804)
linesi Marks, 1964
longifilamentus Su & Zhang, 1988
luteifemur Edwards, 1926

macintoshi Marks, 1959
martineti Senevet, 1937
mcdonaldi Belkin, 1962
melanimon Dyar, 1924
meprai Martinez & Prosen, 1953
mercurator Dyar, 1920
milleri Dyar, 1922
mitchellae (Dyar, 1905)
montchadskyi Dubitsky, 1968
multiplex (Theobald, 1903)
nevadensis Chapman & Barr, 1964
nigrinus (Eckstein, 1918)
nigripes (Zetterstedt, 1838)
nigrithorax (Macquart, 1847)
nigrocanus Martini, 1927
nigromaculis (Ludlow, 1906)
niphadopsis Dyar & Knab, 1918
nivalis Edwards, 1926
normanensis (Taylor, 1915)
nubilus Theobald, 1903
obturbator Dyar & Knab, 1907
oligopistus Dyar, 1918
patersoni Shannon & del Ponte, 1928
pectinatus Arnell, 1976
pennai Antunes & Lane, 1938
perkinsi Marks, 1949
pertinax Grabham, 1906
phaecasiatus Marks, 1964
phaeonotus Arnell, 1976
pionips Dyar, 1919
postspiraculosus Dobrotworsky, 1961
procax (Skuse, 1889)
provocans (Walker, 1848)
pseudonormanensis Marks, 1949
pulcritarsis (Rondani, 1872)
pulcritarsis asiaticus Edwards, 1926
pullatus (Coquillett, 1904)
punctodes Dyar, 1922
punctor (Kirby, 1837)
purpuraceus Brug, 1932
purpureifemur Marks, 1959
purpuriventris Edwards, 1926
quasirusticus Torres Cañamares, 1951
ratcliffei Marks, 1959

raymondi del Ponte, Castro & García, 1951
refiki Medschid, 1928
rempeli Vockeroth, 1954
rhyacophilus da Costa Lima, 1933
riparioides Su & Zhang, 1987
riparius Dyar & Knab, 1907
rusticus (Rossi, 1790)
rusticus subtrichurus Martini, 1927
rusticus trichurus (Dyar, 1904)
sagax (Skuse, 1889)
sapiens Marks, 1964
scapularis (Rondani, 1848)
schizopinax Dyar, 1929
schtakelbergi Shingarev, 1928
scutellalbum Boshell-Manrique, 1939
sedaensis Lei, 1989
sergievi Danilov, Markovich & Proskuryakova, 1978
serratus (Theobald, 1901)
shannoni Vargas & Downs, 1950
silvestris (Dobrotworsky, 1961)
simanini Gutsevich, 1966
sinkiangensis Hsiao, 1977
sollicitans (Walker, 1856)
spencerii (Theobald, 1901)
spencerii idahoensis (Theobald, 1903)
spilotus Marks, 1963
squamiger (Coquillett, 1902)
sticticus (Meigen, 1838)
stigmaticus Edwards, 1922
stimulans (Walker, 1848)
stramineus Dubitzky, 1970
stricklandi (Edwards, 1912)
subalbirostris Klein & Marks, 1960
subdiversus Martini, 1926
surcoufi (Theobald, 1912)
synchytus Arnell, 1976
taeniorhynchus (Wiedemann, 1821)
tahoensis Dyar, 1916
thelcter Dyar, 1918
theobaldi (Taylor, 1914)
thibaulti Dyar & Knab, 1910
tormentor Dyar & Knab, 1906
tortilis (Theobald, 1903)
trivittatus (Coquillett, 1902)

turneri Marks, 1963
upatensis Anduze & Hecht, 1943
ventrovittis Dyar, 1916
vigilax (Skuse, 1889)
vigilax ludlowae (R. Blanchard)
vigilax vansomerenae Mattingly
vittiger (Skuse, 1889)
washinoi Lanzaro & Eldridge, 1992

Subgenus *Paraedes* Edwards, 1934

barraudi (Edwards, 1934)
bonneae Mattingly, 1958
chrysoscuta (Theobald, 1910)
collessi Mattingly, 1958
menoni Mattingly, 1958
ostentatio (Leicester, 1908)
pagei (Ludlow, 1911)
thailandensis Reinert, 1976

Subgenus *Patmarksia* Reinert, Harbach & Kitching, 2006

anggiensis Bonne-Wepster, 1937
argenteitarsis Brug, 1932
argyronotum Belkin, 1962
buxtoni Belkin, 1962
clintoni Taylor, 1946
derooki Brug, 1932
dobodurus King & Hoogstraal, 1946
hollandius King & Hoogstraal, 1946
mackerrasi Taylor, 1927
novalbitarsis King & Hoogstraal, 1946
palmarum Edwards, 1924
papuensis (Taylor, 1914)
subalbitarsis King & Hoogstraal, 1946

Subgenus *Petermattinglyius* Reinert, Harbach & Kitching, 2009

franciscoi Mattingly, 1959
iyengari Edwards, 1923
punctipes Edwards, 1921
scanloni Reinert, 1970
whartoni Mattingly, 1965

Subgenus *Phagomyia* Theobald, 1905

assamensis (Theobald, 1908)
cacharanus (Barraud, 1923)
cogilli Edwards, 1922
deccanus (Barraud, 1923)
feegradei Barraud, 1934
gubernatoris (Giles, 1901)
gubernatoris kotiensis Barraud, 1934
inquinatus Edwards, 1922
iwi Marks, 1955
khazani Edwards, 1922
kiangsiensis Tung, 1955
lophoventralis (Theobald, 1910)
melanopterus (Giles, 1904)
plumiferus King & Hoogstraal, 1946
prominens (Barraud, 1923)
stevensoni (Barraud, 1923)
watasei Yamada, 1921

Subgenus *Polyleptiomyia* Theobald, 1905

albocephalus (Theobald, 1903)
gandarai da Cunha Ramos, Capela & Ribeiro, 1995

Subgenus *Pseudoarmigeres* Stone & Knight, 1956

albomarginatus (Newstead, 1907)
argenteoventralis (Theobald, 1909)
argenteoventralis dunni (Evans, 1928)
kummi Edwards, 1930
michaelikati van Someren, 1946
michaelikati gurneri van Someren, 1946
natalensis Edwards, 1930

Subgenus *Rampamyia* Reinert, Harbach & Kitching, 2006

albilabris Edwards, 1925
notoscriptus (Skuse, 1889)
notoscriptus montanus Brug, 1939
quinquelineatus Edwards, 1922

Subgenus *Rhinoskusea* Edwards, 1929

longirostris (Leicester, 1908)
pillaii Mattingly, 1958
portonovoensis Tewari & Hiriyan, 1992
wardi Reinert, 1976

Subgenus *Sallumia* Reinert, Harbach & Kitching, 2008

hortator Dyar & Knab, 1907
perventor Cerqueira & Costa, 1946

Subgenus *Scutomyia* Theobald, 1904

albolineatus (Theobald, 1904)
arboricola Knight & Rozeboom, 1946
bambusicola Knight & Rozeboom, 1946
boharti Knight & Rozeboom, 1946
hoogstraali Knight & Rozeboom, 1946
impatibilis (Walker, 1860)
laffooni Knight & Rozeboom, 1946
platylepidus Knight & Hull, 1951
pseudalbolineatus Brug, 1939

Subgenus *Skusea* Theobald, 1903

cartroni (Ventrillon, 1906)
lambrechti van Someren, 1971
moucheti Ravaonjanahary & Brunhes, 1977
pembaensis Theobald, 1901

Subgenus *Stegomyia* Theobald, 1901

aegypti (Linnaeus, 1762)
aegypti formosus (Walker, 1848)
africanus (Theobald, 1901)
agrihanensis Bohart, 1957
albopictus (Skuse, 1895)
alcasidi Huang, 1972
alorensis Bonne-Wepster & Brug, 1932
amaltheus de Meillon & Lavoipierre, 1944
andrewsi Edwards, 1926
angustus Edwards, 1935
annandalei (Theobald, 1910)
aobae Belkin, 1962
apicoargenteus (Theobald, 1909)

bambusae Edwards, 1935
blacklocki Evans, 1925
bromeliae (Theobald, 1911)
burnsi Basio & Reisen, 1971
calceatus Edwards, 1924
chaussieri Edwards, 1923
chemulpoensis Yamada, 1921
contiguus Edwards, 1936
cooki Belkin, 1962
corneti Huang, 1986
craggi (Barraud, 1923)
cretinus Edwards, 1921
daitensis Miyagi & Toma, 1981
deboeri Edwards, 1926
demeilloni Edwards, 1936
denderensis Wolfs, 1949
dendrophilus Edwards, 1921
desmotes (Giles, 1904)
dybasi Bohart, 1957
ealaensis Huang, 2004
edwardsi (Barraud, 1923)
ethiopiensis Huang, 2004
flavopictus Yamada, 1921
flavopictus downsi Bohart & Ingram, 1946
flavopictus miyarai Tanaka, Mizusawa & Saugstad, 1979
fraseri (Edwards, 1912)
futunae Belkin, 1962
galloisi Yamada, 1921
galloisiodes Liu & Lu, 1984
gandaensis Huang, 2004
gardnerii (Ludlow, 1905)
gardnerii imitator (Leicester, 1908)
grantii (Theobald, 1901)
guamensis Farner & Bohart, 1944
gurneyi Stone & Bohart, 1944
hakanssoni Knight & Hurlbut, 1949
hansfordi Huang, 1997
hebrideus Edwards, 1926
heischi van Someren, 1951
hensilli Farner, 1945
hogsbackensis Huang, 2004
hoguei Belkin, 1962
horrescens Edwards, 1935
josiahae Huang, 1988

katherinensis Woodhill, 1949
keniensis van Someren, 1946
kenyae van Someren, 1946
kesseli Huang & Hitchcock, 1980
kivuensis Edwards, 1941
krombeini Huang, 1975
langata van Someren, 1946
ledgeri Huang, 1981
lilii (Theobald, 1910)
luteocephalus (Newstead, 1907)
maehleri Bohart, 1957
malayensis Colless, 1962
malikuli Huang, 1973
marshallensis Stone & Bohart, 1944
mascarensis MacGregor, 1924
masseyi Edwards, 1923
mattinglyorum Huang, 1994
maxgermaini Huang, 1990
mediopunctatus (Theobald, 1905)
mediopunctatus sureilensis Barraud, 1934
metallicus (Edwards, 1912)
mickevichae Huang, 1988
mpusiensis Huang, 2004
muroafcete Huang, 1997
neoafricanus Cornet, Valade & Dieng, 1978
neogalloisi Chen & Chen, 2000
neopandani Bohart, 1957
njombiensis Huang, 1997
novalbopictus Barraud, 1931
opok Corbet & van Someren, 1962
palauensis Bohart, 1957
pandani Stone, 1939
patriciae Mattingly, 1954
paullusi Stone & Farner, 1945
pernotatus Farner & Bohart, 1944
perplexus (Leicester, 1908)
polynesiensis Marks, 1951
poweri (Theobald, 1905)
pseudalbopictus (Borel, 1928)
pseudoafricanus Chwatt, 1949
pseudonigeria (Theobald, 1910)
pseudoscutellaris (Theobald, 1910)
quasiscutellaris Farner & Bohart, 1944
rhungkiangensis Chang & Chang, 1974

riversi Bohart & Ingram, 1946
robinsoni Belkin, 1962
rotanus Bohart & Ingram, 1946
rotumae Belkin, 1962
ruwenzori Haddow & van Someren, 1950
saimedres Huang, 1988
saipanensis Stone, 1945
sampi Huang, 2004
schwetzi Edwards, 1926
scutellaris (Walker, 1858)
scutoscriptus Bohart & Ingram, 1946
seampi Huang, 1974
seatoi Huang, 1969
segermanae Huang, 1997
sibiricus Danilov & Filippova, 1978
simpsoni (Theobald, 1905)
soleatus Edwards, 1924
strelitziae Muspratt, 1950
subalbopictus Barraud, 1931
subargenteus Edwards, 1925
tabu Ramalingam & Belkin, 1965
tongae Edwards, 1926
tulagiensis Edwards, 1926
unilineatus (Theobald, 1906)
upolensis Marks, 1957
usambara Mattingly, 1953
varuae Belkin, 1962
vinsoni Mattingly, 1953
w-albus (Theobald, 1905)
wadai Tanaka, Mizusawa & Saugstad, 1979
woodi Edwards, 1922

Subgenus *Tanakaius* Reinert, Harbach & Kitching, 2004

savoryi Bohart, 1957
togoi (Theobald, 1907)

Subgenus *Tewarius* Reinert, 2006

agastyai Tewari & Hiriyan, 1992
nummatus Edwards, 1923
pseudonummatus Reinert, 1973
reubenae Tewari & Hiriyan, 1992

Subgenus *Vansomerenis* Reinert, Harbach & Kitching, 2006

luteostriatus Robinson, 1950
pulchrithorax Edwards, 1939
hancocki van Someren, 1962

Subgenus *Zavortinkius* Reinert, 1999

brunhesi Reinert, 1999
brygooi Brunhes, 1971
fulgens (Edwards, 1917)
geoffroyi Reinert, 1999
huangae Reinert, 1999
interruptus Reinert, 1999
longipalpis (Grünberg, 1905)
monetus Edwards, 1935
mzooi van Someren, 1962
phillipi van Someren, 1949
pollinctor (Graham, 1910)

Subgenus uncertain

aitkeni Schick, 1970
alboapicus Schick, 1970
alticolaa Bonne-Wepster, 1948
amabilis Schick, 1970
argyrothorax Bonne-Wepster & Bonne, 1920
auronitens Edwards, 1922
australiensis (Theobald, 1910)
berlini Schick, 1970
bertrami Schick, 1970
biocellatus (Taylor, 1915)
braziliensis Gordon & Evans, 1922
brelandi Zavortink, 1972
britteni Marks & Hodgkin, 1958
buenaventura Schick, 1970
burgeri Zavortink, 1972
campana Schick, 1970
candidoscutellum Marks, 1947
casali Schick, 1970
chionotum Zavortink, 1972
crossi Lien, 1967
daliensis (Taylor, 1916)
daryi Schick, 1970

diazi Schick, 1970
eatoni (Edwards, 1916)
gabriel Schick, 1970
galindoi Schick, 1970
gracilelineatus Bonne-Wepster, 1937
hendersoni Cockerell, 1918
heteropus Dyar, 1921
homoeopus Dyar, 1922
idanus Schick, 1970
impostor Schick, 1970
insolitus (Coquillett, 1906)
keefei King & Hoogstraal, 1946
knabi (Coquillett, 1906)
kompi Vargas & Downs, 1950
koreicoides Sasa, Kano & Hayashi, 1950
lauriei (Carter, 1920)
mallochi Taylor, 1944
metoecopus Dyar, 1925
monocellatus Marks, 1948
niveoscutum Zavortink, 1972
oreophilus (Edwards, 1916)
peipingensis Feng, 1938
plagosus Marks, 1959
podographicus Dyar & Knab, 1906
quasirubithorax (Theobald, 1910)
roai Belkin, 1962
rubiginosus Belkin, 1962
sandrae Zavortink, 1972
schicki Zavortink, 1972
schroederi Schick, 1970
sintoni (Barraud, 1924)
stanleyi Peters, 1963
subauridorsum Marks, 1948
suffusus Edwards, 1922
sumidero Schick, 1970
tehuantepec Schick, 1970
terrens (Walker, 1856)
thorntoni Dyar & Knab, 1907
toxopeusi Bonne-Wepster, 1948
triseriatus (Say, 1823)
tsiliensis King & Hoogstraal, 1946
vargasi Schick, 1970
versicolor (Barraud, 1924)
wasselli Marks, 1947

zavortinki Schick, 1970
zoosophus Dyar & Knab, 1918

Not placed by Sathe & Girhe, 2002

kolhapuriensis Sathe & Girhe, 2002
panchgangee Sathe & Girhe, 2002
sangitee Sathe & Girhe, 2002
sangiti Girhe & Sathe, 2001

Genus *Armigeres*

Armigeres Theobald, 1901

alkatirii Toma, Miyagi & Syafruddin, 1995
apoensis Bohart & Farner, 1944
aureolineatus (Leicester, 1908)
azurini Basio, 1971
baisasi Stone & Thurman, 1958
bhayungi Thurman & Thurman, 1958
breinli (Taylor, 1914)
candelabrifer Brug, 1939
confusus Edwards, 1915
conjungens Edwards, 1914
denbesteni Brug, 1925
durhami (Edwards, 1917)
ejercitoi Baisas, 1935
fimbriatus Edwards, 1930
foliatus Brug, 1931
giveni Edwards, 1926
hybridus Edwards, 1914
joloensis (Ludlow, 1904)
jugraensis (Leicester, 1908)
kesseli Ramalingam, 1987
kinabaluensis Ramalingam, 1972
kuchingensis Edwards, 1915
lacuum Edwards, 1922
laoensis Toma & Miyagi, 2003
maiae (Edwards, 1917)
malayi (Theobald, 1901)
manalangi Baisas, 1935
maximus Edwards, 1922
milnensis Lee, 1944
moultoni Edwards, 1914

obturbans (Walker, 1859)
pallithorax Dong, Zhou & Dong, 2004
papuensis Peters, 1963
setifer Delfinado, 1966
seticoxitus Luh & Li, 1981
subalbatus (Coquillett, 1898)
subalbatus *chrysocorporis* Hsieh & Liao, 1956
theobaldi Barraud, 1934
yunnanensis Dong, Zhou & Dong, 1995

Leicesteria Theobald, 1904

annulipalpis (Theobald, 1910)
annulitarsis (Leicester, 1908)
balteatus Macdonald, 1960
cingulatus (Leicester, 1908)
dentatus Barraud, 1927
digitatus (Edwards, 1914)
dolichocephalus (Leicester, 1908)
flavus (Leicester, 1908)
inchoatus Barraud, 1927
lepidocoxitus Dong, Zhou & Dong, 1995
longipalpis (Leicester, 1904)
magnus (Theobald, 1908)
menglaensis Dong, Zhou & Dong, 2002
omissus (Edwards, 1914)
pectinatus (Edwards, 1914)
pendulus (Edwards, 1914)
traubi Macdonald, 1960
vimoli Thurman & Thurman, 1958

Genus Eretmapodites

adami Ferrara & Eouzan, 1974
angolensis da Cunha Ramos & Ribeiro, 1992
argyrurus Edwards, 1936
brenguesi Rickenbach & Lombrici, 1975
brottesi Rickenbach, 1967
caillardi Rickenbach, Ferrara & Eouzan, 1968
chrysogaster Graham, 1909
corbeti Hamon, 1962
dracaenae Edwards, 1916
dundo da Cunha Ramos & Ribeiro, 1992
eouzani Rickenbach & Lombrici, 1974

ferrarai Rickenbach & Eouzan, 1970
forcipulatus Edwards, 1936
germaini Rickenbach & Eouzan, 1970
gilletti van Someren, 1949
grahami Edwards, 1911
grenieri Hamon & van Someren, 1961
haddowi van Someren, 1949
hamoni Grjebine, 1972
harperi van Someren, 1949
hightoni van Someren, 1947
inornatus Newstead, 1907
intermedius Edwards, 1936
jani Rickenbach & Lombrici, 1976
lacani Rickenbach & Eouzan, 1970
leucopous Graham, 1909
mahaffyi van Someren, 1949
marcellei Adam & Hamon, 1959
mattinglyi Hamon & van Someren, 1961
melanopous Graham, 1909
mortiauxi Cunha Ramos & Ribeiro, 1990
oedipodeios Graham, 1909
parvipluma Edwards, 1941
pauliani Grjebine, 1950
penicillatus Edwards, 1941
plioleucus Edwards, 1941
plioleucus brevis Edwards, 1941
productus Edwards, 1941
quinquevittatus Theobald, 1901†
ravissei Rickenbach & Eouzan, 1970
rickenbachi Ferrara & Eouzan, 1974
salauni Rickenbach, Ferrara & Eouzan, 1968
semisimplicipes Edwards, 1914
silvestris Ingram & de Meillon, 1927
silvestris conchobius Edwards, 1941
subsimplicipes Edwards, 1914
tendeiroi da Cunha Ramos, Ribeiro & Machado, 1992
tonsus Edwards, 1941
vansomereni Hamon, 1962
wansoni Edwards, 1941
wansoni douceti Adam & Hamon, 1959

Genus *Haemagogus*

Subgenus *Conopostegus* Dyar, 1925

clarki (Galindo, Carpenter & Trapido, 1953)
leucocelaenus (Dyar & Shannon, 1924)
leucophoebus (Galindo, Carpenter & Trapido, 1953)
leucotaeniatus (Komp, 1938)

Subgenus *Haemagogus* Williston, 1896

acutisentis Arnell, 1973
aeritinctus Galindo & Trapido, 1967
albomaculatus Theobald, 1903
anastasionis Dyar, 1921
andinus Osorno-Mesa, 1944
argyromeris Dyar & Ludlow, 1921
baresi Cerqueira, 1960
boshelli Osorno-Mesa, 1944
capricornii Lutz, 1904
celeste Dyar & Nuñez Tovar, 1927
chalcospilans Dyar, 1921
chrysochlorus Arnell, 1973
equinus Theobald, 1903
iridicolor Dyar, 1921
janthinomys Dyar, 1921
lucifer (Howard, Dyar & Knab, 1913)
mesodentatus Komp & Kumm, 1938
nebulosus Arnell, 1973
panarchys Dyar, 1921
regalis Dyar & Knab, 1906
soperi Levi-Castillo, 1955
spegazzinii Brethès, 1912
splendens Williston, 1896
tropicalis Cerqueira & Antunes, 1938

Genus *Heizmannia*

Subgenus *Heizmannia* Ludlow, 1905

aurea Brug, 1932
aureochaeta (Leicester, 1908)
carteri Amerasinghe, 1993
chandi Edwards, 1922
chengi Lien, 1968
communis (Leicester, 1908)
complex (Theobald, 1910)

covelli Barraud, 1929
demeilloni Mattingly, 1970
funerea (Leicester, 1908)
greenii (Theobald, 1905)
heterospina Gong & Lu, 1986
himalayensis Edwards, 1922
indica (Theobald, 1905)
kana Tanaka, Mizusawa & Saugstad, 1979
kanhsienensis Tung, 1955
lii Wu, 1936
macdonaldi Mattingly, 1957
mattinglyi Thurman, 1959
menglianensis Lu & Gong, 1986
menglianeroides Dong, Dong & Zhou, 2003
persimilis Mattingly, 1970
propinqua Mattingly, 1970
proxima Mattingly, 1970
reidi Mattingly, 1957
ruiliensis Dong, Zhou & Wang, 1997
scanloni Mattingly, 1970
scintillans Ludlow, 1905
taiwanensis Lien, 1968
tengchongensis Dong, Wang & Zhou, 2002
thelmae Mattingly, 1970
viridis Barraud, 1929

Subgenus *Mattinglyia* Lien, 1968

achaetae (Leicester, 1908)
catesi (Lien, 1968)
discrepans (Edwards, 1922)
lui Gong & Li, 1999
occidentayunnana (Gong & Lu, 1991)
tripunctata (Theobald, 1908)

Genus *Opifex*

Subgenus *Nothoskusea* Dumbleton, 1962
chathamicus (Dubleton, 1962)

Subgenus *Opifex* Hutton, 1902
fuscus Hutton, 1902

Genus *Psorophora*

Subgenus *Grabhamia* Theobald, 1903

cingulata (Fabricius, 1805)
columbiae (Dyar & Knab, 1906)
confinnis (Lynch Arribálzaga, 1891)
dimidiata Cerqueira, 1943
discolor (Coquillett, 1903)
infinis (Dyar & Knab, 1906)
insularia (Dyar & Knab, 1906)
jamaicensis Theobald, 1901
leucocnemis Martini, 1931
paulli Paterson & Shannon, 1927
pruinosa Martini, 1935
pygmaea (Theobald, 1903)
santamarinai Broche, 2000
signipennis (Coquillett, 1904)
varinervis Edwards, 1922

Subgenus *Janthinosoma* Lynch Arribálzaga, 1891

albigenu (Peryassú, 1908)
albipes (Theobald, 1907)
amazonica Cerqueira, 1960
champerico (Dyar & Knab, 1906)
circumflava Cerqueira, 1943
cyanescens (Coquillett, 1902)
discrucians (Walker, 1856)
ferox (von Humboldt, 1819)
fiebrigi Edwards, 1922
forceps Cerqueira, 1939
horrida (Dyar & Knab, 1908)
johnstonii (Grabham, 1905)
lanei Shannon & Cerqueira, 1943
longipalpus Randolph & O'Neill, 1944
lutzii (Theobald, 1901)
mathesoni Belkin & Heinemann, 1975
melanota Cerqueira, 1943
mexicana (Bellardi, 1859)
pilosa Duret, 1971
pseudoalbipes Duret, 1971
pseudomelanota Barata & Cotrim, 1971
totonaci Lassmann, 1951
varipes (Coquillett, 1904)

Subgenus *Psorophora* Robineau-Desvoidy, 1827

ciliata (Fabricius, 1794)
cilipes (Fabricius, 1805)
holmbergii Lynch Arribálzaga, 1891
howardii Coquillett, 1901
lineata (von Humboldt, 1819)
ochripes (Macquart, 1850)
pallescens Edwards, 1922
pilipes (Macquart, 1834)
saeva Dyar & Knab, 1906
stonei Vargas, 1956

Nomina dubia
goeldii (Giles, 1904)
marmorata (Philippi, 1865)
perterrens (Walker, 1856)

Genus *Udaya* Thurman, 1954

argyrurus (Edwards, 1934)
lucaris Macdonald & Mattingly, 1960
subsimilis (Barraud, 1927)

Genus *Verrallina*

Subgenus *Harbachius* Reinert, 1999

abdita (Barraud, 1931)
consonensis (Reinert, 1973)
fragilis Leicester, 1908
hamistylus (Laffoon, 1946)
indecorabilis Leicester, 1908
nobukonis (Yamada, 1932)
pahangi (Delfinado, 1968)
ramalingami (Reinert, 1974)
robertsi (Laffoon, 1946)
srilankensis (Reinert, 1977)
stunga (Klein, 1973)
uniformis (Theobald, 1910)
yusafi (Barraud, 1931)

Subgenus *Neomacleaya* Theobald, 1907

adusta (Laffoon, 1946)
agrestis (Barraud, 1931)
andamanensis (Edwards, 1922)
assamensis Bhattacharyya, Tewari, Prakash, Mohapatra & Mahanta, 2004
atriisimilis (Tanaka & Mizusawa, 1973)
atria (Barraud, 1928)
campylostylus (Laffoon, 1946)
cauta (Barraud, 1928)
clavata (Barraud, 1931)
comata (Barraud, 1931)
comosa (Reinert, 1974)
cretata (Delfinado, 1967)
cyrtolabis (Edwards, 1928)
gibbosa (Delfinado, 1967)
harrisonica (Reinert, 1974)
hispida (Delfinado, 1967)
incertus (Edwards, 1922)
indica (Theobald, 1907)
johnsoni (Laffoon, 1946)
johorensis (Reinert, 1974)
komponga (Klein, 1973)
lankaensis (Stone & Knight, 1958)
latipennis (Delfinado, 1967)
leicesteri (Edwards, 1917)
macrodixoa (Dyar & Shannon, 1925)
margarsen (Dyar & Shannon, 1925)
neomacrodixoa (King & Hoogstraal, 1947)
nigrotarsis (Ludlow, 1908)
notabilis (Delfinado, 1967)
nubicola (Laffoon, 1946)
panayensis (Ludlow, 1914)
petroelephantus (Wijesundara, 1951)
philippinensis (Delfinado, 1968)
phnoma (Klein, 1973)
prioekanensis (Brug, 1931)
protuberans (Delfinado, 1967)
pseudodiurna (Theobald, 1910)
pseudomediofasciata (Theobald, 1910)
pseudovarietas (Reinert, 1974)
rami (Barraud, 1928)
rara (Delfinado, 1968)
sabahensis (Reinert, 1974)
seculata (Menon, 1950)

singularis (Leicester, 1908)
sohni (Reinert, 1974)
spermathecus (Wijesundara, 1951)
torosa (Delfinado, 1967)
uncus (Theobald, 190l)
vallistris (Barraud, 1928)
varietas (Leicester, 1908)
virilis Leicester, 1908
yerburyi (Edwards, 1917)

Subgenus *Verrallina* Theobald, 1903

azureosquamata (Bonne-Wepster, 1948)
bifoliata (King & Hoogstraal, 1947)
butleri (Theobald, 1901)
carmenti (Edwards, 1924)
cuccioi (Belkin, 1962)
cunninghami (Taylor, 1944)
dux (Dyar & Shannon, 1925)
embiensis (Huang, 1968)
foliformis (King & Hoogstraal, 1947)
funerea (Theobald, 1903)
iriomotensis (Tanaka & Mizusawa, 1973)
killertonis (Huang, 1968)
leilae (King & Hoogstraal, 1947)
lineata (Taylor, 1914)
lugubris (Barraud, 1928)
mccormicki (Belkin, 1962)
milnensis (King & Hoogstraal, 1947)
multifolium (King & Hoogstraal, 1947)
obsoleta (Huang, 1968)
parasimilis (King & Hoogstraal, 1947)
pipkini (Bohart, 1957)
quadrifolium (Brug, 1934)
quadrispinata (King & Hoogstraal, 1947)
reesi (King & Hoogstraal, 1947)
sentania (King & Hoogstraal, 1947)
similis (Theobald, 1910)
simpla (King & Hoogstraal, 1947)
trispinata (King & Hoogstraal, 1947)
vanapa (Huang, 1968)
variabilis (Huang, 1968)

Nomen dubium

perdita Leicester, 1908

Genus *Zeugnomyia* Leicester, 1908

aguilari Baisas & Feliciano, 1953
fajardoi Baisas & Feliciano, 1953
gracilis Leicester, 1908
lawtoni Baisas, 1946
