# Supplementary material for: Making Mosquito Taxonomy Useful: A Stable Classification of Tribe Aedini that Balances Utility with Current Knowledge of Evolutionary Relationships
Source: PLoS One. 2015 Jul 30;10(7):e0133602. doi: 10.1371/journal.pone.0133602 (PMC4520491; doi:10.1371/journal.pone.0133602)
Supplement: S2 Appendix — (PDF) [file pone.0133602.s002.pdf]

Table XX. Proposed Informal species groups listed as subgenera in Reinert et al. (2009)

Reinert et al. (2009)	Herein: genus / subgenus	Suggested informal group prefix or previous group name	Species
Collessius (*Alloeomyia*)	*Aedes* (*Collessius*)	Alloeomyia	*banksi* Edwards
			pseudotaeniatus (Giles)
			tonkinensis Galliard & Ngu
Collessius (*Collessius*)	*Aedes* (*Collessius*)	Collessius	*elsiae* (Barraud)
			elsiae vicarious Lien
			hatorii Yamada
			macdougalli Edwards
			macfarlanei (Edwards)
			ningheensis Lei
			shortti (Barraud)
Georgecraigius (*Georgecraigius*)	*Aedes* (*Georgecraigius*)	Georgecraigius	*atropalpus* (Coquillet)
			epactius Dyar & Knab
Georgecraigius (*Horsfallius*)	*Aedes* (*Georgecraigius*)	Horsfallius	*fluviatilis* (Lutz)
Hopkinsius (*Hopkinsius*)	*Aedes* (*Hopkinsius*)	Hopkinsius	*barnardi* Edwards
			embuensis Edwards
			ingrami Edwards
			nyasae Edwards
			wellmanii (Theobald)
Hopkinsius (*Yamada*)	*Aedes* (*Hopkinsius*)	Yamada	*albocinctus* (Barraud)
			seoulensis Yamada
Macleaya (*Chaetocruiomyia*)	*Aedes* (*Macleaya*)	Chaetocruiomyia	*calabyi* Marks
			elchoensis Taylor

			humeralis Edwards
			macmillani Marks
			moloiensis Taylor
			spinosipes Edwards
			tulliae (Taylor)
			wattensis Taylor
Macleaya (*Macleaya*)	*Aedes* (*Macleaya*)	Macleaya	*littlechildi* Taylor
			stoneorum Marks
			tremulus (Theobald)
Mucidus (*Mucidus*)	*Aedes* (*Mucidus*)	Mucidus	*alternans* (Westwood)
			ferinus Knight
			grahamii (Theobald)
			laniger (Wiedemann)
			lucianus Muspratt
			mucidus (Karsch)
			nigerrimus (Theobald)
			quasiferinus Mattingly
			scatophagoides (Theobald)
			sudanensis (Theobald)
			tonkingi Gebert
Mucidus (*Pardomyia*)	*Aedes* (*Mucidus*)	Pardomyia	*aurantius* (Theobald)
			aurantius chrysogaster (Taylor)
			nigrescens (Edwards)
			quadripunctis (Ludlow)
Ochlerotatus (*Buvirilia*)	*Aedes* (*Ochlerotatus*)	Buvirilia	*edgari* Stone & Rosen
Ochlerotatus (*Crysoconops*)	*Aedes* (*Ochlerotatus*)	Chrysoconops	*bimaculatus* (Coquillett)
			fulvus (Wiedemann)
			fulvus pallens (Ross)

			jorgi Carpintero & Lequizamón
			pennai Antunes & Lane
			stigmaticus Edwards
Ochlerotatus (*Culicada*)	*Aedes* (*Ochlerotatus*)	Culicada	*canadensis* (Theobald)
			canadensis mathesoni Middlekauff
Ochlerotatus (*Culicelsa*)	*Aedes* (*Ochlerotatus*)	Culicelsa	*mitchellae* (Dyar)
			nigromaculis (Ludlow)
			sollicitans (Walker)
			taeniorhynchus (Wiedemann)
Ochlerotatus (*Empihals*)	*Aedes* (*Ochlerotatus*)	Empihals	*vigilax* (Skuse)
			vigilax ludlowae (R. Blanchard)
			vigilax vansomerenae Mattingly
Ochlerotatus (*Gilesia*)	*Aedes* (*Ochlerotatus*)	Gilesia	*aculeatus* (Theobald)
			mcdonaldi Belkin
			purpuraceus Brug
Ochlerotatus (*Juppius*)	*Aedes* (*Ochlerotatus*)	Juppius	*caballus* (Theobald)
			chelli (Edwards)
			juppi McIntosh
Ochlerotatus (*Lepidokeneon*)	*Aedes* (*Ochlerotatus*)	Lepidokeneon	*spilotus* Marks
			stricklandi (Edwards)
			turneri Marks
Ochlerotatus (*Ochlerotatus*)	*Aedes* (*Ochlerotatus*)	Ochlerotatus	*angustivittatus* Dyar & Knab
			atactavittatus Arnell
			auratus Grabham
			bogotanus (Arnell)

			comitatus Arnell
			condolescens Dyar & Knab
			crinifer (Theobald)
			deficiens Arnell
			euplocamus Dyar & Knab
			incomptus Arnell
			infirmatus Dyar & Knab
			meprai Martinez & Prosen
			obturbator Dyar & Knab
			patersoni Shannon & del Ponte
			pectinatus Arnell
			phaeonotus (Arnell)
			raymondi del Ponte, Castro & Garcia
			rhyacophilus da Costa Lima
			scapularis (Rondani)
			synchytus Arnell
			thelcter Dyar
			tortilis (Theobald)
			trivittatus (Coquillett)
Ochlerotatus (*Pholeomyia*)	*Aedes* (*Ochlerotatus*)	Pholeomyia	*calcariae* Marks
			clelandi (Taylor)
			flavifrons (Skuse)
			purpuriventris Edwards
Ochlerotatus (*Protoculex*)	*Aedes* (*Ochlerotatus*)	Protoculex	*aenigmaticus* Cerqueira & Costa
			atlanticus Dyar & Knab
			dupreei (Coquillett)
			eucephalaeus Dyar
			hastatus Dyar

			nubilis Theobald
			oligopistus Dyar
			pertinax Grabham
			serratus (Theobald)
			tormentor Dyar & Knab
Ochlerotatus (*Pseudoskusea*)	*Aedes* (*Ochlerotatus*)	Pseudoskusea	*bancroftianus* Edwards
			culiciformis (Theobald)
			multiplex (Theobald)
			postspiraculosus Dobrotworsky
Ochlerotatus (*Rusticoidus*)	*Aedes* (*Ochlerotatus*)	Rusticoidus	*albescens* Edwards
			bicristatus Thurman & Winkler
			krymmontanus Alekseev
			lepidonotus Edwards
			provocans (Walker)
			quasirusticus Torres Cañamares
			refiki Medschid
			rusticus (Rossi)
			rusticus subtrichurus Martini
			subdiversus Martini
Ochlerotatus (*Woodius*)	*Aedes* (*Ochlerotatus*)	Woodius	*diantaeus* Howard, Dyar & Knab
			intrudens Dyar
Petermattinglyius (*Aglaonotus*)	*Aedes* (*Petermattinglyius*)	Aglaonotus	*whartoni* Mattingly
Petermattinglyius (*Petermattinglyius*)	*Aedes* (*Petermattinglyius*)	Petermattinglyius	*franciscoi* Mattingly
			iyengari Edwards

			punctipes Edwards
			scanloni Reinert
Stegomyia (*Actinothrix*)	*Aedes* (*Stegomyia*)	Actinothrix	*edwardsi* (Barraud)
			robinsoni Belkin
			seampi Huang
			tulagiensis Edwards
Stegomyia (*Bohartius*)	*Aedes* (*Stegomyia*)	Pandani Subgroup of Bohart (1957)	*agrihanensis* Bohart
			neopandani Bohart
			pandani Stone
			rotanus Bohart & Ingram
			saipanensis Stone
Stegomyia (*Heteraspidion*)	*Aedes* (*Stegomyia*)	Annandalei Subgroup of Huang (1977)	*annandalei* (Theobald)
			craggi (Barraud)
Stegomyia (*Huangmyia*)	*Aedes* (*Stegomyia*)	Mediopunctatus Subgroup of Huang (1977)	*malikuli* Huang
			mediopunctatus (Theobald)
			mediopunctatus sureilensis Barraud
			perplexus (Leicester)
Stegomyia (*Mukwaya*)	*Aedes* (*Stegomyia*)	Simpsoni Group of Huang (2004)	*bromeliae* (Theobald)
			gandaensis Huang
			josiahae Huang
			kivuensis Edwards
			lilii (Theobald)
			sampi Huang
			simpsoni (Theobald)
			strelitziae Muspratt
			subargenteus Edwards

			woodi Edwards
Stegomyia (*Stegomyia*)	*Aedes* (*Stegomyia*)	Aegypti Group of Huang (2004)	*aegypti* (Linnaeus)
			aegypti formsus (Walker)
Stegomyia (*Xyele*)	*Aedes* (*Stegomyia*)	Desmotes Subgroup of Huang (1977)	*desmotes* (Giles)
Stegomyia (*Zoromorphus*)	*Aedes* (*Stegomyia*)	Zoromorphus	*futunae* Belkin
