# Supplementary material for: Making Mosquito Taxonomy Useful: A Stable Classification of Tribe Aedini that Balances Utility with Current Knowledge of Evolutionary Relationships
Source: PLoS One. 2015 Jul 30;10(7):e0133602. doi: 10.1371/journal.pone.0133602 (PMC4520491; doi:10.1371/journal.pone.0133602)
Supplement: S3 Appendix — Based on: Reinert JF (2009) List of abbreviations for currently valid generic-level taxa in family Culicidae (Diptera). European Mosquito Bulletin 27: 68–76. (PDF) [file pone.0133602.s003.pdf]

S3 Appendix. Updated mosquito generic (two letters) and subgeneric (three letters) abbreviations. Based on: Reinert JF (2009) List of abbreviations for currently valid generic-level taxa in family Culicidae (Diptera). European Mosquito Bulletin 27: 68-76.

* = Used here for the first time

Subfamily Anophelinae

Genus *Anopheles* Meigen = *An*.
Subgenus *Anopheles* Meigen = *Ano*.
Subgenus *Baimaia* Harbach, Rattanarithikul & Harrison = *Bmi*.
Subgenus *Cellia* Theobald= *Cel*.
Subgenus *Kerteszia* Theobald = *Ker*.
Subgenus *Lophopodomyia* Antunes = *Lph*.
Subgenus *Nyssorhynchus* Blanchard = *Nys*.
Subgenus *Stethomyia* Theobald = *Ste*.
Genus *Bironella* Theobald = *Bi*.
Genus *Chagasia* Cruz = *Ch*.

Subfamily Culicinae

Tribe Aedeomyiini

Genus *Aedeomyia* Theobald = *Ad*.
Subgenus *Aedeomyia* Theobald = *Ady*.
Subgenus *Lepiothauma* Enderlein = *Lpi*.

Tribe Aedini

Genus *Aedes* Meigen = *Ae*.
Subgenus *Abraedes* Zavortink = *Abr*.
Subgenus *Acartomyia* Theobald = *Acy*.
Subgenus *Aedimorphus* Theobald = *Adm*.
Subgenus *Alanstonea* Mattingly = *Ala*.
Subgenus *Albuginosus* Reinert = *Alb*.
Subgenus *Ayurakitia* Thurman = *Ayu*.
Subgenus *Aztecaedes* Zavortink = *Azt*.
Subgenus *Belkinius* Reinert = *Blk*.
Subgenus *Bifidistylus* Reinert, Harbach & Kitching = *Bif*.*
Subgenus *Borichinda* Harbach & Rattanarithikul = *Bor.**
Subgenus *Bothaella* Reinert = *Bot*.
Subgenus *Bruceharrisonius* Reinert = *Brh*.

Subgenus *Cancraedes* Edwards = *Can*.
Subgenus *Catageiomyia* Theobald = *Cat*.*
Subgenus *Catatassomyia* Dyar & Shannon = *Cts*.*
Subgenus *Christophersiomyia* Barraud = *Chr*.
Subgenus *Collessius* Reinert, Harbach & Kitching = *Col*.
Subgenus *Cornetius* Huang = *Cor*.
Subgenus *Dahliana* Reinert, Harbach & Kitching = *Dah*.*
Subgenus *Danielsia* Theobald = *Dan*.*
Subgenus *Dendroskusea* Edwards = *Dsk*.*
Subgenus *Diceromyia* Theobald = *Dic*.
Subgenus *Dobrotworskyius* Reinert, Harbach & Kitching = *Dob*.*
Subgenus *Downsiomyia* Vargas = *Dow*.*
Subgenus *Edwardsaedes* Belkin = *Edw*.
Subgenus *Elpeytonius* Reinert, Harbach & Kitching = *Elp*.*
Subgenus *Finlaya* Theobald = *Fin*.
Subgenus *Fredwardsius* Reinert = *Fre*.
Subgenus *Georgecraigius* Reinert, Harbach & Kitching = *Grg*.*
Subgenus *Geoskusea* Edwards = *Geo*.
Subgenus *Gilesius* Reinert, Harbach & Kitching = *Gil*.*
Subgenus *Gymnometopa* Coquillett = *Gym*.
Subgenus *Halaedes* Belkin = *Hal*.
Subgenus *Himalaius* Reinert, Harbach & Kitching = *Him*.*
Subgenus *Hopkinsius* Reinert, Harbach & Kitching = *Hop*.*
Subgenus *Howardina* Theobald = *How*.
Subgenus *Huaedes* Huang = *Hua*.
Subgenus *Hulecoeteomyia* Theobald = *Hul*.*
Subgenus *Indusius* Edwards = *Ind*.
Subgenus *Isoaedes* Reinert = *Isa*.
Subgenus *Jarnellius* Reinert, Harbach & Kitching = *Jar*.*
Subgenus *Jihlienius* Reinert, Harbach & Kitching = *Jih*.*
Subgenus *Kenknightia* Reinert = *Ken*.
Subgenus *Kompia* Aitken = *Kom*.
Subgenus *Leptosomatomyia* Theobald = *Lep*.
Subgenus *Levua* Stone & Bohart = *Lev*.
Subgenus *Lewnielsenius* Reinert, Harbach & Kitching = *Lew*.
Subgenus *Lorrainea* Belkin = *Lor*.
Subgenus *Luius* Reinert, Harbach & Kitching = *Lui*.*
Subgenus *Macleaya* Theobald = *Mac*.
Subgenus *Molpemyia* Theobald = *Mol*.
Subgenus *Mucidus* Theobald = *Muc*.
Subgenus *Neomelaniconion* Newstead = *Neo*.
Subgenus *Nyctomyia* Harbach & Linton = *Nyc*.
Subgenus *Ochlerotatus* Lynch Arribálzaga = *Och*.
Subgenus *Paraedes* Edwards = *Par*.
Subgenus *Patmarksia* Reinert, Harbach & Kitching = *Pat*.*
Subgenus *Petermattinglyius* Reinert, Harbach & Kitching = *Pet*.*

Subgenus *Phagomyia* Theobald = *Phg*.*
Subgenus *Polyleptiomyia* Theobald = *Pmt*.*
Subgenus *Pseudarmigeres* Stone & Knight = *Psa*.
Subgenus *Rampamyia* Reinert, Harbach & Kitching = *Ram*.*
Subgenus *Rhinoskusea* Edwards = *Rhi*.
Subgenus *Sallumia* Reinert, Harbach & Kitching = *Sal*.
Subgenus *Scutomyia* Theobald = *Sct*.
Subgenus *Skusea* Theobald = *Sku*.
Subgenus *Stegomyia* Theobald = *Stg*.
Subgenus *Tanakaius* Reinert, Harbach & Kitching = *Tan*.*
Subgenus *Tewarius* Reinert = *Tew*.*
Subgenus *Vansomerenis* Reinert, Harbach & Kitching = *Van*.

Genus *Armigeres* Theobald = *Ar*.
Subgenus *Armigeres* Theobald = *Arm*.
Subgenus *Leicesteria* Theobald = *Lei*.

Genus *Eretmapodites* Theobald = *Er*.

Genus *Haemagogus* Williston = *Hg*.
Subgenus *Conopostegus* Dyar = *Con*.
Subgenus *Haemagogus* Williston = *Hag*.

Genus *Heizmannia* Ludlow = *Hz*.
Subgenus *Heizmannia* Ludlow = *Hez*.
Subgenus *Mattinglyia* Lien = *Mat*.

Genus *Opifex* Hutton = *Op*.
Subgenus *Nothoskusea* Dumbleton = *Not*.
Subgenus *Opifex* Hutton = *Opi*.

Genus *Psorophora* Robineau-Desvoidy = *Ps*.
Subgenus *Grabhamia* Theobald = *Gra*.
Subgenus *Janthinosoma* Lynch Arribálzaga = *Jan*.
Subgenus *Psorophora* Robineau-Desvoidy = *Pso*.

Genus *Udaya* Thurman = *Ud*.

Genus *Verrallina* Theobald = *Ve*.
Subgenus *Harbachius* Reinert= *Har*.
Subgenus *Neomacleaya* Theobald = *Nma*.
Subgenus *Verrallina* Theobald = *Ver*.

Genus *Zavortinkius* Reinert = *Za*.

Genus *Zeugnomyia* Leicester = *Ze*.

Tribe Culicini

Genus *Culex* Linnaeus = *Cx*.

Subgenus *Acalleomyia* Leicester = *Aca*.
Subgenus *Acallyntrum* Stone & Penn = *Acl*.
Subgenus *Aedinus* Lutz = *Ads*.
Subgenus *Afroculex* Danilov = *Afc*.
Subgenus *Allimanta* Casal & Garcia = *Alm*.
Subgenus *Anoedioporpa* Dyar = *And*.
Subgenus *Barraudius* Edwards = *Bar*.
Subgenus *Belkinomyia* Adames & Galindo = *Bel*.
Subgenus *Carrollia* Lutz = *Car*.
Subgenus *Culex* Linnaeus = *Cux*.
Subgenus *Culiciomyia* Theobald = *Cui*.
Subgenus *Eumelanomyia* Theobald = *Eum*.
Subgenus *Kitzmilleria* Danilov = *Kit*.
Subgenus *Lasiosiphon* Kirkpatrick = *Las*.
Subgenus *Lophoceraomyia* Theobald = *Lop*.
Subgenus *Maillotia* Theobald = *Mai*.
Subgenus *Melanoconion* Theobald = *Mel*.
Subgenus *Micraedes* Coquillett = *Mca*.
Subgenus *Microculex* Theobald = *Mcx*.
Subgenus *Neoculex* Dyar = *Ncx*.
Subgenus *Nicaromyia* Broche & Rodríguez Rodríguez = *Nic*.
Subgenus *Oculeomyia* Theobald = *Ocu*.
Subgenus *Phenacomyia* Harbach & Peyton = *Phc*.
Subgenus *Phytotelmatomyia* Rossi & Harbach = *Phy*.
Subgenus *Sirivanakarnius* Tanaka = *Sir*.
Subgenus *Tinolestes* Coquillett = *Tin*.

Genus *Deinocerites* Theobald = *De*.

Genus *Galindomyia* Stone & Barreto = *Ga*.

Genus *Lutzia* Theobald = *Lt.*

Subgenus *Insulalutzia* Tanaka = *Ilt*.
Subgenus *Metalutzia* Tanaka = *Mlt*.
Subgenus *Lutzia* Theobald = *Lut*.

Tribe Culisetini

Genus *Culiseta* Felt = *Cs*.

Subgenus *Allotheobaldia* Broelemann = *All*.
Subgenus *Austrotheobaldia* Dobrotworsky = *Aut*.
Subgenus *Climacura* Howard, Dyar & Knab = *Cli*.

Subgenus *Culicella* Felt = *Cuc*.
Subgenus *Culiseta* Felt = *Cus*.
Subgenus *Neotheobaldia* Dobrotworsky = *Net*.
Subgenus *Theomyia* Edwards = *Thm*.

Tribe Ficalbiini

Genus *Ficalbia* Theobald = *Fi*.

Genus *Mimomyia* Theobald = *Mi*.
Subgenus *Etorleptiomyia* Theobald = *Eto*.
Subgenus *Ingramia* Edwards = *Ing*.
Subgenus *Mimomyia* Theobald = *Mim*.

Tribe Hodgesiini

Genus *Hodgesia* Theobald = *Ho*.

Tribe Mansoniini

Genus *Coquillettidia* Dyar = *Cq*.
Subgenus *Austromansonia* Belkin = *Aus*.
Subgenus *Coquillettidia* Dyar = *Coq*.
Subgenus *Rhynchotaenia* Brethés = *Rhy*.

Genus *Mansonia* Blanchard = *Ma*.
Subgenus *Mansonia* Blanchard = *Man*.
Subgenus *Mansonioides* Theobald = *Mnd*.

Tribe Orthopodomyiini

Genus *Orthopodomyia* Theobald = *Or*.

Tribe Sabethini

Genus *Isostomyia* Coquillett = *Is*.

Genus *Johnbelkinia* Zavortink = *Jb*.

Genus *Kimia* Vu Duc Huong & Harbach = *Km*.

Genus *Limatus* Theobald = *Li*.

Genus *Malaya* Leicester = *Ml*.

Genus *Maorigoeldia* Edwards = *Mg*.

Genus *Onirion* Harbach & Peyton = *On*.

Genus *Runchomyia* Theobald = *Ru*.
- Subgenus *Ctenogoeldia* Edwards = *Cte*.
- Subgenus *Runchomyia* Theobald = *Run*.

Genus *Sabethes* Robineau-Desvoidy = *Sa*.
- Subgenus *Davismyia* Lane & Cerqueira = *Dav*.
- Subgenus *Peytonulus* Harbach = *Pey*.
- Subgenus *Sabethes* Robineau-Desvoidy = *Sab*.
- Subgenus *Sabethinus* Lutz = *Sbn*.
- Subgenus *Sabethoides* Theobald = *Sbo*.

Genus *Shannoniana* Lane & Cerqueira = *Sh*.

Genus *Topomyia* Leicester = *To*.
- Subgenus *Miyagiella* Harbach = *Myg*.
- Subgenus *Suaymyia* Thurman = *Sua*.
- Subgenus *Topomyia* Leicester = *Top*.

Genus *Trichoprosopon* Theobald = *Tr*.

Genus *Tripteroides* Giles = *Tp*.
- Subgenus *Polylepidomyia* Theobald = *Pol*.
- Subgenus *Rachionotomyia* Theobald = *Rah*.
- Subgenus *Rachisoura* Theobald = *Rac*.
- Subgenus *Tricholeptomyia* Dyar & Shannon = *Tri*.
- Subgenus *Tripteroides* Giles = *Trp*.

Genus *Wyeomyia* Theobald = *Wy*.
- Subgenus *Antunesmyia* Lane & Cerqueira = *Ant*.
- Subgenus *Caenomyiella* Harbach & Peyton = *Cae*.
- Subgenus *Cruzmyia* Lane & Cerqueira = *Cru*.
- Subgenus *Decamyia* Dyar = *Dec*.
- Subgenus *Dendromyia* Theobald = *Den*.
- Subgenus *Dodecamyia* Dyar = *Dod*.
- Subgenus *Exallomyia* Harbach & Peyton = *Exm*.
- Subgenus *Hystatomyia* Dyar = *Hys*.
- Subgenus *Menolepis* Lutz = *Men*.
- Subgenus *Nunezia* Dyar = *Nuz*.
- Subgenus *Phoniomyia* Theobald = *Pho*.
- Subgenus *Prosopolepis* Lutz = *Prl*.
- Subgenus *Spilonympha* Motta & Lourenço-de-Oliveira = *Spi*.
- Subgenus *Wyeomyia* Theobald = *Wyo*.
- Subgenus *Zinzala* Zavortink = *Zin*.

Tribe Toxorhynchitini

Genus Toxorhynchites Theobald = *Tx*.
Subgenus *Afrorhynchus* Ribeiro = *Afr*.
Subgenus *Ankylorhynchus* Lutz = *Ank*.
Subgenus *Lynchiella* Lahille = *Lyn*.
Subgenus *Toxorhynchites* Theobald = *Tox*.

Tribe Uranotaeniini

Genus *Uranotaenia* Lynch Arribálzaga = *Ur*.
Subgenus *Pseudoficalbia* Theobald = *Pfc*.
Subgenus *Uranotaenia* Lynch Arribálzaga = *Ura*.
