# Supplementary material for: Making Mosquito Taxonomy Useful: A Stable Classification of Tribe Aedini that Balances Utility with Current Knowledge of Evolutionary Relationships
Source: PLoS One. 2015 Jul 30;10(7):e0133602. doi: 10.1371/journal.pone.0133602 (PMC4520491; doi:10.1371/journal.pone.0133602)
Supplement: S1 Fig — Valid generic level names and graphical timeline of nomenclature combination changes in Aedine taxa pre-2000 to date. Genera are indicated in blue, subgenera in green and synonomy (Syn.) in red. Author abbreviations include: D&S = Dyar and Shannon; H&R = Harbach & Rattanarithikul; R-D = Robineau-Desvoidy RH&K = Reinert, Harbach & Kitching; S&P = Shevchenko & Prudkina. Generic abbreviations follow those in Reinert, Harbach & Kitching (2009) and herein as listed in S3 Appendix. *Nyctomyia is a replacement name for the pre-occupied genus name Nyx (Harbach et al., Zootaxa, 3683(2), 159–177 (2013). (PDF) [file pone.0133602.s004.pdf]

S1 Fig. Valid generic level names and graphical timeline of nomenclature combination changes in Aedine taxa pre-2000 to date. Genera are indicated in blue, subgenera in green and synonomy (Syn.) in red. Author abbreviations include: D & S = Dyar and Shannon; H & R = Harbach & Rattanarithikul; R-D = Robineau-Desvoidy R, H & K = Reinert, Harbach & Kitching; S & P = Shevchenko & Prudkina. Generic abbreviations follow those in Reinert, Harbach & Kitching (2009) and herein as listed in S3 Appendix. **Nyctomyia* is a replacement name for the originally pre-occupied genus name *Nyx* (Harbach *et al*., *Zootaxa*, 3683(2), 159-177 (2013).

Aedini valid generic level names	Pre-2000	2000	2003	2004	2006	2007	2008	2009	2013	Herein
Abraedes Zavortink 1970	*Ae.* (*Abr.*)	*Oc.* (*Abr.*)		*Abraedes*						*Aedes* (*Abraedes*)
Acartomyia Theobald 1903	Syn. *Ae.* (*Och.*)						*Oc.* (*Acy.*)	*Acartomyia*		*Aedes* (*Acartomyia*)
Aedes Meigen 1818	*Ae.*									*Aedes*
Aedimorphus Theobald 1903	*Ae.* (*Adm.*)							*Aedimorphus*		*Aedes* (*Aedimorphus*)
Aglaonotus R, H & K 2009								*Petermattinglius* (*Aglaonotus*)		Syn. *Aedes* (*Petermattinglius*)
Alloeomyia R, H & K 2008							*Collesius* (*Alloeomyia*)			Syn. *Aedes* (*Collessius*)
Alanstonea Mattingly 1960	*Ae.* (*Ala.*)			*Alanstonea*						*Aedes* (*Alanstonea*)
Albuginosus Reinert 1987	*Ae.* (*Alb.*)			*Albuginosus*						*Aedes* (*Albuginosus*)
Armigeres Theobald 1901	*Armigeres*									*Armigeres*
Ayurakitia Thurman 1954	*Ae.* (*Ayu.*)	*Ayurakitia*								*Aedes* (*Ayurakitia*)
Aztecaedes Zavortink 1972	*Ae.* (*Azt.*)	*Oc.* (*Azt.*)		*Aztecaedes*						*Aedes* (*Aztecaedes*)
Belkinius Reinert 1982	*Ae.* (*Blk.*)			*Belkinius*						*Aedes* (*Belkinius*)
Bifidistylus R, H & K 2009								*Bifidistylus*		*Aedes* (*Bifidistylus*)
Bohartius R, H & K 2009								*Stegomyia* (*Bohartius*)		Syn. *Aedes* (*Stegomyia*)
Borichinda H & R 2007						*Borichinda*				*Aedes* (*Borichinda*)
Bothaella Reinert 1973	*Ae.* (*Bot.*)			*Bothaella*						*Aedes* (*Bothaella*)
Bruceharrisonius Reinert 2003			*Oc.* (*Brh.*)		*Bruceharrisonius*					*Aedes* (*Bruceharrisonius*)
Buvirilia R, H & K 2008							*Oc.* (*Buvirilia*)			Syn. *Aedes* (*Ochlerotatus*)
Cancraedes Edwards 1929	*Ae.* (*Can.*)							*Cancraedes*		*Aedes* (*Cancraedes*)
Catageiomyia Theobald 1903	Syn. *Ae.* (*Adm.*)							*Catageiomyia*		*Aedes* (*Catageiomyia*)
Catatassomyia D & S 1925	Syn. *Ae.* (*Stg.*)							*Catatassomyia*		*Aedes* (*Catatassomyia*)
Chaetocruiomyia Theobald 1910	*Ae.* (*Cha.*)	*Oc.* (*Chaetocruiomyia*)								Syn. *Aedes* (*Ochlerotatus*)
Christophersiomyia Barraud 1923	*Ae.* (*Chr.*)			*Christophersiomyia*						*Aedes* (*Christophersiomyia*)
Chrysoconops Goeldi 1905	Syn. *Ae.* (*Och.*)						*Oc.* (*Chrysoconops*)			Syn. *Aedes* (*Ochlerotatus*)
Collessius R, H & K 2006					*Collessius*					*Aedes* (*Collessius*)

<table>
<tr><th>Aedini valid generic level names</th><th>Pre-2000</th><th>2000</th><th>2003</th><th>2004</th><th>2006</th><th>2007</th><th>2008</th><th>2009</th><th>2013</th><th>Herein</th></tr>
<tr><td>Conopostegus
Dyar 1925</td><td colspan="9">Haemagogus (Conopostegus)</td><td>Haemagogus (Conopostegus)</td></tr>
<tr><td>Cornetius
Huang 2005</td><td colspan="7"></td><td colspan="2">Cornetius</td><td>Aedes (Cornetius)</td></tr>
<tr><td>Culicada
Felt 1904</td><td colspan="7">Syn. Ae. (Och.)</td><td colspan="2">Oc. (Culicada)</td><td>Syn. Aedes (Ochlerotatus)</td></tr>
<tr><td>Culicelsa
Felt 1904</td><td colspan="6">Syn. Ae. (Och.)</td><td colspan="3">Oc. (Culicelsa)</td><td>Syn. Aedes (Ochlerotatus)</td></tr>
<tr><td>Dahliana
R, H & K 2006</td><td colspan="4"></td><td colspan="5">Dahliana</td><td>Aedes (Dahliana)</td></tr>
<tr><td>Danielsia
Theobald 1904</td><td colspan="6">Syn. Ae. (Fin.)</td><td colspan="3">Danielsia</td><td>Aedes (Danielsia)</td></tr>
<tr><td>Dendroskusea
Edwards 1929</td><td colspan="7">Syn. Ae. (Dic.)</td><td colspan="2">Dendroskusea</td><td>Aedes (Dendroskusea)</td></tr>
<tr><td>Diceromyia
Theobald 1911</td><td colspan="3">Ae. (Dic.)</td><td colspan="6">Diceromyia</td><td>Aedes (Diceromyia)</td></tr>
<tr><td>Dobrotworskyius
R, H & K 2006</td><td colspan="4"></td><td colspan="5">Dobrotworskyius</td><td>Aedes (Dobrotworskyius)</td></tr>
<tr><td>Downsiomyia
Vargas 1950</td><td colspan="3">Syn. Ae. (Fin.)</td><td colspan="6">Downsiomyia</td><td>Aedes (Downsiomyia)</td></tr>
<tr><td>Edwardsaedes
Belkin 1962</td><td colspan="3">Ae. (Edw.)</td><td colspan="6">Edwardsaedes</td><td>Aedes (Edwardsaedes)</td></tr>
<tr><td>Elpeytonius
R, H & K 2009</td><td colspan="7"></td><td colspan="2">Elpeytonius</td><td>Aedes (Elpeytonius)</td></tr>
<tr><td>Empihals
R, H & K 2008</td><td colspan="6"></td><td colspan="3">Oc. (Empihals)</td><td>Syn. Aedes (Ochlerotatus)</td></tr>
<tr><td>Eretmapodites
Theobald 1901</td><td colspan="9">Eretmapodites</td><td>Eretmapodites</td></tr>
<tr><td>Finlaya
Theobald 1903</td><td>Ae. (Fin.)</td><td colspan="2">Oc. (Fin.)</td><td colspan="6">Finlaya</td><td>Aedes (Finlaya)</td></tr>
<tr><td>Fredwardsius
Reinert 2000</td><td></td><td colspan="2">Ae. (Fre.)</td><td colspan="6">Fredwardsius</td><td>Aedes (Fredwardsius)</td></tr>
<tr><td>Georgecraigius
R, H & K 2006</td><td colspan="4"></td><td colspan="5">Georgecraigius</td><td>Aedes (Georgecraigius)</td></tr>
<tr><td>Geoskusea
Edwards 1929</td><td>Ae. (Geo.)</td><td colspan="3">Oc. (Geo.)</td><td colspan="2">Geoskusea</td><td>Oc. (Geo.)</td><td colspan="2">Geoskusea</td><td>Aedes (Geoskusea)</td></tr>
<tr><td>Gilesia
Theobald 1903</td><td colspan="6">Syn. Ae. (Och.)</td><td colspan="3">Oc. (Gilesia)</td><td>Syn. Aedes (Ochlerotatus)</td></tr>
<tr><td>Gilesius
R, H & K 2006</td><td colspan="4"></td><td colspan="5">Gilesius</td><td>Aedes (Gilesius)</td></tr>
<tr><td>Grabhamia
Theobald 1903</td><td colspan="9">Psorophora (Grabhamia)</td><td>Psorophora (Grabhamia)</td></tr>
<tr><td>Gymnometopa
Coquillett 1906</td><td>Ae. (Gym.)</td><td colspan="2">Oc. (Gym.)</td><td colspan="6">Gymnometopa</td><td>Aedes (Gymnometopa)</td></tr>
<tr><td>Haemagogus
Williston 1896</td><td colspan="9">Haemagogus</td><td>Haemagogus</td></tr>
<tr><td>Halaedes
Belkin 1962</td><td>Ae. (Hal.)</td><td colspan="2">Oc. (Hal.)</td><td colspan="6">Halaedes</td><td>Aedes (Halaedes)</td></tr>
<tr><td>Heizmannia
Ludlow 1905</td><td colspan="9">Heizmannia</td><td>Heizmannia</td></tr>
<tr><td>Heteraspidion
R, H & K 2009</td><td colspan="7"></td><td colspan="2">Stegomyia (Heteraspidion)</td><td>Syn. Aedes (Stegomyia)</td></tr>
</table>

**Aedini valid generic level names**	**Pre-2000**	**2000**	**2003**	**2004**	**2006**	**2007**	**2008**	**2009**	**2013**	**Herein**
Himalaius R, H & K 2006					*Himalaius*					*Aedes* (*Himalaius*)
Hopkinsius R, H & K 2008							*Hopkinsius*			*Aedes* (*Hopkinsius*)
Horsfallius R, H & K 2006					*Georgecraigius* (*Horsfallius*)					Syn. *Aedes* (*Georgecraigius*)
Howardina Theobald 1903	*Ae.* (*How.*)	*Oc.* (*How.*)		*Howardina*						*Aedes* (*Howardina*)
Huaedes Huang 1968	*Ae.* (*Hua.*)			*Huaedes*						*Aedes* (*Huaedes*)
Huangmyia R, H & K 2009								*Stegomyia* (*Huangmyia*)		Syn. *Aedes* (*Stegomyia*)
Hulecoeteomyia Theobald 1904	Syn. *Ae.* (*Fin.*)				*Hulecoeteomyia*					*Aedes* (*Hulecoeteomyia*)
Indusius Edwards 1934	*Ae.* (*Ind.*)			*Indusius*						*Aedes* (*Indusius*)
Isoaedes Reinert 1979	*Ae.* (*Isa.*)			*Isoaedes*						*Aedes* (*Isoaedes*)
Janthinosoma *Lynch-Arribalzaga 1891*	*Psorophora* (*Janthinsoma*)									*Psorophora* (*Janthinosoma*)
Jarnellius *R, H & K 2006*					*Jarnellius*					*Aedes* (*Jarnellius*)
Jihlienius *R, H & K 2006*					*Jihlienius*					*Aedes* (*Jihlienius*)
Juppius R, H & K 2009								*Oc.* (*Juppius*)		Syn. *Aedes* (*Ochlerotatus*)
Kenknightia Reinert 1990	*Ae.* (*Ken.*)	*Oc.* (*Ken.*)		*Kenknightia*						*Aedes* (*Kenknightia*)
Kompia Aitken 1941	*Ae.* (*Kom.*)	*Oc.* (*Kom.*)		*Kompia*						*Aedes* (*Kompia*)
Leicesteria Theobald 1904	*Armigeres* (*Leicesteria*)									*Armigeres* (*Leicesteria*)
Lepidokeneon R, H & K 2009								*Oc.* (*Lepidokeneon*)		Syn. *Aedes* (*Ochlerotatus*)
Leptosomatomyia Theobald 1905	*Ae.* (*Lep.*)			*Leptosomatomyia*						*Aedes* (*Leptosomatomyia*)
Levua Stone & Bohart 1944	*Ae.* (*Lev.*)	*Oc.* (*Lev.*)		*Levua*			*Oc.* (*Lev.*)	*Levua*		*Aedes* (*Levua*)
Lewnielsenius R, H & K 2006					*Jarnellius* (*Lew.*)			*Lewnielsenius*		*Aedes* (*Lewnielsenius*)
Lorrainea Belkin 1962	*Ae.* (*Lor.*)			*Lorrainea*						*Aedes* (*Lorrainea*)
Luius R, H & K 2008							*Luius*			*Aedes* (*Luius*)
Macleaya Theobald 1903	*Ae.* (*Mac.*)	*Oc.* (*Mac.*)			*Macleaya*					*Aedes* (*Macleaya*)
Mattinglyia Lien 1968	*Heizmannia* (*Mattinglyia*)									*Heizmannia* (*Mattinglyia*)
Molpemyia Theobald 1910	Syn. *Ae.* (*Fin.*)	*Oc.* (*Mol.*)			*Molpeymia*					*Aedes* (*Molpemyia*)
Mucidus Theobald 1901	*Ae.* (*Muc.*)	*Oc.* (*Muc.*)		*Mucidus*						*Aedes* (*Mucidus*)

<table>
<tr><th>Aedini valid generic level names</th><th>Pre-2000</th><th>2000</th><th>2003</th><th>2004</th><th>2006</th><th>2007</th><th>2008</th><th>2009</th><th>2013</th><th>Herein</th></tr>
<tr><td>Mukwaya R, H & K 2009</td><td colspan="7"></td><td colspan="2">Stegomyia (Mukwaya)</td><td>Syn. Aedes (Stegomyia)</td></tr>
<tr><td>Neomacleaya Theobald 1907</td><td colspan="9">Verrallina (Neomacleaya)</td><td>Verrallina (Neomacleaya)</td></tr>
<tr><td>Neomelaniconion Newstead 1907</td><td colspan="3">Ae. (Neo.)</td><td colspan="6">Neomelanoconion</td><td>Aedes (Neomelanoconion)</td></tr>
<tr><td>Nothoskusea Dumbleton 1962</td><td>Ae. (Not.)</td><td colspan="2">Oc. (Not.)</td><td colspan="6">Opifex (Not.)</td><td>Opifex (Nothoskusea)</td></tr>
<tr><td>Nyctomyia Harbach & Linton 2013</td><td colspan="8"></td><td>Nyctomyia*</td><td>Aedes (Nyctomyia)</td></tr>
<tr><td>Ochlerotatus Lynch-Arribalzaga 1891</td><td>Ae. (Och.)</td><td colspan="8">Ochlerotatus</td><td>Aedes (Ochlerotatus)</td></tr>
<tr><td>Opifex Hutton 1902</td><td colspan="9">Opifex</td><td>Opifex</td></tr>
<tr><td>Paraedes Edwards 1934</td><td colspan="3">Ae. (Par.)</td><td colspan="6">Paraedes</td><td>Aedes (Paraedes)</td></tr>
<tr><td>Pardomyia Theobald 1907</td><td colspan="4">Syn. Ae. (Muc.)</td><td colspan="5">Mucidus (Pardomyia)</td><td>Syn. Aedes (Mucidus)</td></tr>
<tr><td>Patmarksia R, H & K 2006</td><td colspan="4"></td><td colspan="5">Patmarksia</td><td>Aedes (Patmarksia)</td></tr>
<tr><td>Petermattinglius R, H & K 2009</td><td colspan="7"></td><td colspan="2">Petermattinglius</td><td>Aedes (Petermattinglius)</td></tr>
<tr><td>Phagomyia Theobald 1905</td><td colspan="4">Syn. Ae. (Fin.)</td><td colspan="5">Phagomyia</td><td>Aedes (Phagomyia)</td></tr>
<tr><td>Pholeomyia R, H & K 2008</td><td colspan="6"></td><td colspan="3">Oc. (Pholeomyia)</td><td>Syn. Aedes (Ochlerotatus)</td></tr>
<tr><td>Polyleptiomyia Theobald 1905</td><td colspan="7">Syn. Ae. (Adm.)</td><td colspan="2">Polyleptiomyia</td><td>Aedes (Polyleptiomyia)</td></tr>
<tr><td>Protoculex Felt 1904</td><td colspan="6">Syn. Ae. (Och.)</td><td colspan="3">Oc. (Protoculex)</td><td>Syn. Aedes (Ochlerotatus)</td></tr>
<tr><td>Protomacleaya Theobald 1907</td><td>Ae. (Pro.)</td><td colspan="8">Oc. (Protomacleaya)</td><td>Syn. Aedes (Ochlerotatus)</td></tr>
<tr><td>Pseudarmigeres Stone & Knight 1956</td><td colspan="3">Ae. (Pse.)</td><td colspan="6">Pseudarmigeres</td><td>Aedes (Pseudarmigeres)</td></tr>
<tr><td>Pseudoskusea Theobald 1907</td><td>Ae. (Psk.)</td><td colspan="3">Oc. (Psk.)</td><td colspan="2">Pseudoskusea</td><td colspan="3">Oc.(Psk.)</td><td>Syn. Aedes (Ochlerotatus)</td></tr>
<tr><td>Psorophora R-D 1827</td><td colspan="9">Psorophora</td><td>Psorophora</td></tr>
<tr><td>Rampamyia R, H & K 2006</td><td colspan="4"></td><td colspan="5">Rampamyia</td><td>Aedes (Rampamyia)</td></tr>
<tr><td>Rhinoskusea Edwards 1929</td><td>Ae. (Rhi.)</td><td colspan="2">Oc. (Rhi.)</td><td colspan="6">Rhinoskusea</td><td>Aedes (Rhinoskusea)</td></tr>
<tr><td>Rusticoidus S & P 1973</td><td>Ae. (Rus.)</td><td colspan="8">Oc. (Rus.)</td><td>Syn. Aedes (Ochlerotatus)</td></tr>
<tr><td>Sallumia R, H & K 2008</td><td colspan="6"></td><td>Oc. (Sal.)</td><td colspan="2">Sallumia</td><td>Aedes (Sallumia)</td></tr>
<tr><td>Scutomyia Theobald 1904</td><td colspan="3">Syn. Ae. (Stg.)</td><td colspan="6">Scutomyia</td><td>Aedes (Scutomyia)</td></tr>
<tr><td>Skusea Theobald 1903</td><td colspan="3">Ae. (Sku.)</td><td colspan="6">Skusea</td><td>Aedes (Skusea)</td></tr>
</table>

<table>
<tr><th>Aedini valid generic level names</th><th>Pre-2000</th><th>2000</th><th>2003</th><th>2004</th><th>2006</th><th>2007</th><th>2008</th><th>2009</th><th>2013</th><th>Herein</th></tr>
<tr><td>Stegomyia
Theobald 1901</td><td colspan="3">Ae. (Stg.)</td><td colspan="6">Stegomyia</td><td>Aedes (Stegomyia)</td></tr>
<tr><td>Tanakius
R, H & K 2004</td><td colspan="3"></td><td colspan="6">Tanakius</td><td>Aedes (Tanakius)</td></tr>
<tr><td>Tewarius
R, H & K 2006</td><td colspan="3"></td><td colspan="6">Tewarius</td><td>Aedes (Tewarius)</td></tr>
<tr><td>Udaya
Thurman 1954</td><td colspan="9">Udaya</td><td>Udaya</td></tr>
<tr><td>Vansomerenis
R, H & K 2006</td><td colspan="4"></td><td colspan="5">Vansomerensis</td><td>Aedes (Vansomerensis)</td></tr>
<tr><td>Verrallina
Theobald 1903</td><td colspan="9">Verrallina</td><td>Verrallina</td></tr>
<tr><td>Woodius
R, H & K 2009</td><td colspan="7"></td><td colspan="2">Oc. (Woodius)</td><td>Syn. Aedes (Ochlerotatus)</td></tr>
<tr><td>Xyele
R, H & K 2009</td><td colspan="7"></td><td colspan="2">Stegomyia (Xyele)</td><td>Syn. Aedes (Stegomyia)</td></tr>
<tr><td>Yamada
R, H & K 2008</td><td colspan="6"></td><td colspan="3">Hopkinsinsius (Yamada)</td><td>Syn. Aedes (Hopkinsius)</td></tr>
<tr><td>Zavortinkius
Reinert 1999</td><td>Ae. (Zav.)</td><td colspan="2">Oc. (Zav.)</td><td colspan="6">Zavortinkius</td><td>Aedes (Zavortinkius)</td></tr>
<tr><td>Zeugnomyia
Leicester 1908</td><td colspan="9">Zeugnomyia</td><td>Zeugnomyia</td></tr>
<tr><td>Zoomorphus
R, H & K 2009</td><td colspan="7"></td><td colspan="2">Ochlerotatus (Zoromorphus)</td><td>Syn. Aedes (Ochlerotatus)</td></tr>
</table>
