# Supplementary figures and images for: Making Mosquito Taxonomy Useful: A Stable Classification of Tribe Aedini that Balances Utility with Current Knowledge of Evolutionary Relationships
Source: PLoS One. 2015 Jul 30;10(7):e0133602. doi: 10.1371/journal.pone.0133602 (PMC4520491; doi:10.1371/journal.pone.0133602)

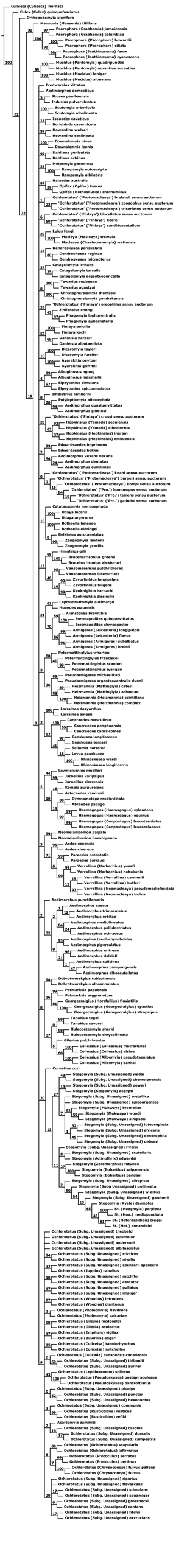

Supplement: S1 Tree — (PDF) [file pone.0133602.s005.pdf]

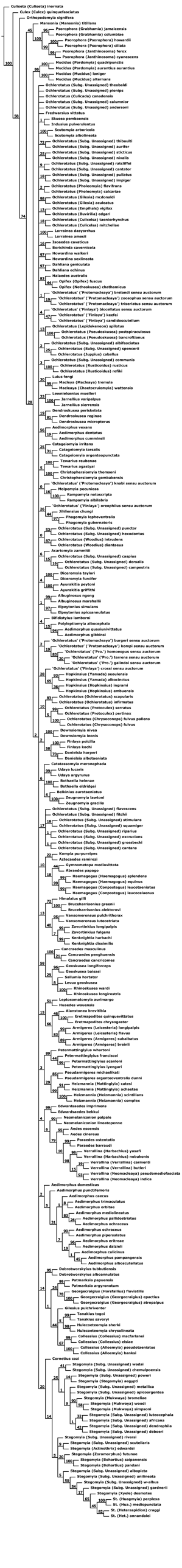

Supplement: S2 Tree — (PDF) [file pone.0133602.s006.pdf]

Aedini, 14 characters ordered, strict consensus of 4000 MPTs, L=6654

Strict consensus tree

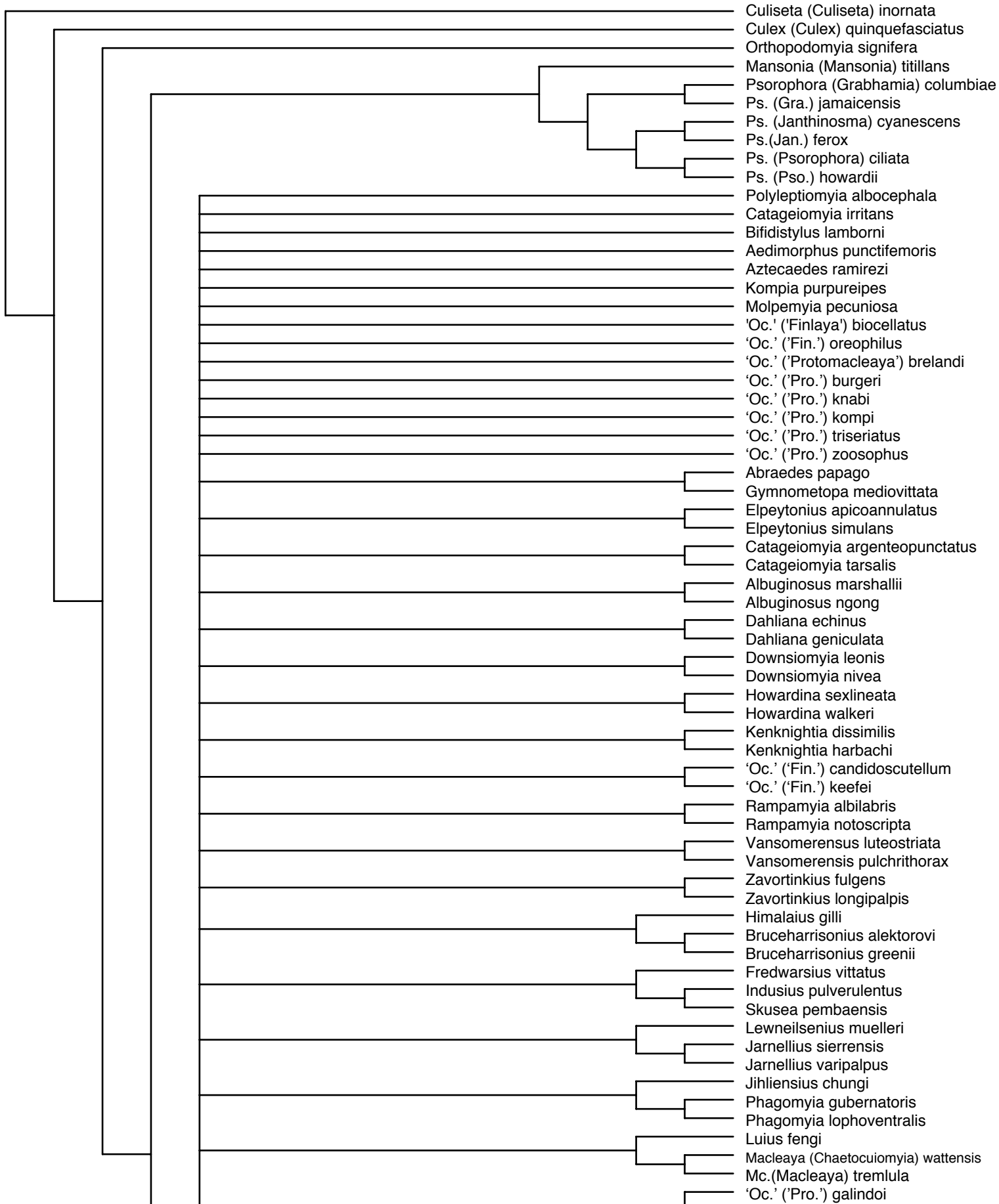

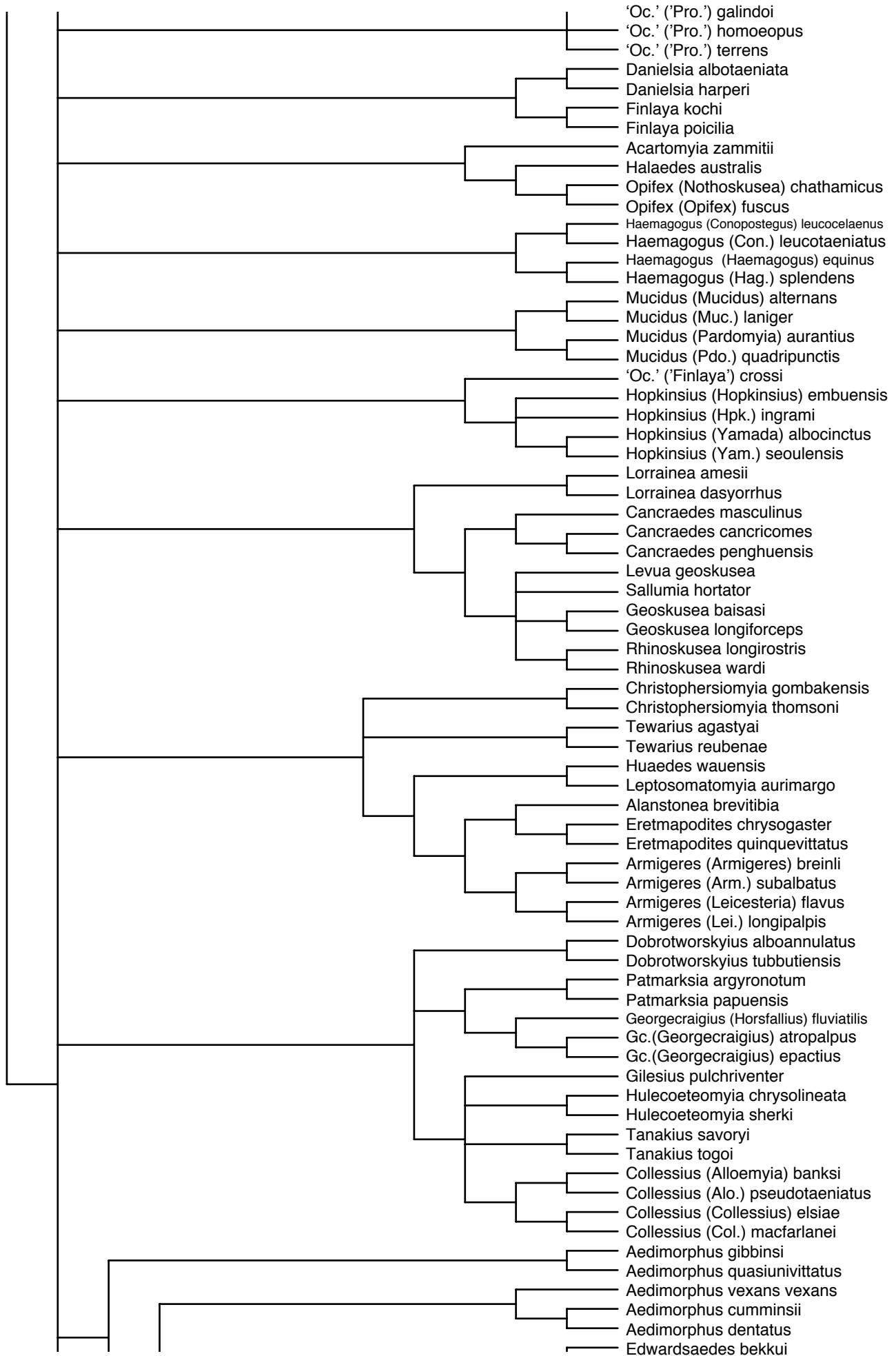

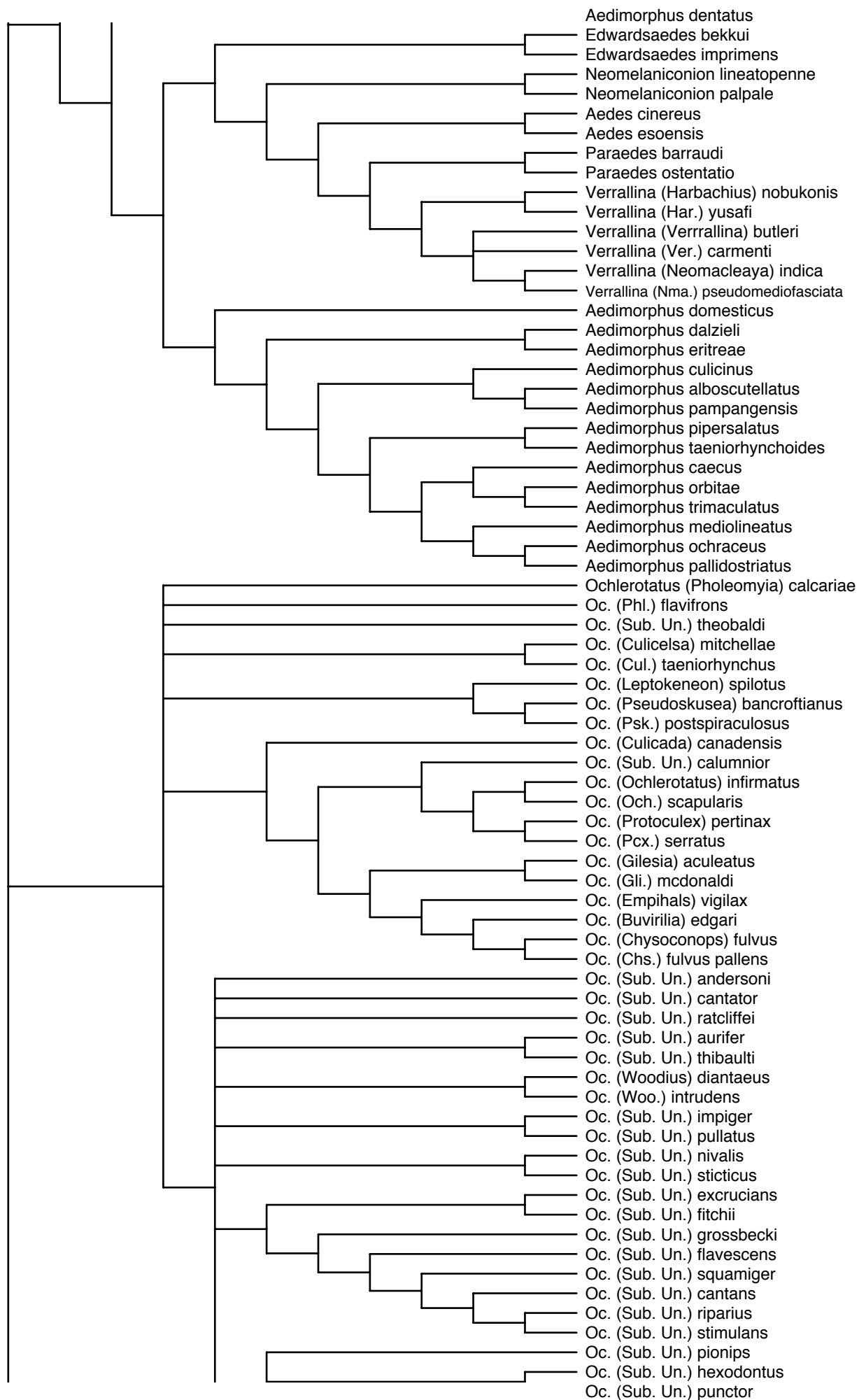

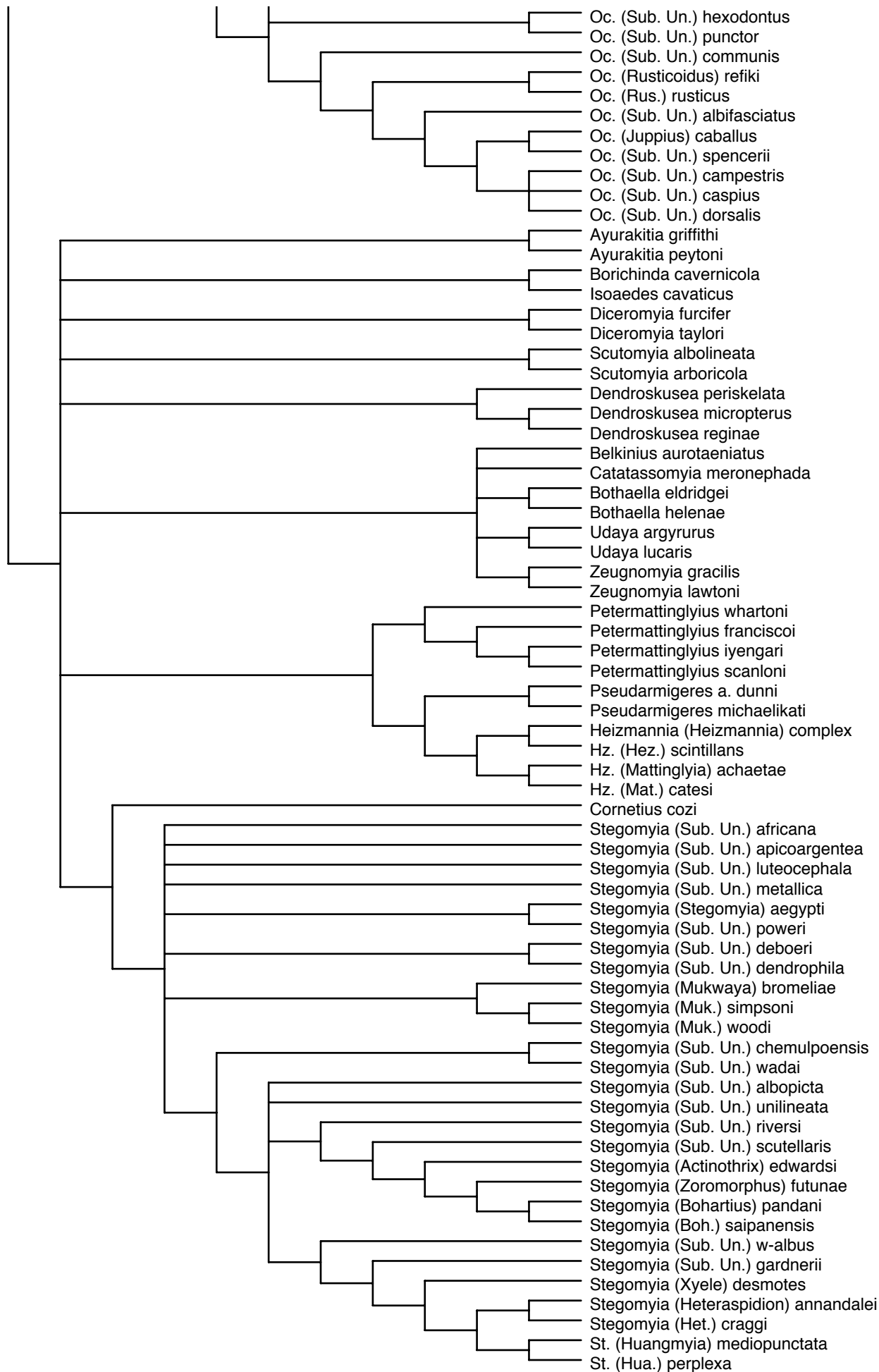

Supplement: S3 Tree — (PDF) [file pone.0133602.s007.pdf]

Aedini, all characters unordered, strict consensus of 832 MPTs, L=6609

Strict consensus tree

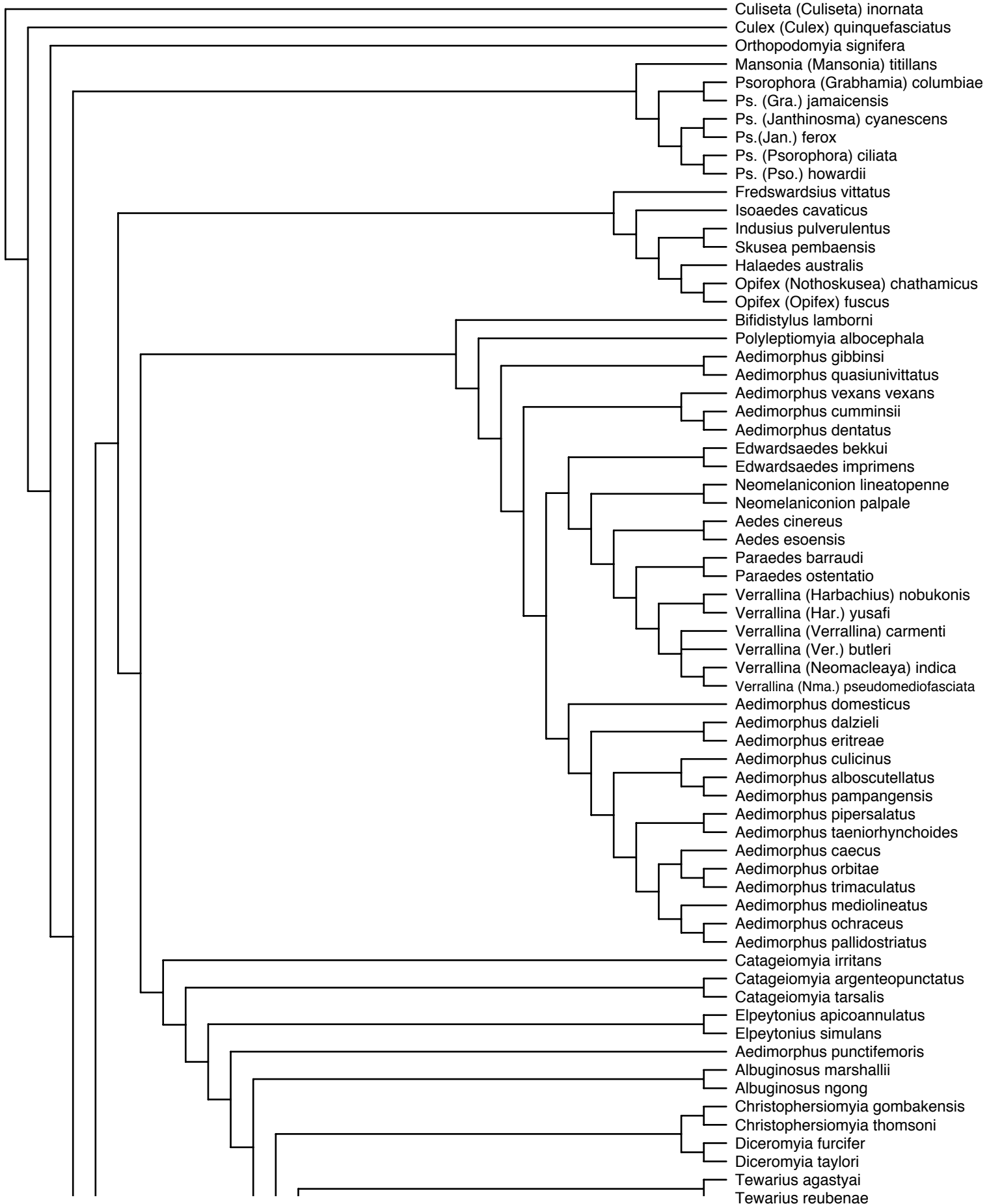

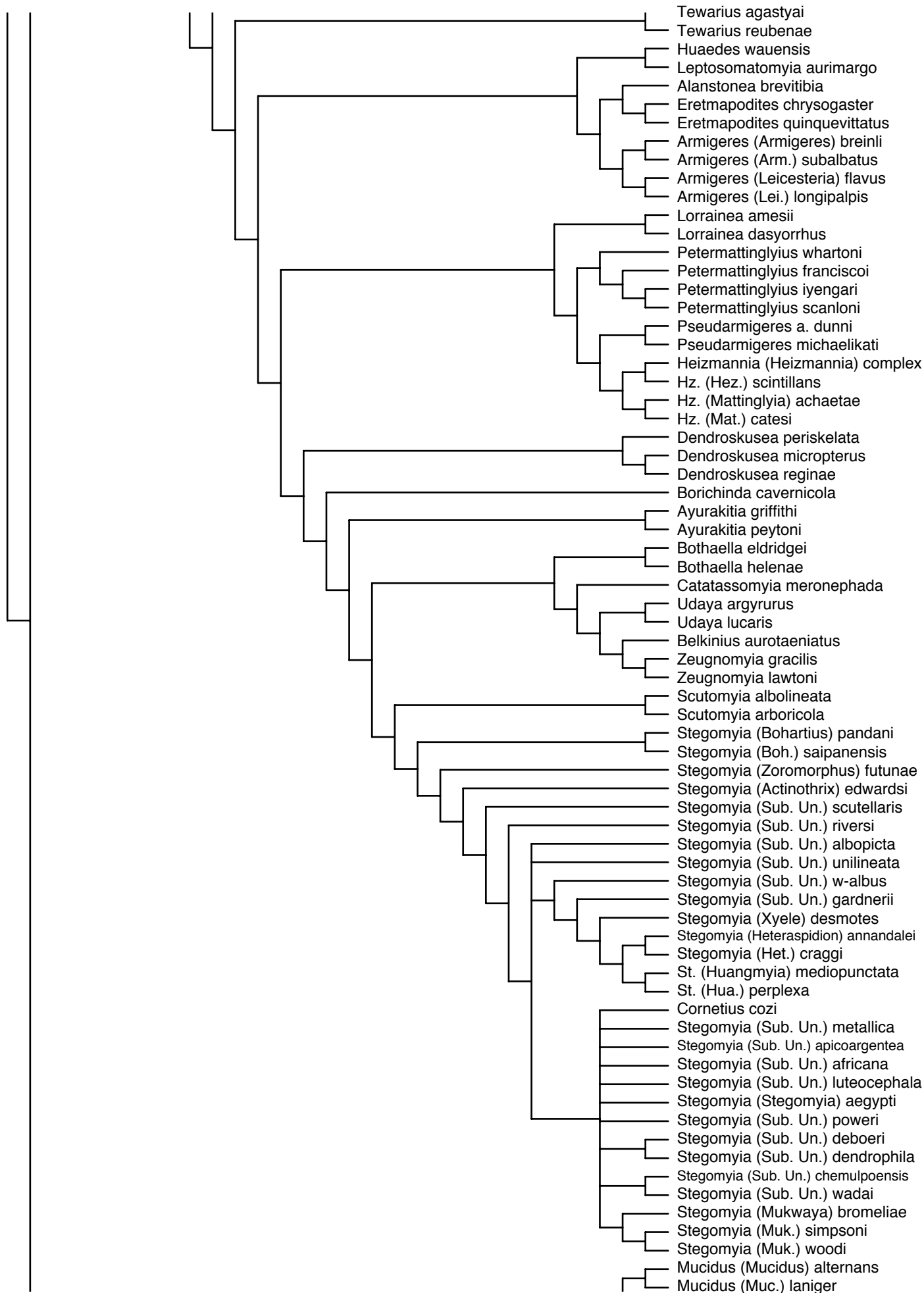

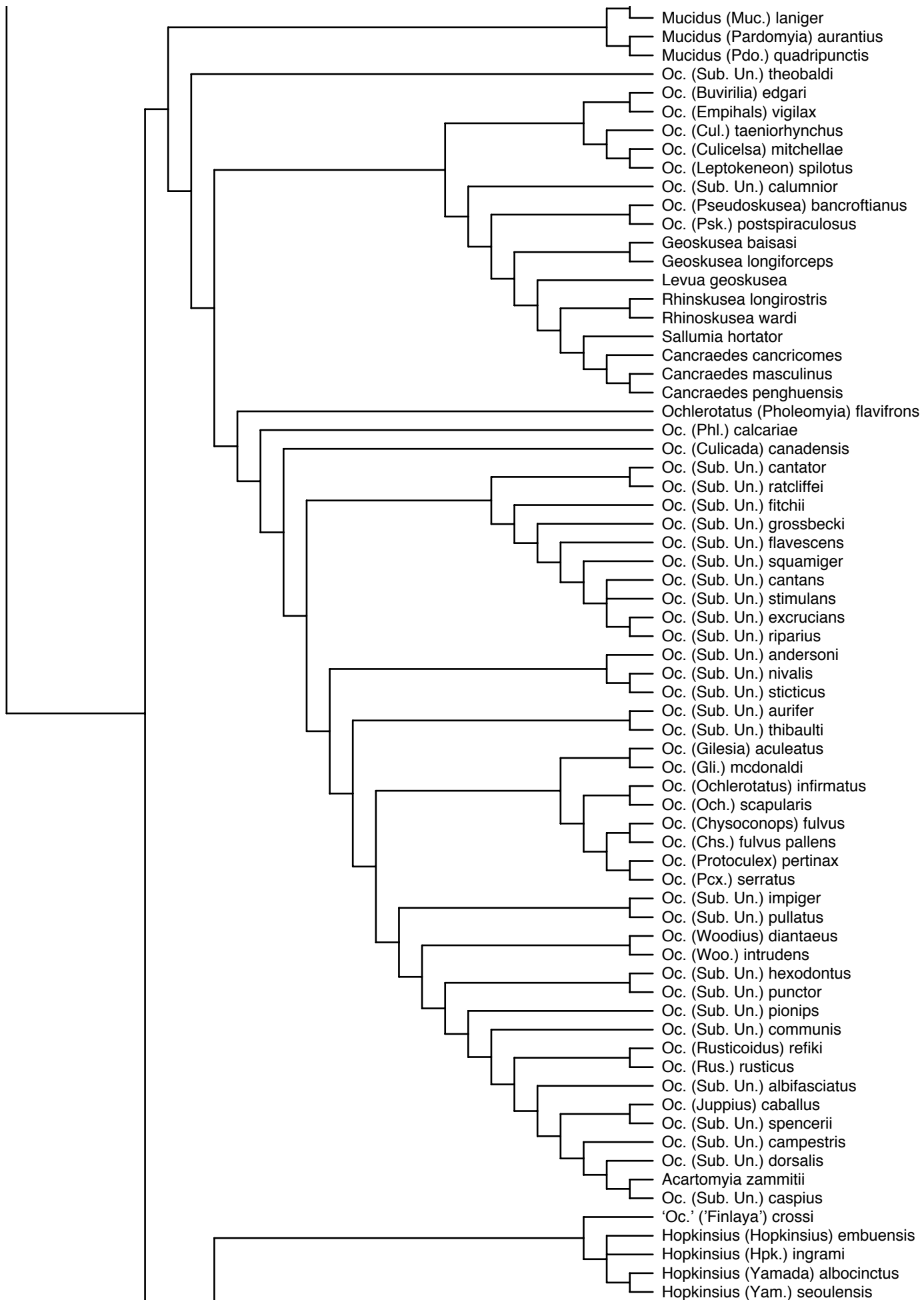

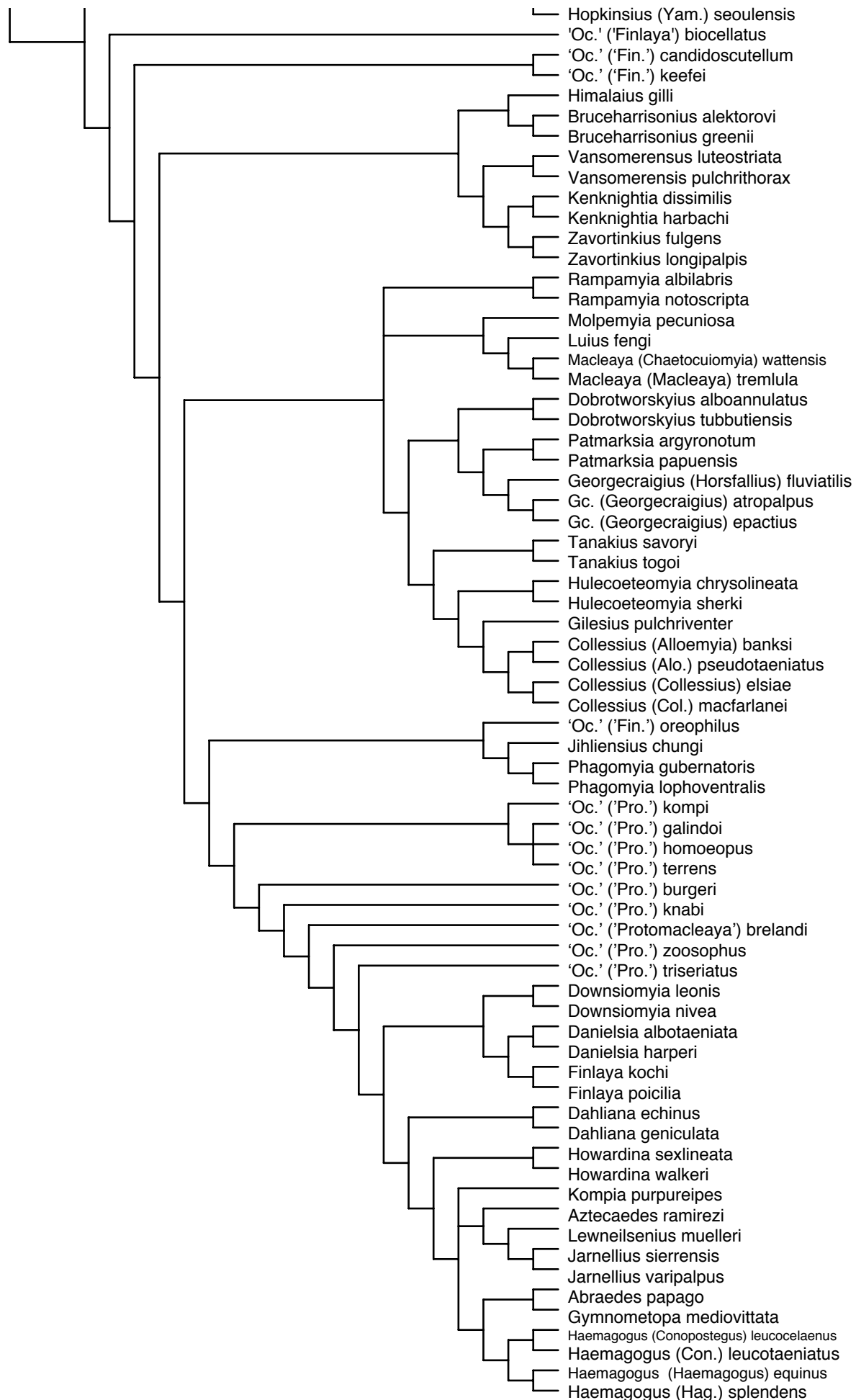

Supplement: S4 Tree — (PDF) [file pone.0133602.s008.pdf]
